# Mini-TCRs: Truncated T cell receptors to generate T cells from induced pluripotent stem cells

**DOI:** 10.1016/j.omtm.2023.101109

**Published:** 2023-09-16

**Authors:** Shin-ichiro Takayanagi, Bo Wang, Saki Hasegawa, Satoshi Nishikawa, Ken Fukumoto, Kohei Nakano, Sayaka Chuganji, Yuya Kato, Sanae Kamibayashi, Atsutaka Minagawa, Atsushi Kunisato, Hajime Nozawa, Shin Kaneko

**Affiliations:** 1 Kirin Central Research Institute, Kirin Holdings Company, Ltd., 26-1, Muraoka-Higashi 2, Fujisawa-shi, Kanagawa 251-8555, Japan; 2 Shin Kaneko Laboratory, Department of Cell Growth and Differentiation, Center for iPS Cell Research and Application (CiRA), Kyoto University, 53 Kawahara-cho, Shogoin, Sakyo-ku, Kyoto 606-8507, Japan; 3 R&D Division, Kyowa Kirin Co. Ltd, 3-6-6 Asahi-machi, Machida-shi, Tokyo 194-8533, Japan; 4 Shinobi Therapeutics, Inc., 46-29 Yoshida-Shimo-Adachi-cho, Sakyo-ku, Kyoto 606-8501, Japan

**Keywords:** induced pluripotent stem cell, T-cell receptor, cytotoxic T lymphocyte, chimeric antigen receptor T cell, antigen recognition, mini-TCR, graft-versus-host disease

## Abstract

Allogeneic T cell platforms utilizing induced pluripotent stem cell (iPSC) technology exhibit significant promise for the facilitation of adoptive immunotherapies. While mature T cell receptor (TCR) signaling plays a crucial role in generating T cells from iPSCs, the introduction of exogenous mature TCR genes carries a potential risk of causing graft-versus-host disease (GvHD). In this study, we present the development of truncated TCRα and TCRβ chains, termed mini-TCRs, which lack variable domains responsible for recognizing human leukocyte antigen (HLA)-peptide complexes. We successfully induced cytotoxic T lymphocytes (CTLs) from iPSCs by employing mini-TCRs. Combinations of TCRα and TCRβ fragments were screened from mini-TCR libraries based on the surface localization of CD3 proteins and their ability to transduce T cell signaling. Consequently, mini-TCR-expressing iPSCs underwent physiological T cell development, progressing from the CD4 and CD8 double-positive stage to the CD8 single-positive stage. The resulting iPSC-derived CTLs exhibited comparable cytokine production and cytotoxicity in comparison to that of full-length TCR-expressing T lymphocytes when chimeric antigen receptors (CARs) were expressed. These findings demonstrate the potential of mini-TCR-carrying iPSCs as a versatile platform for CAR T cell therapy, offering a promising avenue for advancing adoptive immunotherapies.

## Graphical abstract

**Figure:**
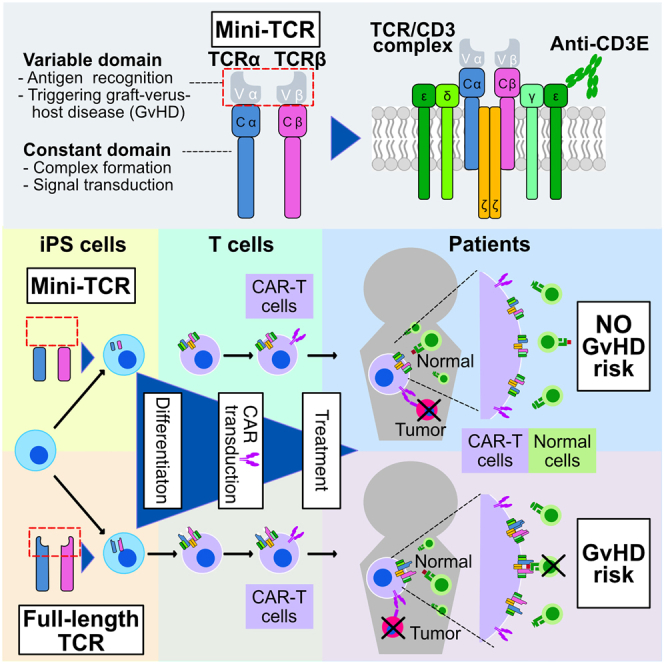

## Teaser

Kaneko and colleagues have pioneered the novel artificial T cell receptor, termed mini-TCR. Although lacking antigen-recognition capacity, mini-TCR facilitates the generation of CAR T cells from iPSCs. This innovative technology holds promise for broadening therapeutic avenues in future cancer therapy.

## Introduction

Recent advances in cancer immunotherapies have extended existing therapeutic options such as surgery, chemotherapy, and radiotherapy for patients.^1^^,^^2^ In particular, CAR T cell therapies have shown clinical outcomes in patients with B cell malignancies.^3^^,^^4^ However, current autologous CAR T cell therapies have issues, such as a time lag until treatment and variability in population, that can occasionally lead to treatment failures.^5^^,^^6^^,^^7^^,^^8^ A promising solution to these problems is to use allogeneic T cells from a healthy donor source and provide them in an off-the-shelf style.^9^^,^^10^

In addition to using donor-derived T cells, technologies based on feeder-free differentiation of induced pluripotent stem cells (iPSCs) have found clinical application and could become a mainstream adoptive immunotherapy in the future.^9^^,^^11^^,^^12^^,^^13^ Both iPSC-derived and peripheral-blood-derived CAR T cells demonstrate comparable antitumor efficacy in mouse models.^14^ Undifferentiated iPSCs are expandable, and CAR-expressing iPSCs can proliferate and be established as master cells.^15^ Furthermore, gene editing against human leukocyte antigen (HLA) genes can universalize master cells to all patients.^16^ Therefore, we focused on iPSC-derived T cells as host cells for immunotherapies.

Physiologically, T cell receptors (TCRs) play a crucial role in the immune system by interacting with antigen-presenting cells (APCs) and are involved in T cell development.^17^ Several groups have created T cell-derived iPSCs (T-iPSCs) that express mature TCR genes possessed by T cells and that have the potential to differentiate into T cells.^18^^,^^19^ Additionally, overexpression of full-length TCR genes into non-T cell-derived iPSCs is sufficient to generate T cells.^20^ However, cross-reactivity of endogenous or overexpressed TCRs on these allogeneic T cells could be a risk factor for graft-versus-host disease (GvHD),^10^ and the cross-reactivity of a TCR specific for HLA-A∗24:02/Wilms tumor 1(235–243) with HLA-B∗57:01 has been reported.^21^

In this study, we designed "mini-TCRs" comprising truncated TCRα and TCRβ that lacked variable regions but maintained TCR functions. These mini-TCRs recruited CD3 proteins to the cell surface and transduced T cell activation signals. Mini-TCR expression induced differentiation to CD8+ T cells from undifferentiated iPSCs and iPSC-derived hematopoietic stem/progenitor cells (HSPCs). The generated CD8+ T cells also demonstrated specific cytotoxicity of target cells when used for engineering CAR T cells. We propose this technology as a promising tool for developing allogeneic, off-the-shelf CAR T cell therapies.

## Results

### Screening identified a mini-TCR with high CD3 recruitment activity

**Figure 1:**
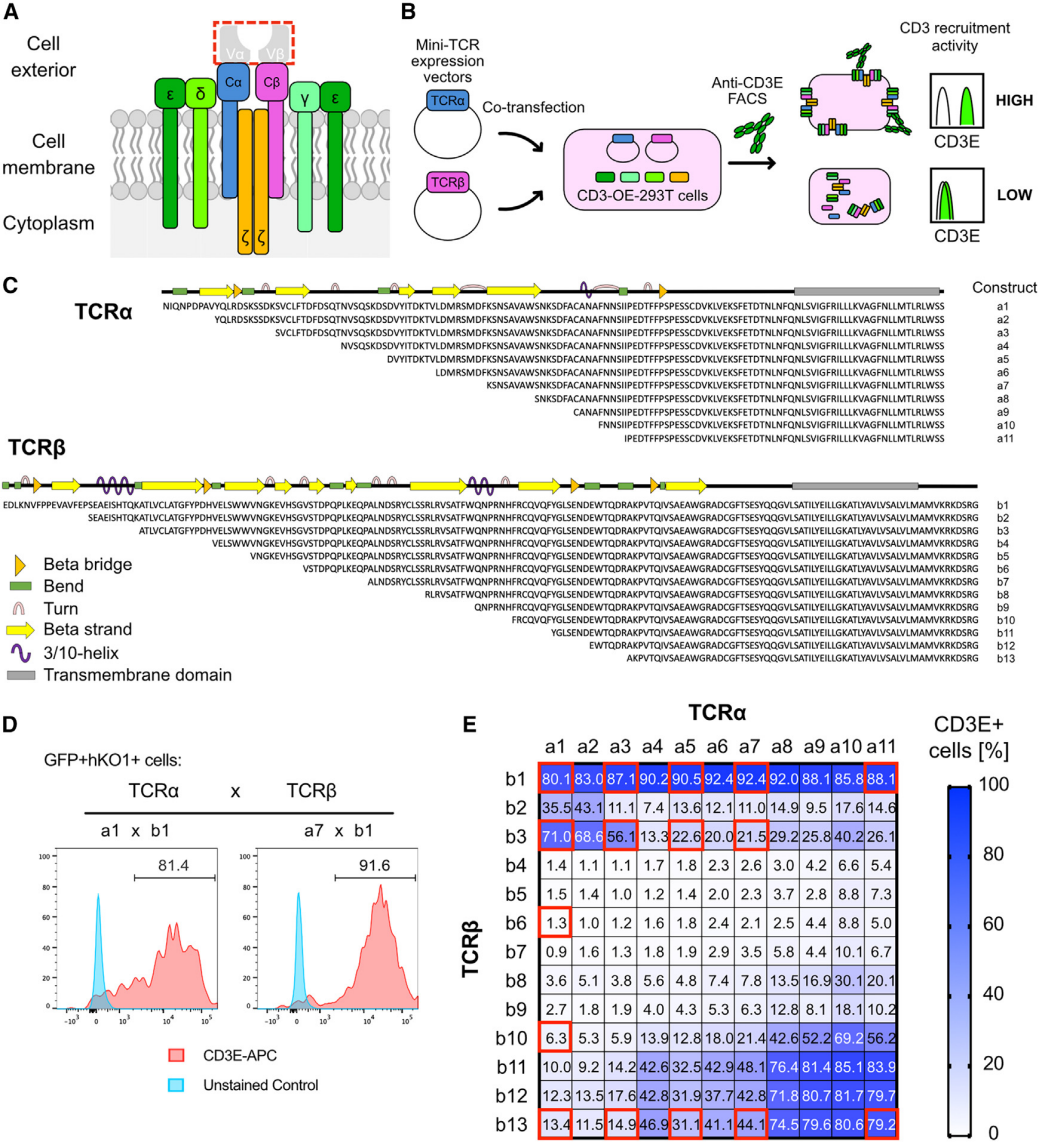
Design of mini-TCR constructs and their CD3 protein recruitment activities (A) Schematic representation of the TCR/CD3 complex. The deletion of variable regions from TCRα and TCRβ chains depicts the concept of mini-TCR. Cα, constant region of TCRα; Cβ, constant region of TCRβ; Vα, variable region of TCRα; Vβ, variable region of TCRβ; ε, CD3E; γ, CD3G; δ, CD3D; and ζ, CD3Z. (B) The experimental scheme to screen the pairs of TCRα and TCRβ chains based on the CD3 recruitment activity. (C) Amino acid sequences and protein motifs of constant regions of TCRα (upper) and TCRβ (lower) chains. The constant regions were tagged with Myc (TCRα, TCRA-Myc) or FLAG (TCRβ, TCRB-FLAG) epitopes at the C terminus. (D) Representative plots of CD3-OE-293T cells transfected with TCRA-Myc and TCRB-FLAG. hKO1+GFP+ cells are shown. (E) The screening result of TCR constructs based on their CD3 protein recruitment activities. Values in the heatmap indicate the percentage of CD3E+ cells. Data are shown as mean of three independent experiments.

In the immunological synapse (the interface between T cells and APCs), TCRs form complexes with CD3 proteins and recognize HLA-antigen peptide complexes on APCs.^17^^,^^22^ TCR proteins contain variable domains that determine the specificity of the TCR and constant domains to interact with CD3 proteins.^23^ We designed several TCRs lacking the variable region that did not recognize antigens but that did transduce TCR signaling, and we designated them mini-TCRs (Figure 1A).

To identify the mini-TCRs that efficiently form complexes with CD3 proteins, we created mini-TCR libraries comprising various lengths of the constant regions of the TCRα and TCRβ chains and designed the cell-based screening system to measure the cell surface localization of the CD3 protein (Figure 1B). The N termini were selected mainly at the boundaries of motifs and domains based on previous studies^24^^,^^25^^,^^26^^,^^27^ and protein crystal structures (Protein DataBank: 3QJF) (Figure 1C).^28^ A signal peptide was added to the 5′ terminus of the designed TCR sequences that were tagged with Myc (for TCRα, TCRA-Myc) or FLAG (for TCRβ, TCRB-FLAG) epitopes at the 3′ terminus (Figure S1A). Intracellular staining with anti-Myc or anti-FLAG antibodies confirmed the expression of TCRA-Myc or TCRB-FLAG proteins from all vectors (Figures S1B and S1C, respectively). We then co-transfected all combinations of generated TCRA-Myc and TCRB-FLAG vectors into 293T cells expressing all CD3 genes (CD3-OE-293T cells; Figure S1D) to test whether they could translocate CD3 proteins to the cell surface (hereafter referred to as CD3 recruitment activity) (Figure 1B). CD3 recruitment activity was found to differ depending on the combination of TCRα and TCRβ (Figures 1D and 1E). Particularly high CD3 recruitment activities were observed when the full-length constant region of TCRβ was used irrespective of the length of a coupled TCRα, as well as in the case where both TCRα and TCRβ were short, truncated forms. The highest CD3 recruitment activity was achieved by combining α7 and β1 (92.4%). These results showed that even TCRs lacking the variable region can recruit CD3 proteins to the cell surface.

**Figure 2:**
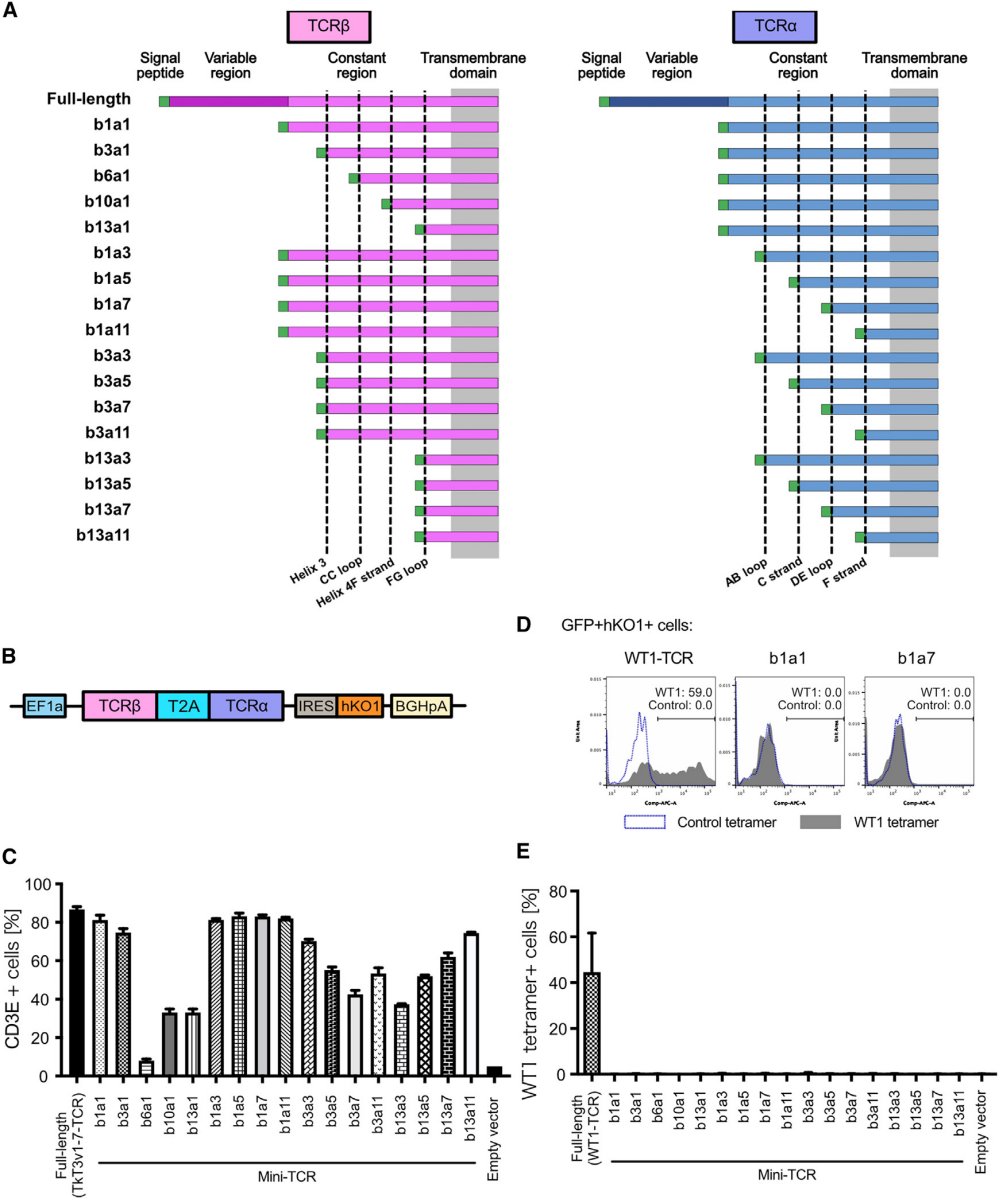
CD3 recruitment activities of mini-TCRs using a polycistronic expression vector (A) Structures of the TCRα and TCRβ proteins in the generated vector. (B) The vector structure that combines TCRα and TCRβ into a single vector. The humanized Kusabiraorange1 (hKO1) fluorescent protein was used as a transfection marker. T2A, Thosea asigna virus 2A-like peptide. (C) CD3 protein recruitment activities of each created vector expressed in CD3-OE-293T cells. Data are shown as mean ± SD of three independent experiments. (D) Representative plots of TCR-transfected CD3-OE-293T cells stained with the Wilms Tumor1 (WT1)-TCR tetramer. (E) Mean fluorescence intensity (MFI) of the WT1-TCR tetramer. Data are shown as mean ± SD of three independent experiments.

Based on CD3 recruitment capacities, we selected 17 representative pairs of truncated TCRα and TCRβ, including the pairs with the lowest (β6 and α1) and highest (β1 and α7) recruitment activities (Figures 1E and 2A). cDNAs of the selected TCRβ and TCRα were fused with the T2A peptide as previously reported and were subcloned into a single vector, as well as full-length TCRα and full-length TCRβ (Figures 2A and 2B).^29^ The CD3 recruitment activities of the generated constructs in CD3-OE-293T showed a similar trend to that of the mini-TCRs that were transfected with separate TCRα and TCRβ expression vectors (Figure 2C).

To confirm the loss of antigen-recognition abilities due to deletion of the variable regions, we transfected the 17 pairs of mini-TCR vectors and the full-length TCR that recognized mutated Wilms-Tumor1 (WT1) peptide-HLA-A∗24:02 complex as a positive control into CD3-OE-293T cells. In contrast to the binding of WT1-TCR-expressing cells to the WT1-HLA-A∗24:02 tetramer, none of the mini-TCR-expressing cells exhibited binding to the tetramer, confirming that the mini-TCR did not recognize the antigen peptide-HLA complexes (Figures 2D and 2E). Based on these initial assessments, we used these 17 pairs of mini-TCR for further characterization.

### Mini-TCR activated Jurkat T cells upon CD3 stimulation

TCR/CD3 complexes transduce TCR signaling and activate T cells.^30^ Because 293T cells are not derived from T cells, TCR signaling-related molecules, including the CD3 genes, are considered to be negative. Therefore, we used human T cell-derived Jurkat cells to monitor activation upon CD3 stimulation as the second screening step.

To specifically detect transfected TCR proteins, we attempted to disrupt endogenous TCRA and TCRB genes using gene-editing technology. Both TCRα constant (TRAC) locus and TCRβ constant 1 (TRBC1) loci were targeted with previously reported guide RNAs (gRNAs) (Figure S2A). To confirm the disruption of the TCR genes, full-length TCRα and/or full-length TCRβ expression vectors were transfected into Jurkat cells, which were subsequently stained with an anti-CD3E antibody and an anti-TCRαβ antibody that recognized a part of the constant region of the TCRαβ complex. Although the CD3E and TCRαβ protein was detected after co-transfection of both full-length TCRα and full-length TCRβ, they were not detected by either single transfection of full-length TCRα or full-length TCRβ, suggesting that endogenous TCR genes were successfully disrupted (Figure S2B).

**Figure 3:**
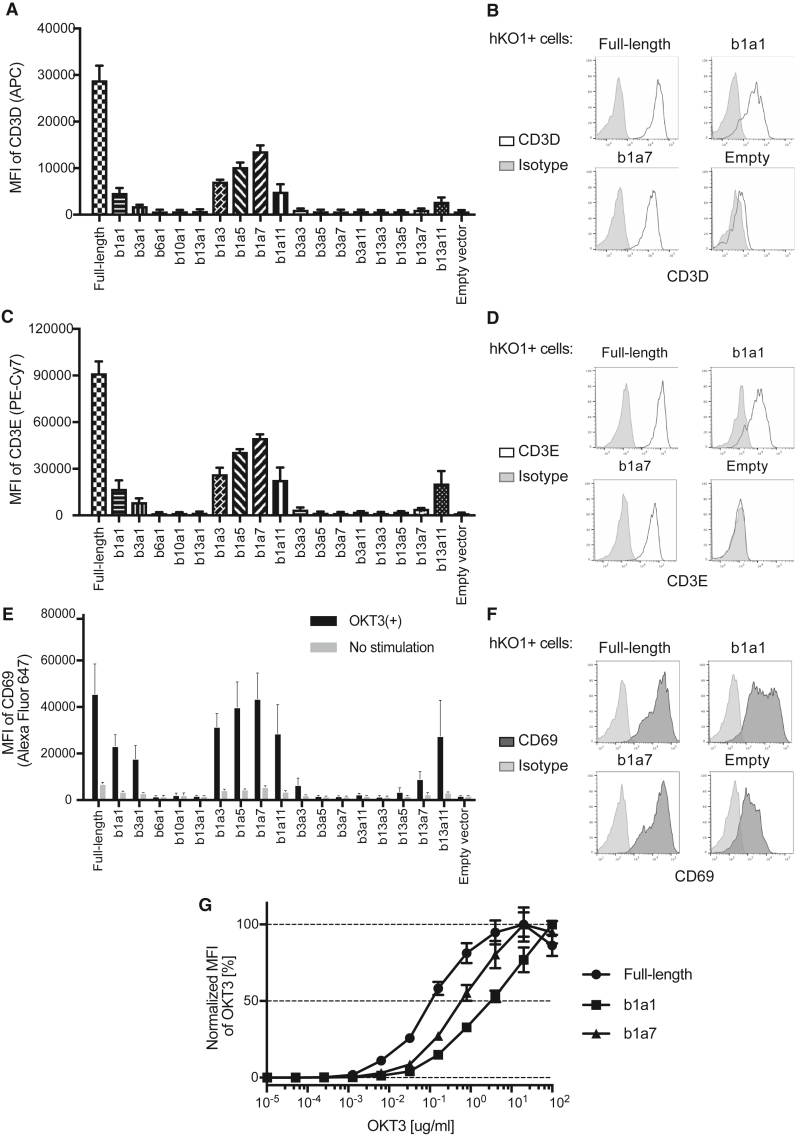
Activation of mini-TCR-expressing TCR-KO Jurkat cells via CD3 stimulation MFI (A) and representative plots (B) of CD3D in TCR-KO Jurkat cells transiently transfected with TCR expression vectors. MFI (C) and representative plots (D) of CD3E in TCR-KO Jurkat cells transiently transfected with TCR expression vectors. MFI (E) and representative plots (F) of the CD69 T cell activation marker. TCR-KO Jurkat cells transiently transfected with TCR expression vectors were stimulated with anti-CD3E antibody OKT3 overnight and stained with the anti-CD69 antibody. (G) Normalized MFI of OKT3 in TCR-KO Jurkat cells transiently transfected with TCR expression vectors to compare the reactivity of OKT3. All data are shown as mean ± SD of three independent experiments.

Subsequently, to compare CD3 recruitment activity, we transfected all 17 mini-TCR vectors, the full-length TCR control, and the empty vector as a negative control. Neither CD3D nor CD3E proteins were detected in the empty-vector control samples, confirming that CD3D and CD3E proteins did not localize on the cell surface unless the TCRA and TCRB chains were expressed in the Jurkat knockout (KO) cells (Figures 3A–3D). Cell surface localization of CD3D and CD3E proteins was detected in TCR-KO Jurkat cells with full-length TCR controls and several mini-TCRs, including b1a1 and b1a7, suggesting that these mini-TCRs formed a complex with CD3 proteins (Figures 3A–3D).

To assess the function of the mini-TCR/CD3 complex, the transfected TCR-KO Jurkat cells were stimulated with an OKT3 anti-CD3E antibody. Subsequently, we observed the highest upregulation of CD69, a T cell activation marker, in the full-length TCR control, and the mini-TCR b1a7 showed comparable upregulation, followed by other mini-TCRs that showed correlation with mean fluorescence intensities (MFIs) of CD3D and CD3E (Figures 3E, 3F, and S2C).

As the binding affinity of the anti-CD3E antibody OKT3 to TCR/CD3 complex measured by bio-layer interferometry was approximately 10-fold higher than that of the extracellular domains of CD3γε,^27^ suggesting that the presence of the TCR proteins affected the activity of the OKT3 antibody, we hypothesized that CD69 expression levels were affected by the different binding capacities of OKT3 to the full-length TCR-CD3 and mini-TCR-CD3 complexes on the cell surface. To confirm this, mini-TCR (b1a7) (which showed the highest CD3 recruitment activity and CD69 expression among the mini-TCRs), mini-TCR (b1a1) (composed of the full-length TCRα and TCRβ constant regions with lower activities), and full-length TCR were each transfected into the TCR-KO Jurkat cells. The full-length TCR-expressing cells exhibited the highest MFI overall, followed by b1a7-and b1a1-expressing cells (Figure S2D), which correlated with the MFI of CD3E detected by anti-CD3E antibody UCHT1 (Figure 3C). Based on the normalized MFI of OKT3, the highest reactivity was observed against full-length TCR, followed by that against b1a7 and b1a1, suggesting the relatively low affinity of OKT3 against mini-TCRs (Figure 3G).

Collectively, these data demonstrated that the introduction of mini-TCRs enables endogenous CD3 proteins to localize on the cell surface and mediate TCR signaling via stimulation with the CD3 antibody. Subsequently, we evaluated the functions of mini-TCRs b1a1 and b1a7 in the differentiation of iPSCs to T cells.

### Mini-TCRs enabled T cell differentiation of iPSC-derived HSPCs

The TCR/CD3 complex is also critical for T cell development. The T cell lineage commitment of common lymphoid progenitors derived from multipotent HSPCs is triggered by Notch signaling.^31^ CD4 and CD8 double-negative (DN) T cells mature into double-positive (DP) cells, followed by further maturation into single-positive (SP) CD4 or SP CD8 cells in the thymus.^32^ During maturation, T cells are selected based on their binding affinities between the self-peptide-HLA complex on thymic epithelial cells and the TCR/CD3 complex.^33^

**Figure 4:**
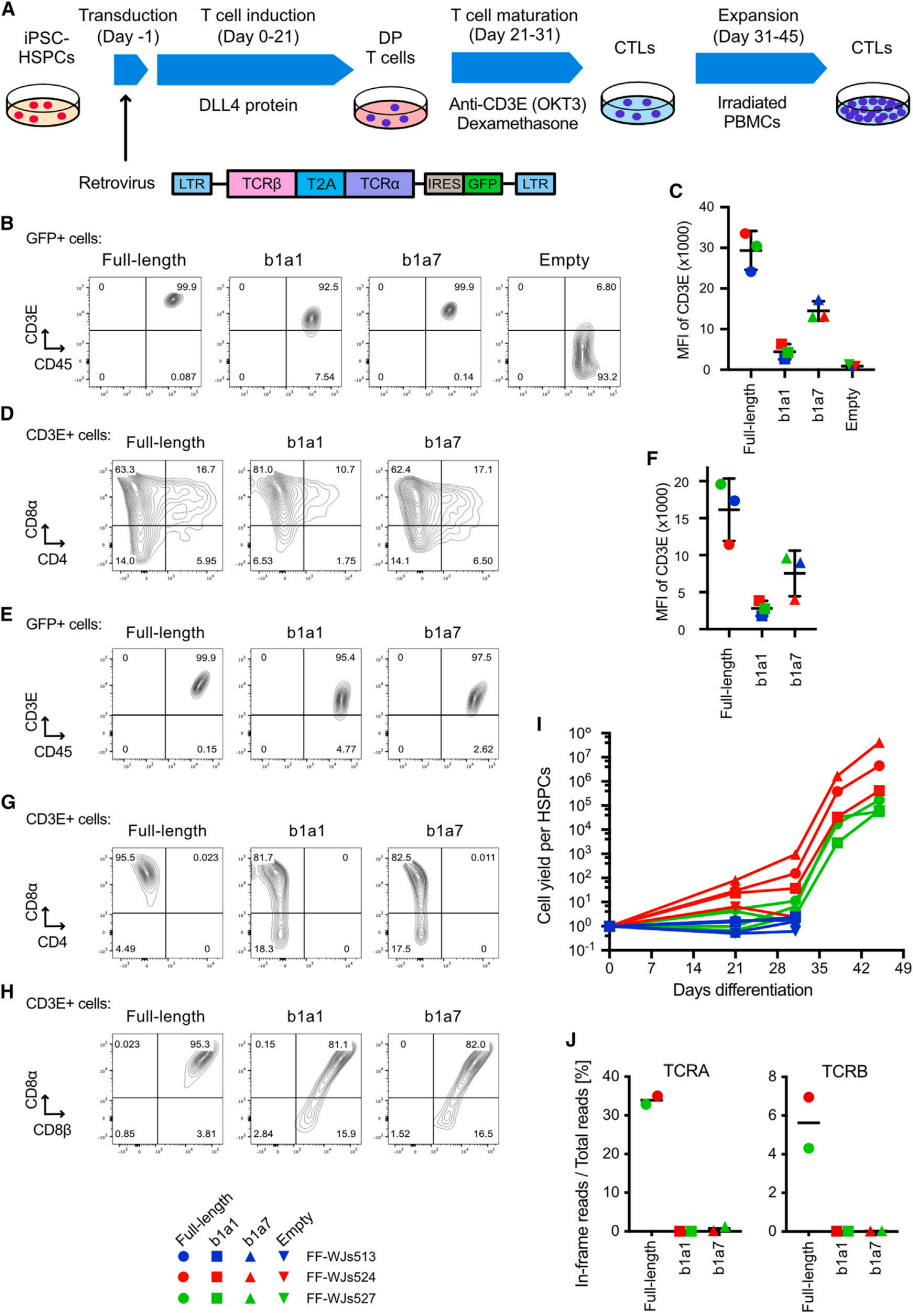
Induction of CTLs from iPSC-derived HSPCs by mini-TCRs (A) Experimental scheme of T cell differentiation using TCR genes. For the stepwise differentiation processes from iPSC-derived HSPCs to CTLs, retroviruses encoding full-length TCR, mini-TCR, or empty control vectors were transduced into HSPCs. (B) Representative FACS plots of the CD45 and CD3E expression after T cell induction on DLL4 protein. (C) MFI of CD3E in GFP+ transduced cells after T cell induction on DLL4 protein. Data are shown as mean ± SD of three iPS clones. (D) Representative FACS plots of CD4 and CD8α in CD3E + cells after T cell induction. (E) Representative FACS plots of the CD45 and CD3E expression after T cell maturation using the anti-CD3E antibody and dexamethasone. (F) MFI of CD3E in GFP+ cells after T cell maturation. Data are shown as mean ± SD of three iPS clones. (G) Representative FACS plots of CD4 and CD8α expression after T cell maturation. (H) Representative FACS plots of CD8α and CD8β expression after T cell maturation. (I) Estimated cell yields per HSPC. Cell numbers were calculated based on the initially seeded HSPC count and subsequent proliferation rates. (J) TCR gene expression analysis by NGS. Percentages of in-frame reads coding CDR3 amino acid sequence derived from endogenous TCRA or TCRB genes are shown.

We utilized cord-blood-derived iPSCs as a model of the hematopoietic system to test the function of mini-TCRs in T cell development, as these iPSCs were derived from myeloid cells and their TCR genes were not rearranged (Figure 4A). First, the iPSCs were differentiated into HSPCs via an embryoid body formation method, and retroviruses encoding full-length TCR, mini-TCRs b1a1 or b1a7, or empty-vector control were transduced into the harvested HSPCs (day −1; Figure S3A). The transduced cells were then cultured with a recombinant Delta-like ligand 4 (DLL4) protein, a ligand of Notch receptors, to trigger lineage commitment into T cells (days 0–21). There are no statistically significant differences in the MFI of GFP, which indicate transduced vector copy numbers, across the four retroviruses (Figure S3B).^34^ Flow cytometry analyses showed that cell surface expression of CD3 was observed only in full-length TCR- and mini-TCR (b1a1 and b1a7)-transduced cells, whereas leukocyte common antigen CD45 was expressed in all samples, including empty-vector control cells (day 21; Figure 4B). Full-length TCR-transduced cells showed the highest MFI of CD3 (MFI = 29,377), followed by b1a7 (MFI = 14,483), b1a1 (MFI = 4,423), and the empty-vector control (MFI = 898; Figure 4C). Both full-length TCR and mini-TCR-expressing CD3+ cells included comparable percentages of CD4+CD8α+ DP cells, while the DP cells were not enriched (Figure 4D). These data indicated that introduction of mini-TCR genes enables efficient differentiation of iPSC-derived HSPCs into T cell lineage cells.

T cell activation signaling via the TCR/CD3 complex is essential for the maturation of DP cells to SP cells in the thymus.^33^ To test the function of CD3E proteins on the mini-TCR-expressing DP cells during the maturation process, entire T cells, including DP cells, were further stimulated with an OKT3 anti-CD3 antibody in the presence of dexamethasone, a steroid that mimics endogenous glucocorticoids in the thymus (Figure 4A).^35^^,^^36^ Selective activation of CD3+ cells led to nearly 100% enrichment of GFP+ cells (Figure S3C). The order of the MFI of CD3E on full-length- and mini-TCR-transduced cells after OKT3 stimulation was similar to that of DP T cells (Figures 4E and 4F). Notably, viable cell populations were not observed in empty-vector control samples after maturation (data not shown; no cell populations were observed in the forward scatter-side scatterplot of the flow cytometry analysis). Neither mini-TCR- nor full-length TCR-transduced cells expressed CD4 (Figure 4G); however, both expressed CD8α and CD8β, phenotypic markers of authentic cytotoxic T lymphocytes (CTLs; Figure 4H). These CTLs were expandable when peripheral blood mononuclear cells (PBMCs) were co-cultured as feeder cells, and entire cell yields per HSPC reached at least 10^5^-fold (Figure 4I).

Avoiding GvHD risk is one of the primary purposes of the mini-TCRs. While the loss of antigen-recognition capacities by mini-TCR was experimentally demonstrated in Figure 2E, endogenous expression of TCRA and TCRB genes in the differentiated CTLs remains the risk associated with GvHD. We performed next-generation sequencing (NGS) of TCRA and TCRB transcripts, of which cDNA samples were amplified with the adapter primer at the 5′ terminus, and primers correspond to constant regions (Figure S4A). Then, sequenced reads were queried to the CDR3 sequence database to extract in-framed reads that can be translated to CDR3 proteins. It is worth noting that mini-TCR lacks variable domains, including CDR3, and the detected TCRA and TCRB transcripts could indicate endogenous TCR gene expression in mini-TCR-transduced CTLs. Among the nearly 3 × 10^5^ total reads, 33.9% of in-frame TCRA reads and 4.95% of in-frame TCRB were detected in full-length TCR CTLs (mean values of two iPSCs) (Figure 4J). Mini-TCR CTLs exhibited very limited frequencies of in-frame TCRA reads (0.01%–1.29%) and in-frame TCRB reads (0.01%–0.03%) in both b1a1 and b1a7 mini-TCR CTLs (Figure 4J).

To determine the endogenous TCR gene expression, we referred to our previous work that demonstrated the disruption of RAG2-disabled rearrangement of TCR genes during T cell induction from two T cell-derived iPSCs (GPC3 T-iPSC and TkT3V1-7) using similar NGS-based TCR repertoire analysis with the similar depth of the sequencing (3.3–4.9 × 10^5^ total reads) (Table S1).^36^ Among the 1.8 or 2.5 × 10^5^ total in-frame reads, 86.31% and 91.85% of the reads represented the original TCRA sequences in the RAG2-KO iPSC-derived T cells (GPC3 T-iPSC and TkT3V1-7, respectively) (Table S1), whereas only 1,000 to hundreds of reads encoded CDR3 amino acid sequences other than the original TCRA (Table S1, rank 2–5). Considering that RAG genes are vital for TCR rearrangement, hundreds of in-frame reads other than the original TCRs were regarded as sequencing noise levels. In this study, 96.47% and 93.22% of in-frame reads from full-length TCR CTLs were confirmed to be those of transduced full-length TCRA and TCRB, respectively (Tables S1, S2, and S3). Regarding the mini-TCR-transduced CTLs, the numbers of in-frame reads were also less than hundreds, except for b1a7-transduced FF-WJs527 cells, suggesting that mini-TCR suppressed the expression of endogenous TCR to the near noise level. Because CTLs induced with b1a7 exhibited a tendency toward a biased repertoire (Figure S4B; Tables S1 and S2), disrupting the RAG2 gene or TRAC locus would be worth considering to avoid the TCR rearrangement and endogenous TCR expression altogether. In conclusion, mini-TCRs demonstrated comparable performance to full-length TCR as inducers of phenotypic CTLs in iPSC-derived HSPCs.

### Mini-TCR-expressing iPSCs differentiated into CTLs

**Figure 5:**
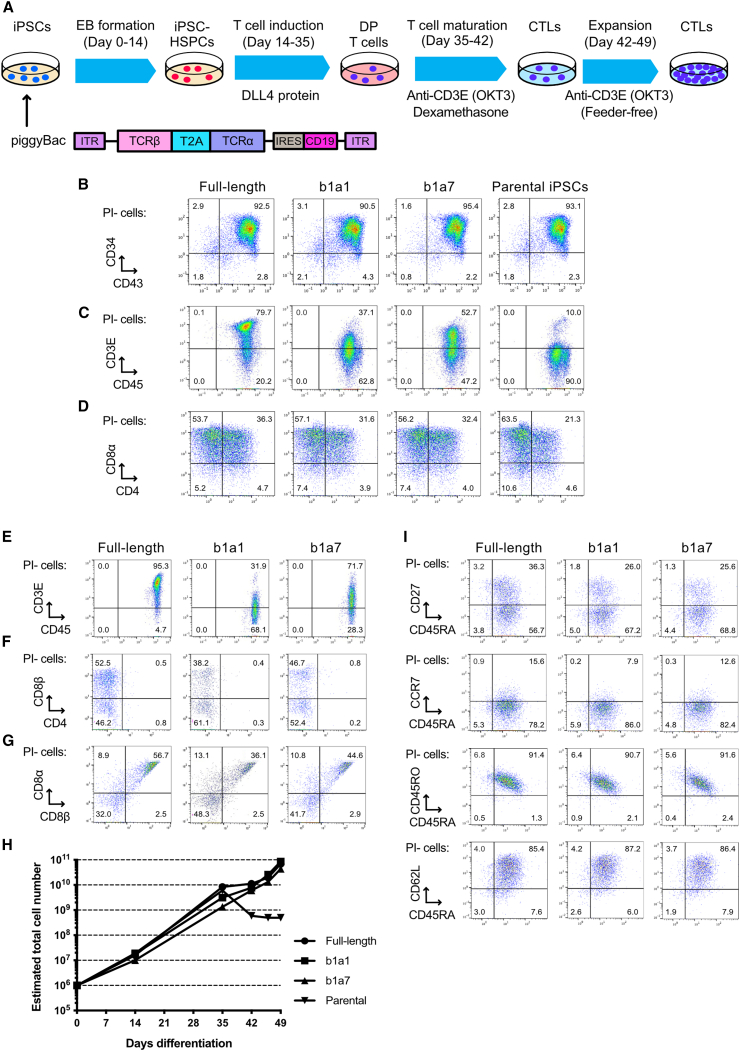
Induction of CTLs from mini-TCR-iPSCs (A) Experimental scheme of T cell differentiation from mini-TCR-iPSCs. TCR genes were expressed with the piggyBac transposon method in iPSCs and the iPSCs were differentiated into CTLs. (B) Representative FACS plots of the CD34 and CD43 expression in iPSC-derived HSPCs. (C) Representative FACS plots of the CD45 and CD3E expression after T cell induction on DLL4 protein. (D) Representative FACS plots of CD4 and CD8α in CD3E + cells after T cell induction. (E–H) Phenotypic analyses of CTLs after T cell maturation using the anti-CD3E antibody and dexamethasone. Representative FACS plots of CD45 and CD3E (E) and CD4 and CD8α (F), and CD8α and CD8β (G) in PI- cells are shown. (H) Estimated cell yields per HSPC. Cell numbers were calculated based on the cell numbers of seeded HSPCs and following proliferation rates (mean of technical duplicate). Representative of three independent experiments is shown. (I) Phenotypic analyses of memory T cell markers in CTLs after T cell maturation. The markers are indicated in the figure. Representative FACS plots of three independent experiments are shown.

We examined whether mini-TCRs could be used in undifferentiated iPSCs as a cell source for T cell generation (Figure 5A). To address this question, a clinical-grade iPSC clone^37^ was transfected with mini-TCRs b1a1 and b1a7 using the piggyBac system by MaxCyte, and transgene-positive cells were isolated via fluorescence-activated cell sorting (FACS) (Figure S5). Concurrently, iPSCs transfected with a full-length Glypican-3 (GPC3)-specific TCR were generated as a positive control. The differentiation of both mini-TCR-iPSCs and full-length TCR-iPSCs into HSPCs was comparable to that of the parental iPSC line and the full-length TCR iPSC (Figure 5B). Furthermore, HSPCs derived from mini-TCR-iPSCs were able to differentiate into CD3+CD4+CD8α+ T cells, similar to full-length TCR-iPSCs, in a clinical-grade T cell induction condition (Figures 5C and 5D).^14^ Furthermore, only progenitor cells derived from mini-TCR-iPSCs and full-length TCR-iPSCs responded to OKT3 stimulation and matured into CD8αβ SP CTLs (Figures 5E–5G).

Cell yields during the differentiation and expansion processes were estimated to reach the magnitude of 1 × 10^10^ cells from an initial 1 × 10^6^ iPSCs (full length, 6.84 × 10^10^; b1a1, 8.58 × 10^10^; b1a7, 4.35 × 10^10^) (Figure 5H). Similar to full-length TCR-iPSC-derived T cells and consistent with our previously reported phenotype of iPSC-derived T cells, both b1a1 and b1a7 iPSC-derived T cells exhibited elevated expression levels of CD45RA, CD62L, and CD45RO, and partial expression levels of CD27 and CCR7 (Figure 5I).^14^^,^^38^ It is worth noting that these CTL-expressing memory T cell markers have been shown to correlate significantly with antitumor efficacy in induced pluripotent stem (iPS)-T cells^14^^,^^38^ as well as in peripheral blood-derived T cells.^39^ Consequently, iPSCs expressing mini-TCR were considered to be a robust cell source for generating CTLs.

### Mini-TCR-iPSC-derived T cells responded to the CD20 antigen when engineered with anti-CD20 CAR

**Figure 6:**
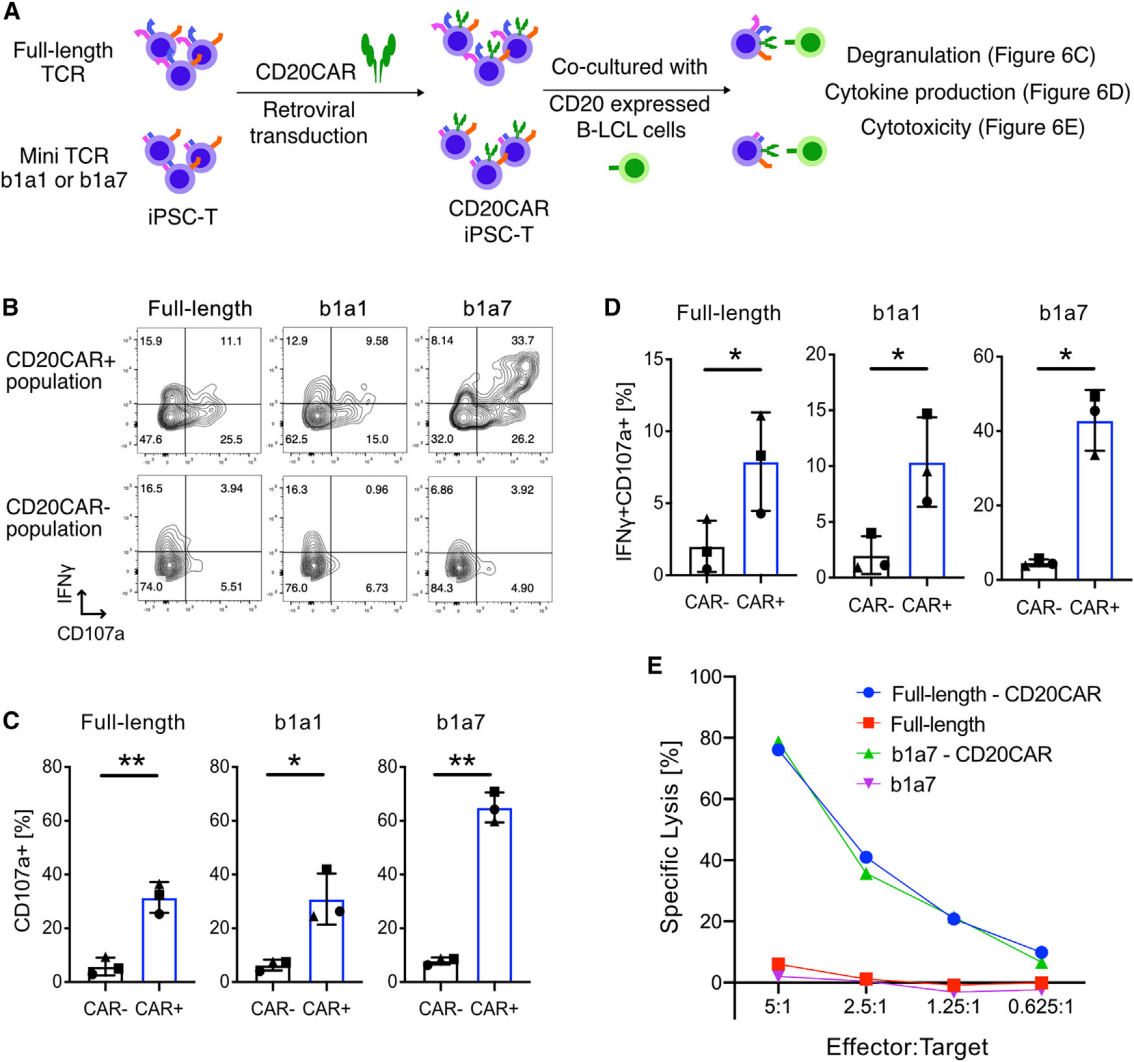
Effector functions of mini-TCR-iPS-CAR T cells (A) Experimental scheme to evaluate effector functions of mini-TCR-iPSC-derived CTLs. The CTLs were transduced with CD20CAR retrovirus, and CD20+ B-LCL cells were employed as the target cells. (B) Representative FACS plots of CD107a and IFNγ in CTLs after co-culture with the target cells. (C) Percentages of CD107a+ cells in CD20CAR− and CD20CAR+ populations. Data are shown as mean ± SD of three independent experiments. (D) Percentages of CD107a+IFNγ+ cells in CD20CAR− and CD20CAR+ populations. Data are shown as mean ± SD of three independent experiments. Paired t tests, ∗p < 0.05, ∗∗p < 0.01, and ∗∗∗p < 0.001. (E) *In vitro* cytotoxicity comparison between mini-TCR (b1a7)-iPS-CAR T cells and full-length TCR-iPS-CAR T cells. The specific lysis after a 3-h incubation with B-LCL cells is shown (mean of technical duplicate). The data represent two independent experiments.

To investigate whether such expanded CTLs could potentially be used for CAR T cell therapy, full-length or mini-TCR-iPSC-T cells were modified by transducing a retroviral vector containing a second-generation anti-CD20 CAR (CD20CAR) (Figures S6A and S6B). The effector function of these CAR-expressing cells was evaluated by co-culturing them with B lymphoblastoid cell line (B-LCL) cells expressing CD20 (Figures 6A and S6C). CD20CAR-expressing mini-TCR-iPSC-T cells exhibited significantly elevated expression of the degranulation marker CD107a compared to CD20CAR-negative populations (Figures 6B and 6C). Intracellular staining revealed that CD107a+ cells produced significantly elevated levels of interferon-gamma (IFNγ), implying that mini-TCR-iPSC-T cells executed their CTLs function in a CAR-dependent manner (Figure 6D). Moreover, after co-culturing with B-LCL cells for 3 h, the CD20CAR-transduced mini-TCR-iPSC-T cells (b1a7) efficiently killed the target cells, with comparable efficacy in comparison to the control CD20CAR-transduced full-length TCR-iPSC-T cells (Figure 6E). These results suggest that mini-TCR-iPSC-T cells expressing CAR exhibit robust effector function, thereby holding promise for potential utilization in CAR T cell therapy.

### iPSC-CAR T cells generated using mini-TCRs had *in vivo* antitumor effects

**Figure 7:**
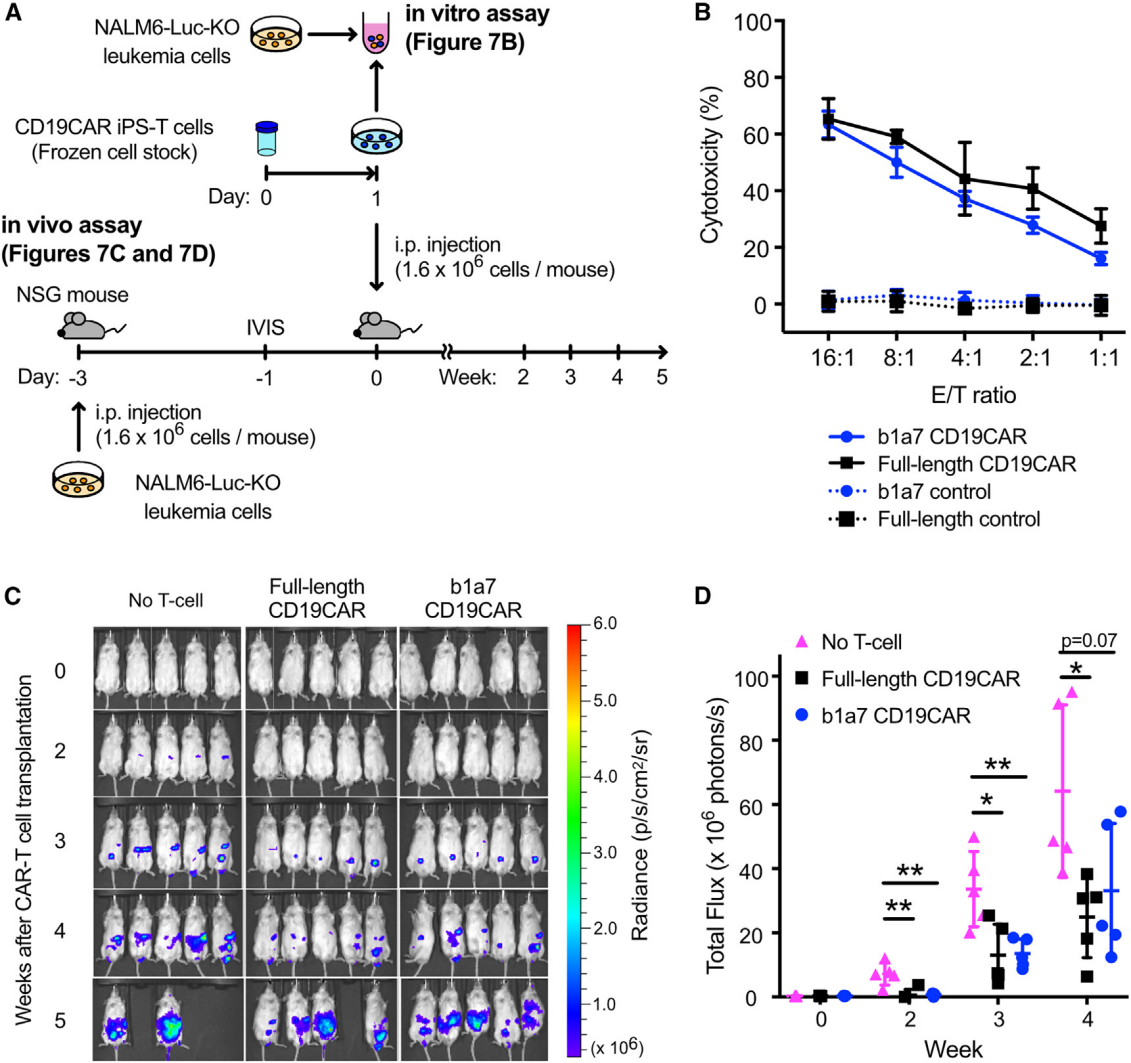
b1a7 CD19CAR T cells exhibit cytotoxic abilities both *in vivo* and *in vitro* (A) Experimental scheme of the *in vitro* and *in vivo* cytotoxicity assays. (B) *In vitro* cytotoxicity of b1a7 CD19CAR and full-length TCR CD19CAR T cells against CD19+ NALM6-Luc-KO leukemia cells. Data are shown as mean ± SD of technical triplicates. (C) IVIS images at the indicated time points. (D) Amounts of NALM6-Luc-KO cells at the indicated time points. The scale bar represents the bioluminescence signal in radiance. Unpaired multiple t tests, ∗p < 0.05, ∗∗p < 0.01, and ∗∗∗p < 0.001.

Finally, we evaluated the antitumor effects of CTLs generated using the mini-TCR b1a7, which showed the highest CD3 recruitment activity and higher cytokine production in response to the CAR antigen (Figure 7A). Another second-generation CAR targeting CD19 (CD19CAR) was transduced into iPSC-derived T cells generated using mini-TCR b1a7 or full-length TCR (hereafter designated as b1a7 CD19CAR T cells or full-length TCR CD19CAR T cells, respectively) (Figure S7A). Surface expression of the CD19CAR protein was confirmed with protein-L staining (Figure S7B). Moreover, luciferase-expressing NALM6 (NALM6-Luc-KO) B-ALL cells were used as the target cells. The *in vitro* cytotoxicity against NALM6-Luc-KO cells of b1a7 CD19CAR T cells was comparable to that of full-length TCR CD19CAR T cells (Figure 7B).

To evaluate the *in vivo* antitumor efficacy, NALM6-Luc-KO cells were injected to NOD.CG-Prkdc scid Il2rg tm1Wjl/SzJ (NSG) mice (day −3) (Figure 7A). The mice were grouped based on the amount of the engrafted tumor (day −1; Figures S7C and S7D). Subsequently, b1a7 CD19CAR or full-length TCR CD19CAR T cells were injected into the mice 3 days after injection of NALM6-Luc-KO cells (day 0). *In vivo* imaging at 3 weeks demonstrated that both b1a7 CD19CAR and full-length TCR CD19CAR T cells significantly suppressed the tumor growth of NALM6-Luc-KO cells compared to untreated control mice (total luminescence at 3 weeks: b1a7 CD19CAR T cells, 135.4 × 10^5^ photons/s; full-length TCR CD19CAR T cells, 130.5 × 10^5^ photons/s; untreated control cells, 336 × 10^5^ photons/s; Figures 7C and 7D). A similar trend was observed at 4 weeks. These data indicated that iPSC-derived T cells generated using mini-TCRs can serve as an allogeneic cell source for CAR T cell therapy.

## Discussion

In this study, we developed mini-TCRs, gene constructs of truncated TCRs, proficient in inducing T cell differentiation from iPSCs. Furthermore, we substantiated an innovative approach for engineering allogeneic CAR T cell therapies. Mini-TCRs successfully triggered T cell differentiation in all four iPSC lines tested. These compelling findings strongly suggest that mini-TCRs have the potential to generate CD8+ T cells from iPSCs as a promising allogeneic cell source platform to enhance CAR T cell therapies. In this section, we elaborately compare the mini-TCRs with full-length TCR and other iPS-T cell-related technologies. Additionally, we explore the future perspectives of iPS-T cells in the context of our findings.

In the screening of mini-TCRs, constant regions of TCRα and TCRβ were cleaved in a stepwise manner, and the combination of these TCRs resulted in a bimodal distribution of CD3 recruitment to the cell surface (Figure 1C). One reason for this could be a change in the interaction between TCRα and TCRβ.^40^ TCRα and TCRβ are held by hydrogen and disulfide bonds in a complex formation that is responsible for recruiting CD3 molecules and transmitting downstream signals.^30^^,^^41^ The deletion of variable regions and extracellular parts of the constant regions can cause structural changes in the binding interfaces that interact with CD3 molecules.^42^^,^^43^ These changes directly affect the stability of the TCR/CD3 complex and the number of CD3E proteins recruited to the cell surface.^44^ The CD3 recruitment activities were markedly decreased when 40–87 amino acids (β4–β9) were removed from the constant region of TCRβ, suggesting the presence of a domain in the constant region of TCRβ that inhibits the binding of CD3 molecules and the TCRα in the absence of N-terminal domains (1–39 amino acids) of the TCRβ constant region. Interestingly, combinations of shorter TCRα and TCRβ constant regions exerted high CD3 recruitment activities, which raises the possibility of interaction modes of these TCR/CD3 complexes that differ from those involving native TCRs.

To date, most studies reporting the induction of T cells from iPSCs have been performed using full-length TCR-overexpressing iPSCs or T cell-derived iPSCs.^14^^,^^18^^,^^19^^,^^20^^,^^36^^,^^45^^,^^46^^,^^47^^,^^48^ Our mini-TCRs achieved an efficient lineage commitment to the T cell lineage in the presence of a DLL4 protein. In T cell development *in vivo*, Notch signaling and pre-TCR signaling coordinate the proliferation of DN cells and their differentiation into DP cells.^41^^,^^49^ Thus, in the differentiation culture condition, mini-TCRs, as well as full-length TCRs, might partially mimic the function of the pre-TCR, which consists of a pre-TCRα and a properly rearranged TCRβ, through the engagement of CD3 molecules on the cell surface.

On the other hand, we observed different expression patterns of CD4, CD8a, and CD8b between full-length TCRs and mini-TCRs when T cells were differentiated from both retroviral-transduced HSPCs and iPSCs transfected with transposon. A DN cell population was formed after the maturation process in mini-TCR-introduced iPSC-T cells, indicating that the developmental cues received from mini-TCRs were weaker than those from full-length TCRs (Figure 4). Moreover, the expression levels of CD3 and CD69 in mini-TCR-expressing Jurkat cells and iPSC-T cells were lower than full-length TCRs, while a variety of transduction efficiency was not observed. Although these phenomena are partially explained by structural differences, as discussed above, as well as the differing reactivity of OKT3 to TCR/CD3 complex, we aim to analyze cellular signaling and/or copy number in greater detail and to optimize the parameters of the cell culture conditions as part of a future study.

Recent reports to improve the efficiency of T cell induction and antitumor efficacy are worth considering for combination with the mini-TCR. Jing et al. reported efficient induction of iPSC-derived CD8ab^+^ T cells by knocking down EZH1 and demonstrated superior *in vivo* antitumor efficacy due to the existence of memory-like T cells that gave rise to effector T cells.^50^ EZH1 is a component of Polycomb Repressive Complex 2 as well as EZH2 and some available EZH1/2 inhibitors, such as lirametostat and valemetostat tosylate, can be added to the medium for feeder-free differentiation.^51^ Stegen et al. reported a T cell induction method from iPSCs involving insertion of the CAR construct to the TRAC locus and achieved high antitumor efficacy.^52^ This approach presents an alternative method for inducing functional CTLs from iPSCs. However, it is essential to note that the associated costs may limit its practicality. Establishing a master cell bank for each CAR construct contributes to the overall expenses involved in this approach. Mini-TCR iPS-T cells offer many specific advantages, such as accepting newly developed CAR constructs including co-expression of memory T cell-related cytokines such as interleukin (IL)-15^14^^,^^53^ and dual-targeting CAR approaches.^54^^,^^55^

Among hundreds of CAR T-related clinical trials, only a few allogeneic peripheral-blood-derived CAR T cell products are under development, such as Allo-501A and Allo-715 by Allogene Therapeutics and CTX110 by CRISPR Therapeutics.^56^^,^^57^ These technologies require complete deletion of the endogenous TCR activities; otherwise, the remaining TCRs might recognize the patients' cells and induce GvHD due to the presence of allogeneic T cells. In FT819, the first iPSC-derived CAR T cells in clinical development, the CAR gene targeting CD19 is inserted into the *TRAC* locus of T cell-derived iPSCs to prevent GvHD.^58^^,^^59^ Our concept offers another safe strategy when starting from allogeneic non-T cell-derived iPSCs because our mini-TCRs, which lack variable regions, would not recognize host cells. Although we have successfully demonstrated the loss of antigen-recognition capacity of the mini-TCR protein, one possible concern at the cellular level is a spontaneous rearrangement of endogenous TCR genes of host iPSCs during differentiation to express alloreactive TCRs. Several studies have reported that expression of endogenous TCR genes was not detectable in differentiated T cells when exogenous TCR genes were overexpressed in iPSCs.^18^^,^^36^^,^^48^ However, it is noteworthy that endogenous TCR genes might still be expressed with a biased repertoire, and the TCR locus rearrangements might be higher in the mini-TCR compared to the full-length TCR, albeit at nearly negligible levels in NGS-based TCR gene expression analysis in this study. In the context of safety assessment for mini-TCR-iPSC-derived CTLs, it is essential to conduct future studies involving mixed leukocyte reactions and incorporate comprehensive manufacturing development processes. When aiming for complete avoidance of the risk of GvHD, disruption of the rearrangement-activating genes *RAG1* or *RAG2* would be worth considering.^36^

Finally, we present a comprehensive summary of the current state of iPS-T cell technology based on published research. Although culture conditions and iPSC lines exhibit variations, iPS-T cells commonly face challenges in maintaining memory T cell phenotypes, such as CCR7 and CD62L, and gene expression profiles during culture. Moreover, they demonstrate weaker cytokine and TCR signaling than clinically established primary CAR T cells. However, recent efforts to enhance iPS-T cell function have shown promising results. For instance, introducing optimized CAR constructs and membrane-bound IL-15 into iPS-T cells has led to comparable antitumor efficacy to primary CAR T cells in mouse tumor models.^14^^,^^15^ Despite the extended duration required for T cell induction from iPSCs, leading to substantial costs, an off-the-shelf approach can mitigate expenses through large-scale manufacturing. It is worth noting that, while numerous engineered receptors, including CARs, have been reported to activate T cells, they typically contain variable domains derived from immunoglobulin or TCR.^45^^,^^46^ Notably, mini-TCRs stand out as the first artificial receptors that lack variable regions while retaining the ability to activate T cells. This unique feature makes iPSCs bearing mini-TCRs an ideal platform for developing allogeneic immunotherapies.

In conclusion, the development of mini-TCRs and the potential use of iPSCs as a platform for allogeneic immunotherapies represent significant advancements in cellular therapies. This approach holds promise for future clinical applications and contributes to the continued progress in cutting-edge cellular therapies.

## Materials and methods

### Sequencing of full-length TCRA and TCRB

Full-length TCRα and TCRβ cDNA were amplified using the SMARTer RACE 5′/3′ Kit (Takara Bio) with T cell-derived iPSCs (TkT3v1-7,^18^ University of Tokyo). Gene-specific primers for the constant regions for the RACE method were as follows: TCRA-5RACE, 5′-CAGCACTGTTGCTCTTGAAGTCCATAGACC-3′; TCRA-3RACE, 5′-ACCGATTTTGATTCTCAAACAAATGTGTCACAAAGTAAGG-3′; TCRB-5RACE, 5′-GGGTTCTGCCAGAAGGTGGCCGAGAC-3′; and TCRB-3RACE, 5′-TGGGAAGGAGGTGCACAGTGGGGTC-3′. Amplicons were sequenced using the standard Sanger sequencing method. The sequences of full-length TCRα and TCRβ cDNA are shown in Tables S3 and S4. The TCRβ was derived from the TRBC2 locus.

### Vector construction

#### Vectors expressing TCRα or TCRβ

The constant regions were amplified by PCR from the full-length-TCRα or TCRβ cDNA samples and a Myc Tag or FLAG Tag was fused to the 3′ end of the constant regions via a GS (glycine serine) linker (sequence: GSGSGS) (designated as TCRA-Myc and TCRB-FLAG, respectively; Figure S1A). The PCR fragment was cloned into the pcDNA3.1(+) mammalian expression vector (Thermo Fisher Scientific) using the In-Fusion HD Cloning Kit (Clontech). The vector was then used as a template for inverse PCR to produce the TCR libraries with the constant region removed in a stepwise process. The sequences of TCRα and TCRβ of the generated TCR library are shown in Tables S5–S8. The sequences of the primers used for inverse PCR are shown in Table S9.

#### CD3 expression vectors

CD3E (NM_000733.3), CD3G (NM_000073.2), CD3D (NM_000732.4), CD3z (NM_198053.2), and EGFP were fused with T2A peptides (CD3E-T2A-CD3G-T2A-CD3D-T2A-CD3Z-T2A-EGFP) and subcloned into the pBApo-EF1α Pur mammalian expression vector (Takara Bio; pEF1α-CD3E-T2A-CD3G-T2A-CD3D-T2A-CD3Z-T2A-EGFP; Figure S1D).

#### TCRβ-T2A-TCRα mammalian expression vectors

To express equal number of TCRα and TCRβ proteins in each cell, the selected pairs of TCRα and TCRβ genes with signal peptides were fused with the GS linker (sequence: SGSG) and the T2A peptide (Figure 2B) and subcloned into the mammalian expression vector comprising EF1 promotor from pBApo-EF1α Pur vector (Takara Bio; Figure S2B), IRES-Kusabiraorange (hKO1, codon optimized for human use, derived from phKO1-S1 purchased from MBL), and BGH polyadenylation signal (designated as pEF1α-IRES-hKO1; Figure S1A).

### Cell culture

293T cells (DSMZ), GP2-293 cells (Takara Bio), and 293FT cells (Invitrogen) were maintained in Dulbecco’s modified Eagle’s medium (DMEM, Nacalai Tesque) supplemented with 10% fetal bovine serum (FBS) and 1% L-glutamine-penicillin-streptomycin solution (PSG; Sigma) at 37°C in a 5% CO_2_ atmosphere. Jurkat cells (DSMZ), B-LCL cells (RIKEN Cell Bank), and NALM6 cells (ATCC) were cultured in RPMI-1640 (Sigma) supplemented with 10% FBS and 1% PSG at 37°C in a 5% CO_2_ atmosphere.

### Flow cytometry

Cells were washed with phosphate-buffered saline (PBS) supplemented with 2% FBS and transferred into 96-well plates (2 × 10^5^ cells/well, 50 μL/well) or 15-mL tubes (1 × 10^6^ cells/tube, 100 μL/tube). Fluorescent-labeled antibodies (2 μL each for 1 × 10^6^ cells; detailed information is described in each section) were diluted with the buffer (total volume was 50 μL/sample) and transferred to the samples. After incubation at 4°C for 30 min, cells were washed with the buffer and resuspend in PBS supplemented with 2% FBS and DAPI (for SONY SH800 cell sorter, FUJIFILM Wako) or propidium iodide (for other machines, Thermo Fisher Scientific) at the volumes of 160 μL/well in 96-well plates or 500 μL/tube for 15-mL tubes. FCS files were analyzed on FlowJo v10 software.

### Establishment of CD3-expressing 293T cells

293T cells (1.1 × 10^5^ cells/well) were seeded into 12-well plates. Next day, the cells were washed with PBS to remove the antibiotics and 1 mL of DMEM supplemented with 10% FBS was added. The CD3 expression vector pEF1α-CD3E-T2A-CD3G-T2A-CD3D-T2A-CD3Z-T2A-EGFP (1 μg) and Lipofectamine 2000 (Thermo Fisher Scientific) were diluted in 50 μL of Opti-MEM (Thermo Fisher Scientific), respectively. After 5 min, the two diluted solutions were combined, incubated at room temperature for 5 min to form DNA-liposome complexes, and transferred into the 293T cells. EGFP+ cells were purified using FACS SH800 (SONY) and maintained to establish a stable transfectant (designated as CD3-OE-293T cells).

### Establishment of TCR-KO Jurkat cells

Genome editing targeting TRAC and TRBC loci was performed to establish a double-KO mutant of Jurkat cells (designated as TCR-KO Jurkat cells) using the Alt-R CRISPR-Cas9 System (Integrated DNA Technologies). TCRαTCRβ double negative cells were purified by FACS on SONY SH800. Disruption of TCRA and TCRB genes was confirmed by single transfection with the full-length TCRα or full-length TCRβ expression vector and subsequent FACS analysis using anti-TCRαβ and anti-CD3E (approximately 99% of the cells were negative for TCRαβ and CD3E). The target genomic sequences were as follows: *TRAC*,^60^ 5′-GAGAATCAAAATCGGTGAAT-3′; *TRBC1/TRBC2*,^61^ 5′-CAAACACAGCGACCTCGGGT-3′. Anti-TCRαβ-APC (clone IP26, BioLegend) and anti-CD3E-PE-Cy7 (clone UCHT1, BioLegend) were used for FACS analysis on a SONY SH800.

### Intracellular staining of TCR proteins

To validate the protein expression from TCRA-Myc or TCRB-FLAG vectors, CD3-OE-293T cells were seeded in 12-well plates at 1.1 × 10^5^ cells/well. The next day, TCRA-Myc or TCRB-FLAG (0.8 μg) and pEF1α-IRES-hKO1 vectors (0.2 μg, as a transfection marker) (total 1.0 μg) were mixed in 50 μL of Opti-MEM and cotransfected into CD3-OE-293T cells as described in the “establishment of CD3-expressing 293T cells” section. Two days after transfection, cells were harvested with trypsin (0.05%)-EDTA phenol red (Gibco) and washed with FACS buffer (PBS [Nacalai Tesque] + 2% FBS [Access Cell Culture] + 1 mM EDTA [Invitrogen]). Then, the cells were fixed with Fixation Buffer (BioLegend) and permeabilized with Perm Wash Buffer (BioLegend). The cells were stained with anti-Myc Tag-Alexa Fluor 647 (clone 9B11, Cell Signaling) or anti-DYKDDDDK (FLAG) Tag-Alexa Fluor 647 (clone L5, BioLegend). The stained cells were suspended in Cell Staining Buffer (BioLegend) and analyzed using BD LSRII (BD Bioscience).

### Detection of CD3E protein on CD3-OE-293T cells

To detect cell surface localization of CD3E protein on CD3-OE-293T cells, the following two combinations of vectors were transfected as described in the “intracellular staining of TCR proteins” section.

(1) TCRA-Myc (0.4 μg), TCRB-FLAG (0.4 μg), and pEF1α-IRES-hKO1 vectors (0.2 μg, as a transfection marker) (total 1.0 μg) (Figures 1 and S1)
(2) TCRβ-T2A-TCRα-pEF1α-IRES-hKO1 vectors (1.0 μg) (Figure 2)

Two to 3 days later, cells were harvested by trypsinization, stained with anti-CD3E-APC (clone UCHT1, BioLegend), and analyzed using a BD LSRII (BD Bioscience).

### Tetramer binding assay

TCRβ-T2A-TCRα-pEF1α-IRES-hKO1 vectors (1.0 μg) including full-length TCRs^35^ were transfected into CD3-OE-293T cells as described in the “intracellular staining of TCR proteins” section. Two to 3 days later, cells were harvested by trypsinization, stained with T-Select HLA-A∗24:02 modified WT1 tetramer-CYTWNQMNL-APC (MBL) and HLA-A∗24:02 control tetramer-AYAAAAAAL-APC (MBL) as described in the “flow cytometry” section and analyzed using a BD LSRII (BD Bioscience).

### Detection of CD3 proteins on TCR-KO Jurkat cells

To detect cell surface expression of CD3E and CD3D proteins on TCR-KO Jurkat cells, TCRβ-T2A-TCRα-pEF1α-IRES-hKO1 vectors (2 μg) were transfected into TCR-KO Jurkat cells (1 × 10^6^ cells/sample, 100 μL/well) by using the 4D-Nucleofector electroporator (Lonza, program: CL-120) and SE Cell Line 4D-Nucleofector X Kit L (Lonza) according to the manufacturer’s instruction.

Two days later, transfected cells were harvested and stained with anti-CD3E-PE-Cy7 (clone UCHT1, BioLegend) and anti-CD3D-APC (clone 7D6, Invitrogen). Biotinylated anti-CD3E antibody OKT3 (BioLegend) was diluted to 200 μg/mL and serial dilution was performed (1:5). Diluted antibody solution (100 μL/well) was transferred to a 96-well plate and mixed with equal amount of TCR-transfected TCR-KO Jurkat cells (1 × 10^5^ cells/sample, 100 μL/well). After incubation (4°C, 30 min), cells were washed twice and stained with Alexa 647-labeled streptavidin (BioLegend, diluted in 1:400, 50 μL/sample). Samples were prepared as described in the “flow cytometry” section and analyzed using a BD LSRII (BD Bioscience).

### CD3 stimulation of TCR-KO Jurkat cells

Transfection into TCR-KO Jurkat cells was performed as described in the “detection of CD3 proteins on TCR-KO Jurkat cells” section and incubated overnight (day 0). Ultra-LEAF purified anti-human CD3 (clone OKT3, BioLegend) was plated into 96-well plates (10 μg/mL in PBS, 100 μL/well) and incubated overnight (day 0). After washing the CD3-coated plate with PBS, transfected TCR-KO Jurkat cells or parental Jurkat cells were seeded at the cell density of 1–2 × 10^5^ cells/well and incubated overnight (day 1). Jurkat cells were stained with anti-CD69-Alexa Fluor 647 (clone FN50, BioLegend) and analyzed using a BD LSRII (BD Bioscience).

### Retroviral production

Full-length and selected pairs of TCRβ and TCRα (b1a1 and b1a7) described in the section “TCRβ-T2A-TCRα mammalian expression vectors” were transferred into pMYs-IRES-GFP retroviral vector (pMYs-IG, Cell Biolabs). The CD19CAR sequence was obtained from an application for a US patent (US2014-0271635). The synthesized CD19CAR cDNA was subcloned into pMYs-IG. The CD20CAR cDNA^16^^,^^62^ was subcloned into the pGCDNsam vector.^63^

For viral production, GP2-293 cells (Takara Bio) were seeded onto poly-L-lysine 5× (Sigma-Aldrich)-coated 10-cm dishes (5 × 10^6^ cells/dish) and incubated overnight. pMYs-IG vectors (2 μg) and VSV-G vector (Takara Bio, 2 μg) were diluted in Opti-MEM (200 μL) and combined with Lipofectamine 2000 (Thermo Fisher Scientific; 12 μL were diluted in Opti-MEM 200 μL). After 5 min of incubation at room temperature, they were transfected into the GP2-293 cells and incubated for 2 days. The harvested supernatant (10 mL/dish) was filtered and mixed with a Retro-X Concentrator (Takara Bio, 3.3 mL) and incubated overnight at 4°C. After centrifugation (at 1,500 × *g*, 45 min, 4°C), the pellet was resuspended in alpha-MEM (300 μL). The titers were determined by infection with the serial diluted viruses to Jurkat cells and FACS analyses. Aliquots were stored in a −80°C deep freezer until further use.

### Lentiviral production and establishment of NALM6-Luc-KO cells

The GFP gene in CMV-Luciferase-EF1a-copGFP-T2A-Puro Lentivector (System Biosciences) was replaced with hKO1 amplified from the phKO1-S1 vector (MBL). Lentivirus was produced as previously described using 293FT cells.^35^ The concentrated lentivirus was titrated with Jurkat cells and infected to NALM6 cells (MOI = 6) by the spin-infection method in 24-well plates (32°C, 2000 × *g*, 2 h). hKO1+ NALM6 cells were sorted on BD FACSAria II and expanded.

### T cell induction from iPSCs

#### Maintenance of feeder-free iPSCs

Myeloid-derived iPSCs (Ff-WJs513, Ff-WJs524, Ff-WJs527, and FfI01s04; all clones were established from cord-blood cells in CiRA, Kyoto University^64^^,^^65^) and T cell-derived iPSCs (TkT3V1-7, provided by the University of Tokyo) were maintained in StemFit AK03N (Ajinomoto) on iMatrix-511 (Matrixome) as previously reported.^37^

#### Establishment of TCR-expressing iPSCs

Full-length GPC3-specific TCRs (provided by Shinobi Therapeutics) and selected pairs of TCRβ and TCRα (b1a1 and b1a7) described in section “TCRβ-T2A-TCRα mammalian expression vectors” were transferred into piggyBac transfer vector (kindly provided by Dr. Yozo Nakazawa, Shinshu University). iPSCs (FfI01s04, 4 × 10^6^ cells/sample) were washed with MaxCyte buffer (HyClone, EPB1) three times and resuspended in MaxCyte buffer (4 × 10^6^ cells per 100 μL) in an OC-100 cuvette. Then, the transfer and transposase vectors (16 μg each) were electroporated into the cells using MaxCyte STX (MaxCyte) in the Optimization 8 condition. Following the electroporation, the cells were immediately placed in culture medium and incubated at 37°C and 5% CO_2_ for 30 min. Then, the cells were transferred to iMatrix-511 coated six-well plates with StemFit AK03. After 5–7 days, CD19-positive cells were sorted by FACS (Figure S5).

#### Generation of HSPCs from iPSCs

HSPCs were induced by using the embryoid body (EB) method as previously reported.^14^ Briefly, iPSCs dissociated into single cells using 0.5× TrypLE Select (Thermo Fisher Scientific) and 0.75 mM EDTA were resuspended in StemFit AK02N supplemented with 10 μM Y-27632 (Nacalai Tesque) and 10 μM CHIR99021 (Tocris Bioscience) and cultured in six-well ultra-low attachment plates (Corning) for 24 h at a concentration of 0.2–0.3 × 10^6^ cells/well in a 5% O_2_ incubator (day 0). The next day, the cells were harvested and resuspended in 2 mL of StemPro-34 (Thermo Fisher Scientific) supplemented with 0.2× PSG, 1× GlutaMAX (Thermo Fisher Scientific), 50 μg/mL ascorbic acid-2-phosphate (PAA, FUJIFILM Wako), 45 mM monothioglycerol (FUJIFILM Wako), and 1× insulin-transferrin-selenium solution (Thermo Fisher Scientific) (hereafter referred to as EB basal medium), 50 ng/mL recombinant human (rh) BMP-4 (Miltenyi Biotec), 50 ng/mL rhVEGF (FUJIFILM Wako), and 50 ng/mL bFGF (FUJIFILM Wako) per well (day 1, 5% O_2_, 5% CO_2_). After 24 h, 6 μM SB431542 (FUJIFILM Wako) was added (day 2, 5% O_2_, 5% CO_2_). After 2 days, the differentiating EBs were collected, washed, and resuspended in 2 mL of EB basal medium supplemented with 50 ng/mL rhVEGF, 50 ng/mL rhbFGF, and 50 ng/mL rhSCF (R&D Systems) per well and cultured for 2 days (day 4, 5% O_2_, 5% CO_2_). After 2 days, the EBs were cultured in 2 mL of EB basal medium supplemented with 50 ng/mL rhVEGF, 50 ng/mL rhbFGF, 50 ng/mL rhSCF (R&D Systems), 30 ng/mL rhTPO (FUJIFILM Wako), and 10 ng/mL rhFLT3L (FUJIFILM Wako) (day 6, 5% CO_2_). The medium was changed every 2–3 days until day 13. Harvested cells were resuspended in TC-Protector cell freezing medium (Iwai) and stored in a −150°C deep freezer.

#### Transduction of TCR genes and T cell induction

HSPCs were differentiated into T cells as previously described.^14^ Briefly, thawed EB-derived HSPCs (from Ff-WJs513, Ff-WJs524, and Ff-WJs527 cells) were incubated in EB medium supplemented with 50 ng/mL VEGF-165A, 50 ng/mL rhbFGF, 50 ng/mL rhSCF, 50 ng/mL rhTPO, and 10 ng/mL rhFLT3L (day −2). The next day, cells were seeded into 48-well plates coated with RetroNectin (Takara Bio, 100 μg/mL) at a cell density of 2–4 × 10^4^ cells/well. Titrated retrovirus was added to the cell suspension (day −1, MOI = 20). The next day, cells were seeded into plates coated with RetroNectin (5 μg/mL) and DLL4 protein (5 μg/mL) and incubated in alpha-MEM supplemented with 50 ng/mL rhSCF, 50 ng/mL rhIL-7 (FUJIFILM Wako), 50 ng/mL rhFLT3L, 100 ng/mL rhTPO, 30 nM rhSDF-1α (FUJIFILM Wako), 15 μM SB203580 (FUJIFILM Wako), 50 μg/mL PAA, 15% FBS, and 1% PSG (day 0). The medium was changed every 2–3 days, and cells were reseeded into fresh RetroNectin- and DLL4-coated 48-well plates every 7 days until day 21. Anti-CD3-BV510 (BioLegend) and anti-CD45-APC-Cy7 (BioLegend) were used for FACS analysis.

#### Maturation of T cell progenitors to CD8α+CD8β+ CTLs

T cells induced on DLL4 proteins were seeded into 48-well plates at a density of 1–10 × 10^4^ cells/well and stimulated with OKT3 anti-CD3 antibody (eBioscience, 500 ng/mL) in alpha-MEM supplemented with 15% FBS, 50 μg/mL PAA, 1% PSG, 10 ng/mL rhIL-7, and 10 nM dexamethasone (DEXART, Fuji Pharma) for 3 days (days 21–24).^14^^,^^36^ OKT3 and dexamethasone were removed by changing the medium (alpha-MEM supplemented with 15% FBS, 50 μg/mL PAA, 1% PSG, and 10 ng/mL rhIL-7), and cells were incubated for another 7 days (days 24–31). Anti-CD8β-PE-Cy7, anti-CD8α-APC, anti-CD45-APC-Cy7, anti-CD4-BV421, and anti-CD3-BV510 antibodies were used for FACS analysis.

#### T cell expansion

CTLs were co-cultured with irradiated PBMCs (40 Gy) as feeder cells in alpha-MEM supplemented with 10% FBS, 1% PSG, IL-7 (5 ng/mL), IL-15 (5 ng/mL), pan-caspase inhibitor Z-VAD-FMK (10 μM, R&D Systems), PAA (50 μg/mL), ITS-G, and phytohemagglutinin (PHA; 2 μg/mL, FUJIFILM Wako) for 14 days. Feeder-free expansion was performed based on the previous report.^14^ The medium was replenished every 2–3 days, and cell passaging was performed every 2–6 days.

### TCR gene expression analysis

The NGS to evaluate expression of endogenous and transduced TCR genes was performed as previously described.^36^ Total RNA was isolated from CTLs after expansion (see the section “T cell expansion”) using RNeasy micro kit (QIAGEN) following the manufacturer’s instructions. After reverse transcription, the cDNA was amplified by using adopter primer at the 5′ and the reverse primers to constant domains of TCRA and TCRB. After the sequencing of the amplified CDR3 regions from both TCRA and TCRB genes on a next-generation sequencer, entire sequenced reads (number of reads are depicted as “Total reads” in Tables S1 and S2) were analyzed using the repertoire analysis software Repertoire Genesis to assign TRV and TRJ alleles to queries and then generate CDR3 sequences and aggregate their combination patterns ("Total assigned reads" in Tables S1 and S2). In-frame sequences for which the CDR3 region can be translated to amino acid sequences were analyzed (Figures 4J and S4B; "Total in-frame reads" in Tables S1 and S2).

### Retroviral transduction into iPSC-derived T cells

Anti-CD19 CAR retrovirus was transduced into iPS-T cells. A 96-well plate was coated with RetroNectin (Takara Bio) for 2 h at room temperature or overnight at 4°C. Two days after CD3/CD28 stimulation, iPS-T cells were transduced with concentrated retroviral supernatants by centrifugation on a RetroNectin-coated plate (MOI = 20). Cells were cultured for 11 days from transduction (total 13 days). Transduction was confirmed with flow cytometry with protein-L (GenScript) and streptavadin-APC (BioLegend). The cryopreserved cell stocks were prepared as outlined in “generation of HSPCs from iPSCs” section (Figure S7A).

### CD107a and cytokine detection

For detection of CD107a and IFNγ expression of iPSC-T cells, T cells were co-cultured with B-LCLs cells at a 1:1 ratio in a 96-well plate for 5 h in 100 μL of RPMI-1640 supplemented with glutamine, 1× Monensin (BioLegend), 10% FBS, and anti-CD107a-APC antibody (BioLegend, Clone H4A3, 1 μL). Then the cell surface was stained with anti-CD20-PE-Cy7 (BD Pharmingen) and anti-EGFR-PE (BioLegend). Samples were washed and fixed with Fixation Buffer (BioLegend) for 20 min. Samples were washed twice with Permeabilization Wash Buffer (BioLegend) and were stained with IFNγ-APC-Cy7 (BioLegend) for 20 min. The washed and resuspended cells were analyzed using BD FACSAria II.

### *In vitro* cytotoxicity assay

A nonradioactive cellular cytotoxicity assay kit (Techno-Suzuta, Nagasaki, Japan) was used for CD20CAR T cells. Briefly, target B-LCLs were pulsed with BM-HT reagent at 37°C for 15 min, washed three times, and seeded into 96-well plates at a density of 5,000 cells/well. Effector cells were added to the wells at effector-to-target (E:T) ratios ranging from 5:1 to 0.625:1 and were co-cultured for 3 h with α-MEM supplemented with 15% FBS and 1% PSG at 37°C with 5% CO_2_. Then, 40 μL of the co-culture supernatant was collected and mixed with 160 μL of Eu solution, and time-resolved fluorescence was measured using a NIVO plate reader (PerkinElmer, Waltham, MA).

*In vitro* cytotoxicity of the CD19CAR T cells were examined using the standard 51Cr release method. Briefly, target NALM6-Luc-KO cells were loaded with 1.85 MBq 51Cr for 1 h in α-MEM supplemented with 15% FBS and 1% PSG. Target cells (5,000 cells/well) were co-cultured with effector iPSC-derived CAR T cells for 5 h in the medium with various effector-to-target (E:T) ratios ranging from 16:1 to 1:1. Supernatants were harvested and the released 51Cr was measured using a beta counter (PerkinElmer).

### Mouse leukemia model

Male NSG mice (11 weeks old; Charles River Laboratories Japan) were housed under controlled humidity and light/dark cycle in a specific pathogen-free facility. All animal experiments were performed in accordance with the Animal Review Board of Kyoto University. A total of 1 × 10^6^ NALM6-Luc-KO cells were intraperitoneally (i.p.) injected to establish a mouse leukemia model (day −3). From day −1, the tumor burden was monitored weekly by *in vivo* bioluminescence imaging using IVIS SPECTRUM and Living Image 4.7.3 software (PerkinElmer). CAR T cells (1.6 × 10^6^ cells/mouse) were i.p. injected into mice grouped according to the luminescence on day 0. From day −3, body weight was monitored daily, and 20% weight loss compared to that on day −3 was used as the humane endpoint for sacrifice.

## Data and code availability

The sequences identified and designed during this study are available from supplementary tables or from the corresponding author upon reasonable request.

## Supplemental information

Document S1. Figures S1–S7 and Tables S1–S9

Document S2. Article plus supplemental information
