# Supplementary material for: Mini-TCRs: Truncated T cell receptors to generate T cells from induced pluripotent stem cells
Source: Mol Ther Methods Clin Dev. 2023 Sep 16;31:101109. doi: 10.1016/j.omtm.2023.101109 (PMC10562677; doi:10.1016/j.omtm.2023.101109)
Supplement: Document S1. Figures S1–S7 and Tables S1–S9 [file mmc1.pdf]

OMTM, Volume *31*

Supplemental information

Mini-TCRs: Truncated T cell receptors to generate T cells from induced pluripotent stem cells

Shin-ichiro Takayanagi, Bo Wang, Saki Hasegawa, Satoshi Nishikawa, Ken Fukumoto, Kohei Nakano, Sayaka Chuganji, Yuya Kato, Sanae Kamibayashi, Atsutaka Minagawa, Atsushi Kunisato, Hajime Nozawa, and Shin Kaneko

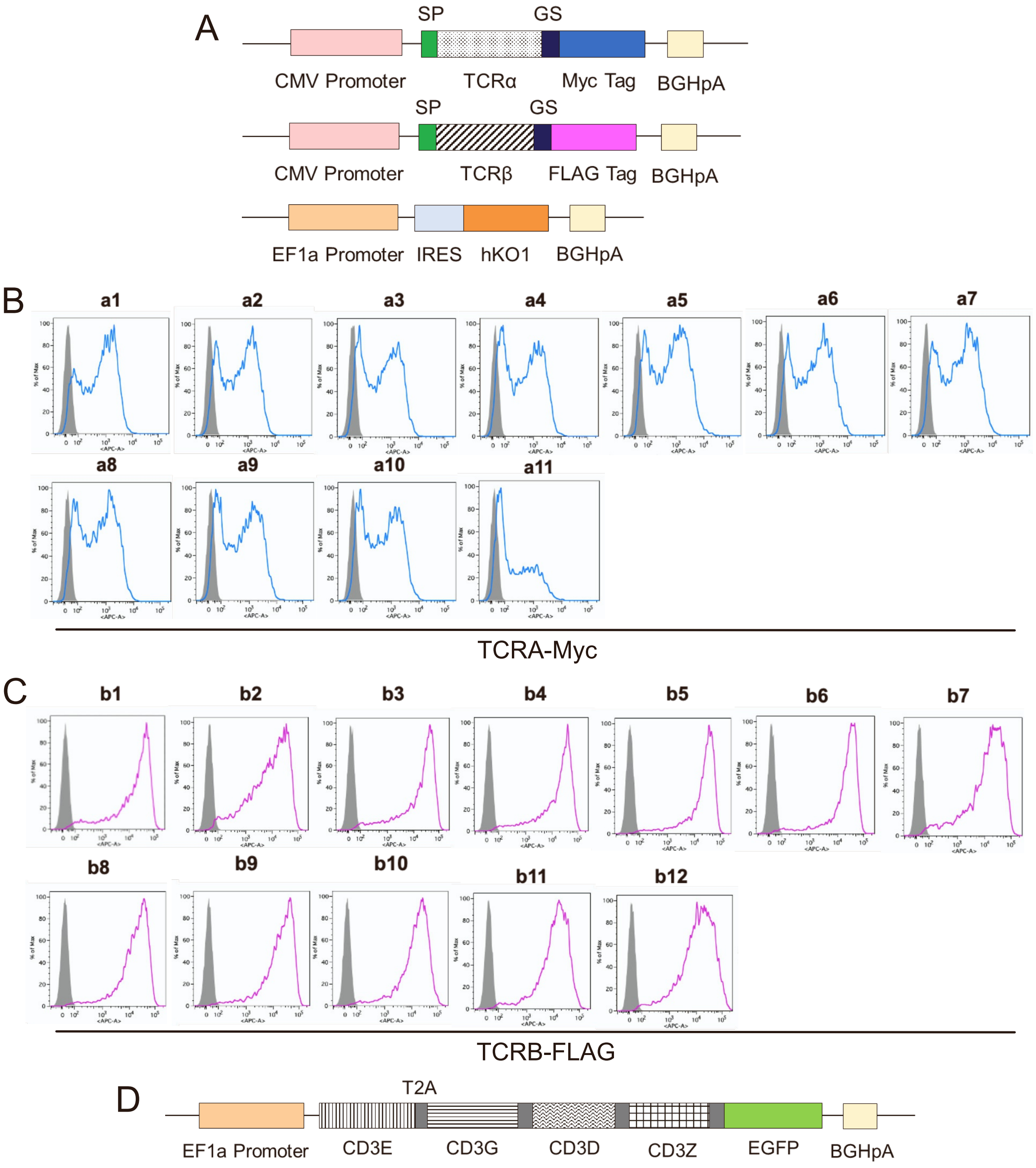

Figure S1, Related to Figure 1. Details of the experimental materials and verification of TCR protein expression.

(A) Structures of the TCRA-Myc (top), TCRB-FLAG (middle), and hKO1 (bottom) expression vectors used in the screening of TCR constructs. hKO1 fluorescent protein was used as a transfection marker.

(B) (C) Intracellular staining to evaluate expression of TCRA-Myc (B) and TCRB-FLAG proteins (C). hKO1+GFP+ cells are shown.

(D) The CD3 protein expression vector used in the CD3-OE-293T cells.

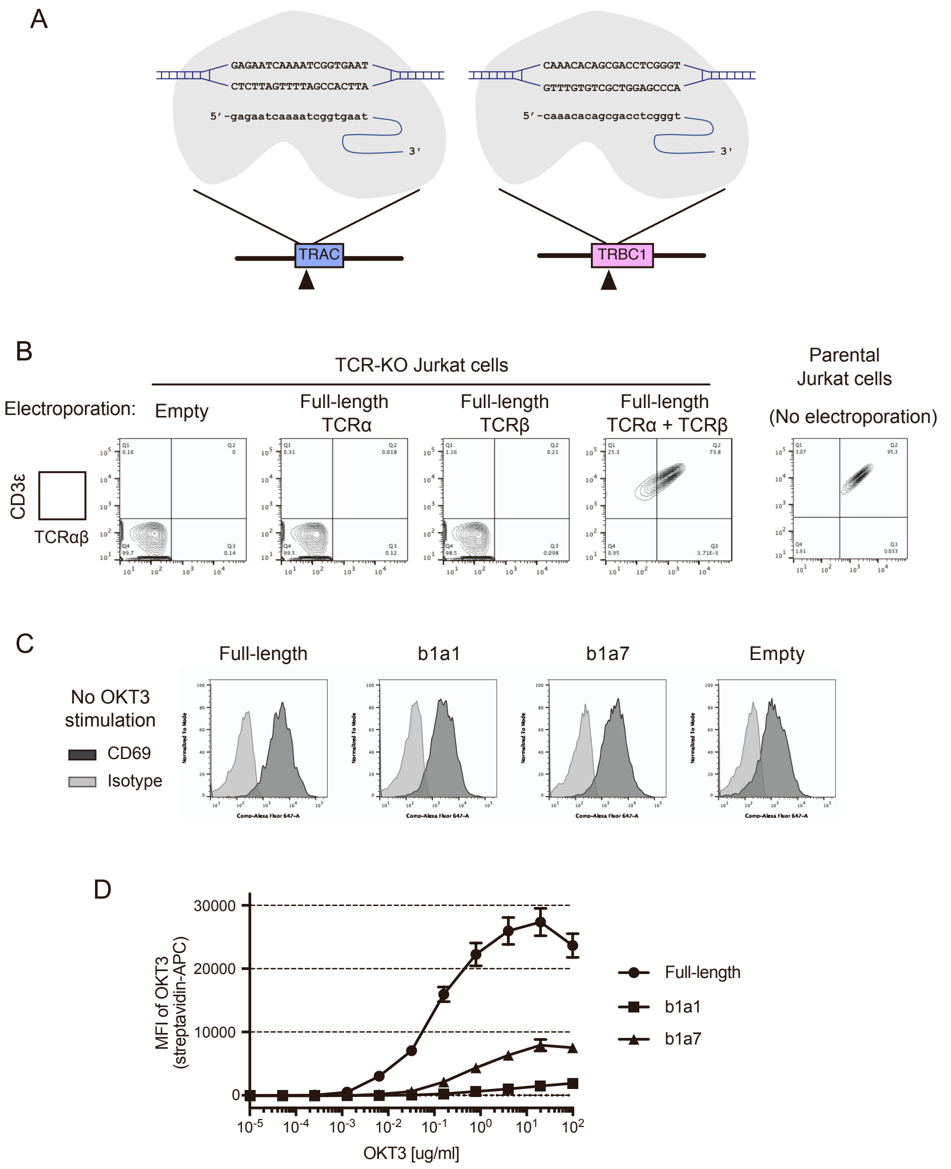

Figure S2, Related to Figure 3. Details of TCR-KO Jurkat cells

(A) gRNA sequences targeting TRAC (left) and TRBC1 (right) loci using the CRISPR/Cas9 method.

(B) FACS plots of TCR-KO and parental Jurkat cells after electroporation of full-length TCRα and/or TCRβ or empty control vector.

(C) Representative FACS plots of CD69 expression on non-stimulated TCR-KO Jurkat cells electroporated with each vector, related to Figure 3E-F.

(D) Mean fluorescence intensity (MFI) of OKT3 to TCR-KO Jurkat cells electroporated with each vector before normalization, related to Figure 3G.

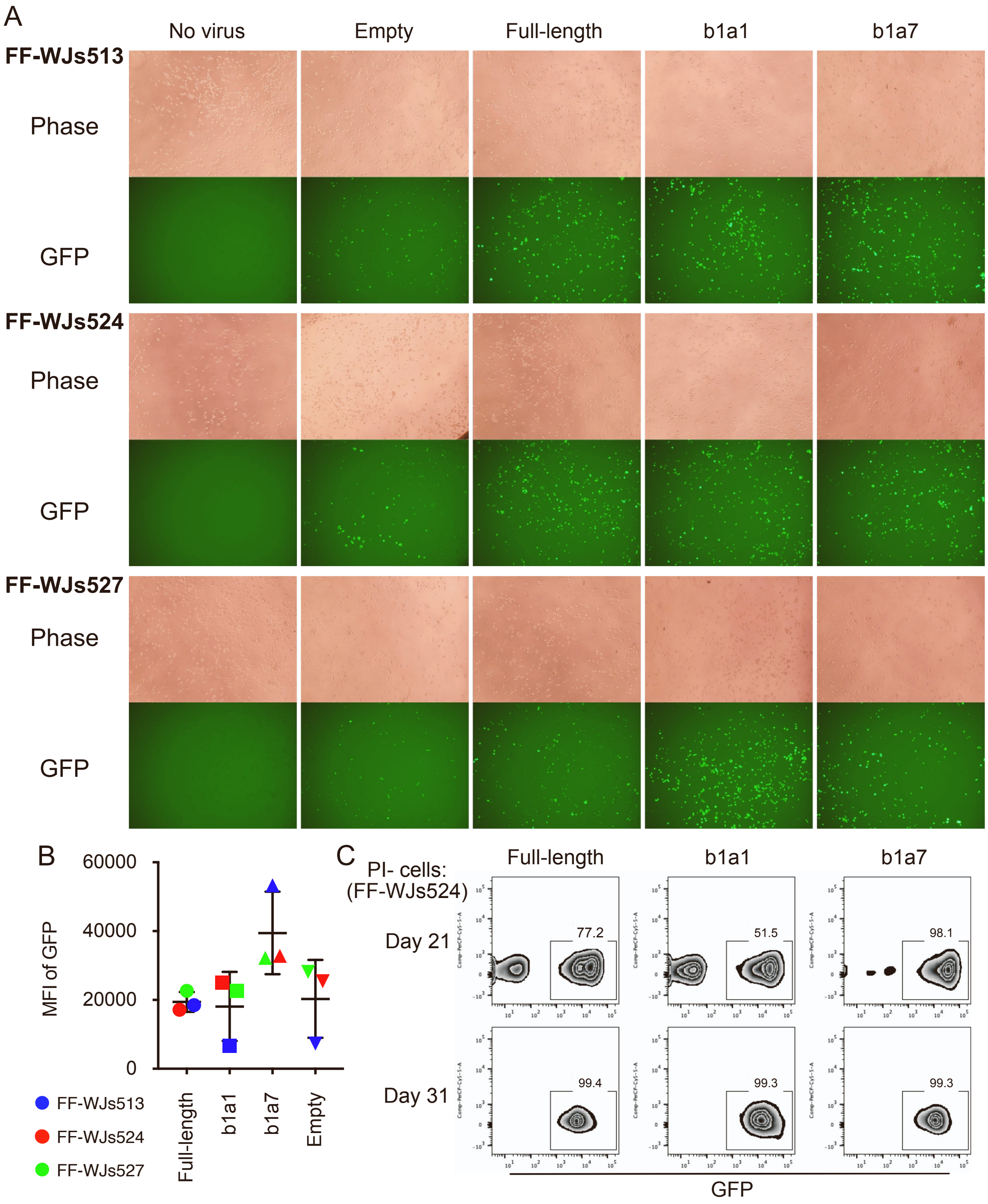

Figure S3, Related to Figure 4.

(A) Fluorescent microscopy images of iPSC-derived HSPCs six days after retroviral transduction.

(B) MFI of GFP in GFP+ cells after T cell induction on DLL4 protein (Day 21).

(C) Flow cytometry before (Day 21) and after (Day 31) T cell maturation with anti-CD3E antibody OKT3 and dexamethasone.

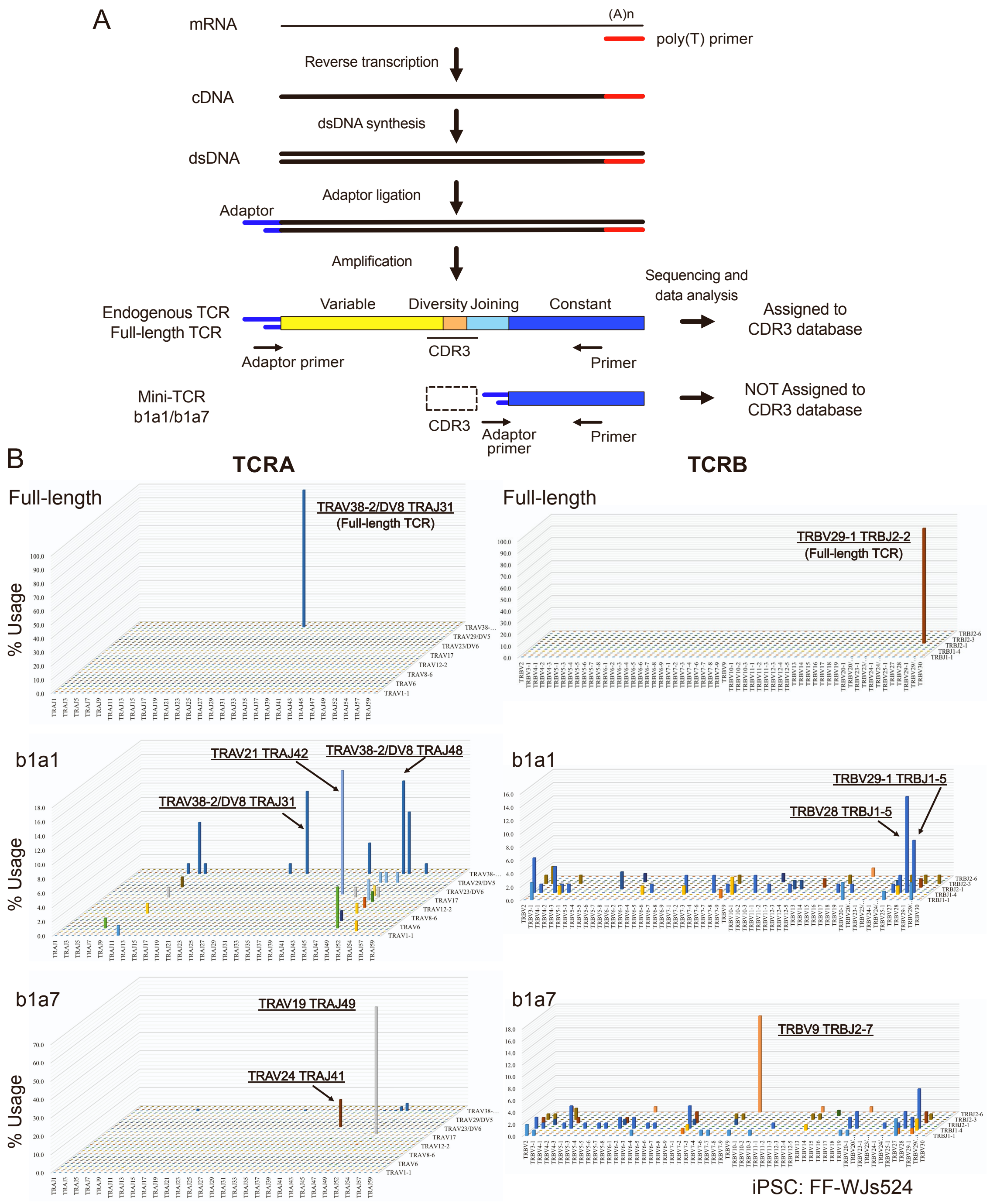

Figure S4, Related to Figure 4. TCR gene expression analysis by Next-generation sequencing

(A) Schematic illustration of NGS-based analysis of TCR gene expression.

(B) TRAVJ (left) and TRBVJ (right) repertoire 3D graph. Data from FF-WJs524 iPSCs are shown as representative of two iPSC-derived CTLs.

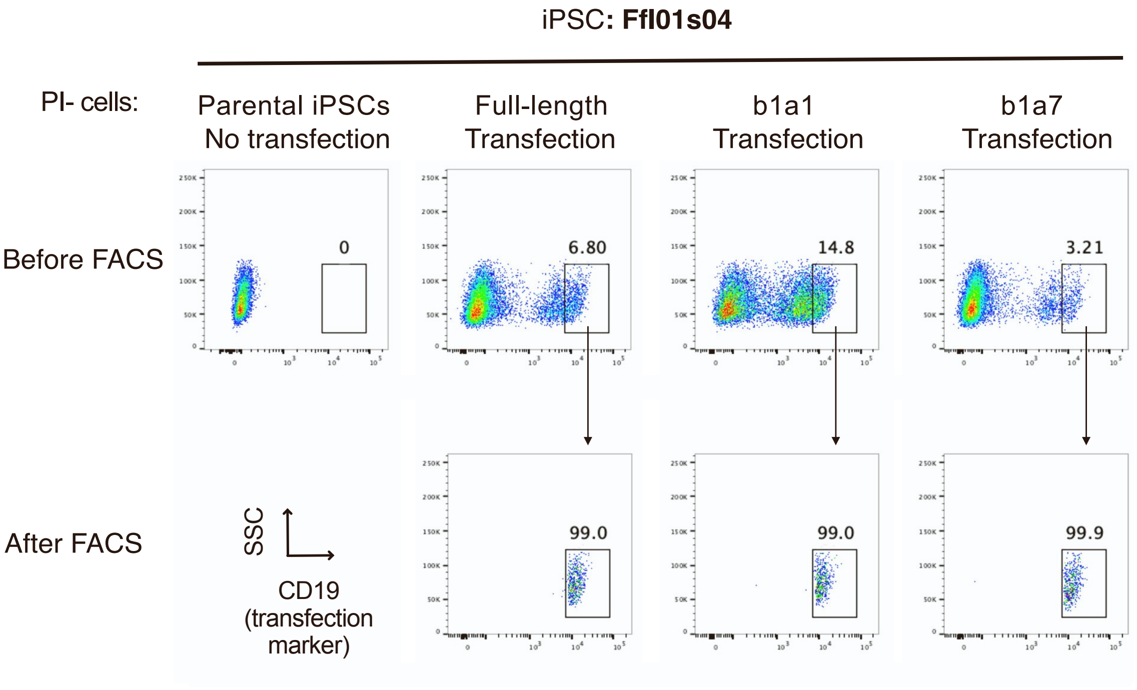

Figure S5, Related to Figure 5. Establishment of Mini-TCR-iPSCs.

Full-length or Mini-TCR b1a1 or b1a7 were inserted into the transposon vector and transfected into iPSCs (clone Ffl01s04). Flow cytometry data before FACS (upper panels) and reanalysis data after FACS to confirm the purity (lower panels). CD19 was used as the transfection marker.

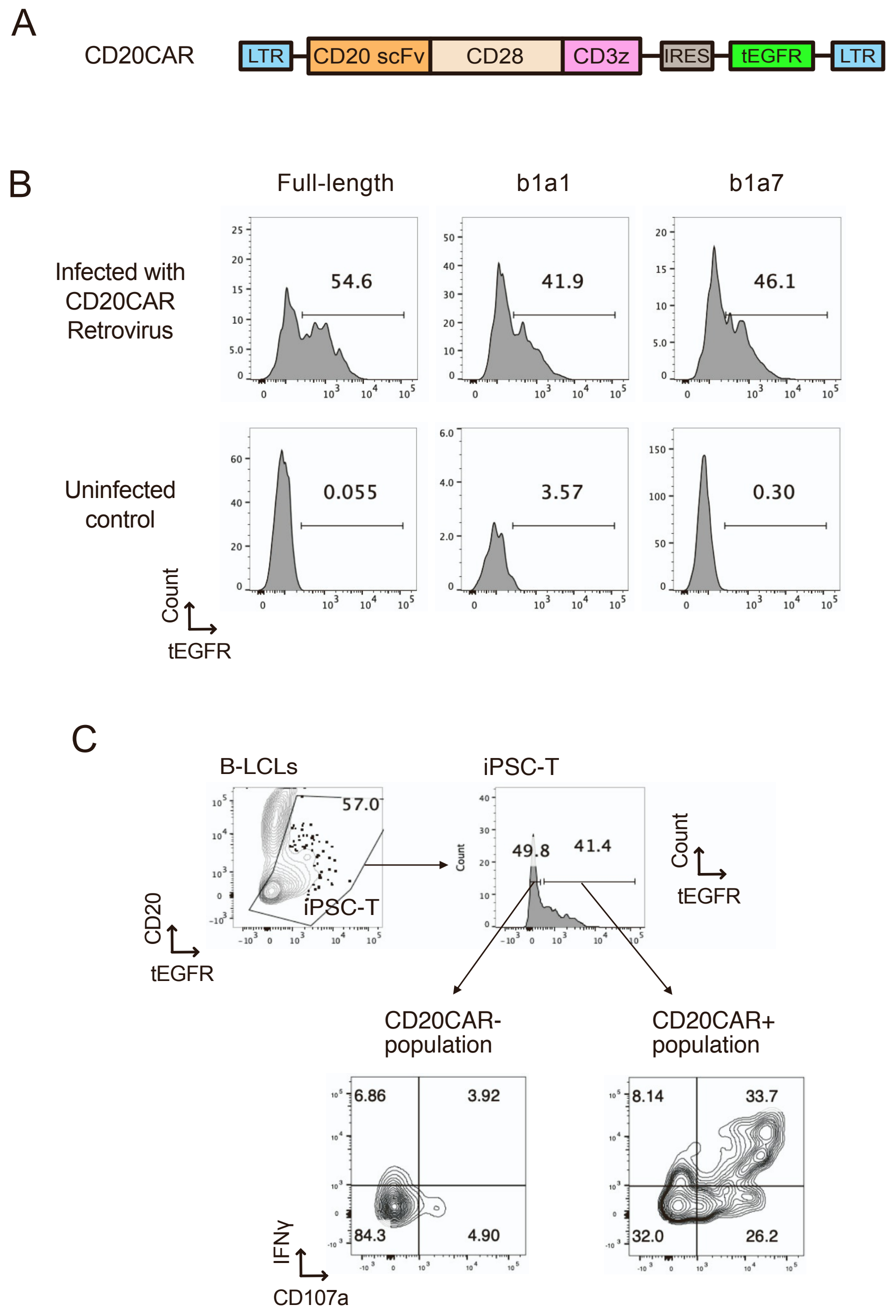

Figure S6, Related to Figure 6. Details of the cytokine production and in vitro cytotoxicity assay.

(A) The structures of the CD20CAR expression vector.

(B) Representative plots of CD20CAR expressing iPSC-derived T cells.

(C) The gating strategy for the cytokine production assay of CD20CAR expressing iPSC-derived CTLs.

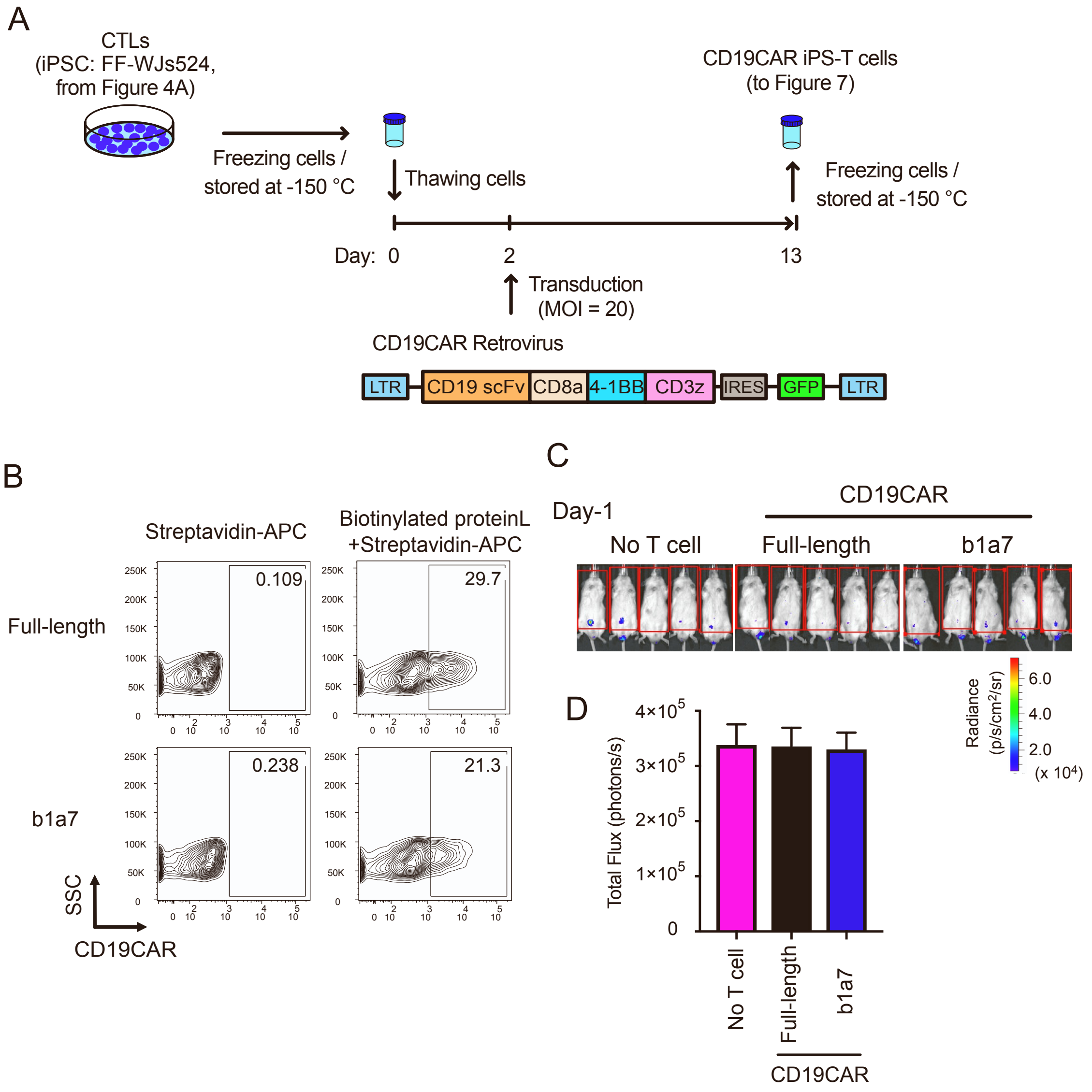

Figure S7, Related to Figure 7. Experimental materials.

(A) Preparation of CD19CAR iPS-T cells and the structure of the CD19CAR vector.

(B) Expression of the CD19CAR protein in iPSC-T cells induced with full-length-TCR or Mini-TCR b1a7. The CD19CAR protein was detected with protein-L that binds to the anti-CD19 single-chain variable fragment (scFv).

(C) *In vivo* bioluminescence images to monitor the amounts of NALM6-Luc-KO cells on Day −1.

(D) Total flux (photons/s) on Day -1. Data are shown as mean ± S.D. of 5 biological replicates.

Table S1. The details of sequencing read in the NGS-based TCRA gene expression analysis

Numbers of sequenced reads were compared with the previous report.[35]

In-frame: Number of reads for which the CDR3 region can be translated to amino acid sequences

Assigned: Number of reads judged as TCRA genes in the Repertoire Genesis software

Total: Number of total reads in NGS

%Read = (Number of reads) / (In-frame read number) x 100

[36]: Data from our earlier report (Minagawa, A. et al. (2018). Enhancing T Cell Receptor Stability in Rejuvenated iPSC-Derived T Cells Improves Their Use in Cancer Immunotherapy. Cell Stem Cell 23, 850–858.e4.)

Sample	Rank	TRAV	TRAJ	CDR3	Reads	%Read
FF-WJs524-Full-length	1	TRAV38-2/DV8	TRAJ31	CAYWSNNNARLMF	107486	96.51
	2	TRAV38-2/DV8	TRAJ31	CAYWSNNNDRLMF	144	0.13
	3	TRAV38-2/DV8	TRAJ31	CAYWSNSNARLMF	107	0.10
	4	TRAV38-2/DV8	TRAJ31	CAYWSNNNVRLMF	106	0.10
	5	TRAV38-2/DV8	TRAJ31	CAYWGNNNARLMF	99	0.09
	In-frame				111368	35.03
	Assigned				113385	35.66
	Total				317963	
FF-WJs524-b1a1	1	TRAV21	TRAJ42	CAVILWRSQGNLIF	12	17.39
	2	TRAV38-2/DV8	TRAJ48	CVSNFGNEKLTF	9	13.04
	3	TRAV38-2/DV8	TRAJ31	CAYWSNNNARLMF	4	5.80
	4	TRAV38-2/DV8	TRAJ12	CGMDSSYKLIF	4	5.80
	5	TRAV38-2/DV8	TRAJ49	CLSQWNTGNQFYF	3	4.35
	In-frame				69	0.02
	Assigned				649	0.19
	Total				338072	
FF-WJs524-b1a7	1	TRAV19	TRAJ49	CALSEASNTGNQFYF	471	67.38
	2	TRAV24	TRAJ41	CAASNSNSGYALNF	98	14.02
	3	TRAV38-2/DV8	TRAJ48	CVSNFGNEKLTF	11	1.57
	4	TRAV38-2/DV8	TRAJ12	CGMDSSYKLIF	5	0.72

	5	TRAV38-2/DV8	TRAJ49	CLSQWNTGNQFYF	4	0.57
	In-frame				699	0.23
	Assigned				1761	0.59
	Total				297521	
FF-WJs527-Full-length	1	TRAV38-2/DV8	TRAJ31	CAYWSNNNARLMF	124717	96.43
	2	TRAV3	TRAJ17	CAVRDSRAAGNKLTF	931	0.72
	3	TRAV38-2/DV8	TRAJ31	CAYWSNNNAGLMF	145	0.11
	4	TRAV38-2/DV8	TRAJ31	CAYWSNNNDRLMF	122	0.09
	5	TRAV38-2/DV8	TRAJ31	CAYWSNSNARLMF	120	0.09
	In-frame				129340	
	Assigned				131259	
	Total				393623	
FF-WJs527-b1a1	1	TRAV38-2/DV8	TRAJ48	CVSNFGNEKLTF	5	16.67
	2	TRAV38-2/DV8	TRAJ31	CAYWSNNNARLMF	3	10.00
	3	TRAV38-2/DV8	TRAJ49	CLSQWNTGNQFYF	2	6.67
	4	TRAV38-2/DV8	TRAJ47	CVEYGNKLVF	2	6.67
	5	TRAV8-4	TRAJ48	CVSNFGNEKLTF	1	3.33
	In-frame				30	
	Assigned				304	
	Total				238460	
FF-WJs527-b1a7	1	TRAV5	TRAJ8	CAEERTGFQKLVF	3815	66.95
	2	TRAV17	TRAJ40	CATAGWGTYKYIF	1349	23.67
	3	TRAV17	TRAJ40	CVTAGWGTYKYIF	117	2.05
	4	TRAV38-2/DV8	TRAJ12	CGMDSSYKLIF	24	0.42
	5	TRAV38-2/DV8	TRAJ48	CVSNFGNEKLTF	21	0.37
	In-frame				5698	
	Assigned				7253	
	Total				440363	
GPC3 T-iPSCs WT[36]	1	TRAV19	TRAJ10	CALTYILTGGGNKLTF	76811	29.29
	2	TRAV1-1	TRAJ33	CAVMDSNYQLIW	6540	2.49
	3	TRAV1-1	TRAJ31	CAVRDNNARLMF	5989	2.28
	4	TRAV9-2	TRAJ4	CALSDSGGYNKLIF	4624	1.76
	5	TRAV1-1	TRAJ34	CAVRDNTDKLIF	3937	1.50

	In-frame				262206	
	Assigned				358316	
	Total				415348	
GPC3 T-iPSCs RAG2 KO[36]	1	TRAV19	TRAJ10	CALTYILTGGGNKLTF	153535	86.31
	2	TRAV19	TRAJ10	CALTYIRTGGGNKLTF	1064	0.60
	3	TRAV19	TRAJ10	CALTDILTGGGNKLTF	764	0.43
	4	TRAV19	TRAJ10	CALTYILTGGGNKRTF	626	0.35
	5	TRAV19	TRAJ10	CALTYMLTGGGNKLTF	617	0.35
	In-frame				177884	
	Assigned				269603	
	Total				338666	
TkT3v1-7 WT[36]	1	TRAV38-2	TRAJ31	CAYWSNNNARLMF	27928	8.17
	2	TRAV39	TRAJ39	CAVVNNAGNMLTF	4270	1.25
	3	TRAV21	TRAJ11	CAVISSGYSTLTF	4122	1.21
	4	TRAV27	TRAJ22	CAGAGSARQLTF	3914	1.15
	5	TRAV30	TRAJ6	CGTSGGSYIPTF	3866	1.13
	In-frame				341648	
	Assigned				421059	
	Total				491249	
TkT3v1-7 RAG2 KO[36]	1	TRAV38-2	TRAJ31	CAYWSNNNARLMF	225909	91.85
	2	TRAV38-2	TRAJ31	CADWSNNNARLMF	747	0.30
	3	TRAV38-2	TRAJ31	CAYWSNSNARLMF	734	0.30
	4	TRAV38-2	TRAJ31	CDYWSNNNARLMF	637	0.26
	5	TRAV38-2	TRAJ31	CAYWSNNNVRLMF	545	0.22
	In-frame				245943	
	Assigned				250110	
	Total				325934	

Table S2. The details of sequencing read in the NGS-based TCRB gene expression analysis

In-frame: Number of reads for which the CDR3 region can be translated to amino acid sequences

Assigned: Number of reads judged as TCRA genes in the Repertoire Genesis software

Total: Number of total reads in NGS

%Read = (Number of reads) / (In-frame read number) x 100

Sample	Rank	TRBV	TRBJ	CDR3	Reads	%Read
FF-WJs524-Full-length	1	TRBV29-1	TRBJ2-2	CSVDGQGNTGELFF	31358	93.19
	2	TRBV29-1	TRBJ2-2	CSVDGQGNTEELFF	68	0.20
	3	TRBV29-1	TRBJ2-2	GSVDGQGNTGELFF	53	0.16
	4	TRBV29-1	TRBJ2-2	CSVDGQRNTGELFF	51	0.15
	5	TRBV29-1	TRBJ2-2	CSVEGQGNTGELFF	43	0.13
	In-frame				33651	
	Assigned				34908	
	Total				484086	
FF-WJs524-b1a1	1	TRBV28	TRBJ1-5	CASSNPGLKQPQHF	10	13.16
	2	TRBV29-1	TRBJ1-5	CSNQPQHF	5	6.58
	3	TRBV2	TRBJ1-5	CSNQPQHF	4	5.26
	4	TRBV9	TRBJ1-4	CATNEKLFF	2	2.63
	5	TRBV7-7	TRBJ1-5	CSNQPQHF	2	2.63
	In-frame				76	
	Assigned				548	
	Total				364343	
FF-WJs524-b1a7	1	TRBV9	TRBJ2-7	CASSVGERWTSGDEQYF	16	15.24
	2	TRBV29-1	TRBJ1-5	CSNQPQHF	6	5.71
	3	TRBV5-1	TRBJ1-5	CSNQPQHF	3	2.86
	4	TRBV7-2	TRBJ2-2	CANTGELFF	2	1.90
	5	TRBV7-2	TRBJ1-5	CSNQPQHF	2	1.90
	In-frame				105	
	Assigned				634	
	Total				339996	

FF-WJs527-Full-length	1	TRBV29-1	TRBJ2-2	CSVDGQGNTGELFF	13328	93.24
	2	TRBV20-1	TRBJ1-5	CSAGNPGTPAGPQHF	31	0.22
	3	TRBV29-1	TRBJ2-2	GSVDGQGNTGELFF	24	0.17
	4	TRBV29-1	TRBJ2-2	CSVDGQGNPGELFF	23	0.16
	5	TRBV29-1	TRBJ2-2	CSVEGQGNTGELFF	22	0.15
	In-frame				14294	
	Assigned				14853	
	Total				331814	
FF-WJs527-b1a1	1	TRBV7-2	TRBJ1-5	CSNQPQHF	5	8.06
	2	TRBV7-2	TRBJ2-2	CANTGELFF	3	4.84
	3	TRBV4-1	TRBJ1-5	CSNQPQHF	3	4.84
	4	TRBV5-4	TRBJ1-5	CSNQPQHF	2	3.23
	5	TRBV3-1	TRBJ1-5	CSNQPQHF	2	3.23
	In-frame				62	
	Assigned				572	
	Total				440286	
FF-WJs527-b1a7	1	TRBV28	TRBJ1-2	CASSRGMGGSGYTF	80	52.98
	2	TRBV29-1	TRBJ1-5	CSNQPQHF	6	3.97
	3	TRBV4-3	TRBJ1-5	CSNQPQHF	3	1.99
	4	TRBV6-3	TRBJ1-5	CSNQPQHF	2	1.32
	5	TRBV29/OR9-2	TRBJ1-5	CSNQPQHF	2	1.32
	In-frame				151	
	Assigned				538	
	Total				383582	

Table S3. cDNA sequences of the full-length TCR and the T2A peptide

Name	DNA sequence
TCRβ	ATGCTGAGTCTTCTGCTCCTTCTCCTGGGACTAGGCTCTGTGTTCAGTGC<br>TGTCATCTCTCAAAAGCCAAGCAGGGATATCTGTCAACGTGGAACCTCCC<br>TGACGATCCAGTGTCAAGTCGATAGCCAAGTCACCATGATGTTCTGGTAC<br>CGTCAGCAACCTGGACAGAGCCTGACACTGATCGCAACTGCAAATCAGG<br>GCTCTGAGGCCACATATGAGAGTGGATTTGTCATTGACAAGTTTCCCATC<br>AGCCGCCCAAACCTAACATTCTCAACTCTGACTGTGAGCAACATGAGCCC<br>TGAAGACAGCAGCATATATCTCTGCAGCGTTGATGGACAGGGAAACACCG<br>GGGAGCTGTTTTTTGGAGAAGGCTCTAGGCTGACCGTACTGGAGGACCT<br>GAAAAACGTGTTCCCACCCGAGGTCGCTGTGTTTGAGCCATCAGAAGCAG<br>AGATCTCCCACACCCAAAAGGCCACACTGGTGTGCCTGGCCACAGGCTT<br>CTACCCCGACCACGTGGAGCTGAGCTGGTGGGTGAATGGGAAGGAGGT<br>GCACAGTGGGGTCAGCACAGACCCGCAGCCCCTCAAGGAGCAGCCCGC<br>CCTCAATGACTCCAGATACTGCCTGAGCAGCCGCCTGAGGGTCTCGGCC<br>ACCTTCTGGCAGAACCCCCGCAACCACTTCCGCTGTCAAGTCCAGTTCTA<br>CGGGCTCTCGGAGAATGACGAGTGGACCCAGGATAGGGCCAAACCTGTC<br>ACCCAGATCGTCAGCGCCGAGGCCTGGGGTAGAGCAGACTGTGGCTTCA<br>CCTCCGAGTCTTACCAGCAAGGGGTCCTGTCTGCCACCATCCTCTATGAG<br>ATCTTGCTAGGGAAGGCCACCTTGTATGCCGTGCTGGTCAGTGCCCTCGT<br>GCTGATGGCCATGGTCAAGAGAAAGGATTCCAGAGGC
T2A peptide	TCCGGAAGCGGAGAGGGCAGAGGAAGTCTGCTAACATGCGGTGACGTCG<br>AGGAGAATCCTGGCCCC
TCRα	ATGGCATGCCCTGGCTTCCTGTGGGCACTTGTGATCTCCACCTGTCTTGA<br>ATTTAGCATGGCTCAGACAGTCACTCAGTCTCAACCAGAGATGTCTGTGC<br>AGGAGGCAGAGACCGTGACCCTGAGCTGCACATATGACACCAGTGAGAG<br>TGATTATTATTTATTCTGGTACAAGCAGCCTCCCAGCAGGCAGATGATTCT<br>CGTTATTCGCCAAGAAGCTTATAAGCAACAGAATGCAACAGAGAATCGTTT<br>CTCTGTGAACTTCCAGAAAGCAGCCAAATCCTTCAGTCTCAAGATCTCAGA<br>CTCACAGCTGGGGGATGCCGCGATGTATTTCTGTGCTTATTGGAGTAATA<br>ACAATGCCAGACTCATGTTTGGAGATGGAACTCAGCTGGTGGTGAAGCCC<br>AATATCCAGAACCCTGACCCTGCCGTGTACCAGCTGAGAGACTCTAAATC<br>CAGTGACAAGTCTGTCTGCCTATTCACCGATTTTGATTCTCAAACAAATGT<br>GTCACAAAGTAAGGATTCTGATGTGTATATCACAGACAAAACTGTGCTAGA<br>CATGAGGTCTATGGACTTCAAGAGCAACAGTGCTGTGGCCTGGAGCAACA<br>AATCTGACTTTGCATGTGCAAACGCCTTCAACAACAGCATTATTCCAGAAG

	ACACCTTCTTCCCCAGCCCAGAAAGTTCCTGTGATGTCAAGCTGGTCGAG AAAAGCTTTGAAACAGATACGAACCTAAACTTTCAAAACCTGTCAGTGATT GGGTTCCGAATCCTCCTCCTGAAAGTGGCCGGGTTTAATCTGCTCATGAC GCTGCGGCTGTGGTCCAGCTGA

Table S4. Amino acid sequences of the full-length-TCR and the T2A peptide

Name	Amino acid sequence
TCRβ	MLSLLLLLLGLGSVFSAVISQKPSRDICQRGTSLTIQCQVDSQVTMMFWYRQ QPGQSLTLIATANQGSEATYESGFVIDKFPISRPNLTFSTLTVSNMSPEDSSIY LCSVDGQGNTGELFFGEGSRLTVLEDLKNVFPPEVAVFEPSEAEISHTQKATL VCLATGFYPDHVELSWWVNGKEVHSGVSTDPQPLKEQPALNDSRYCLSSRL RVSATFWQNPRNHFRCQVQFYGLSENDEWTQDRAKPVTQIVSAEAWGRAD CGFTSESYQQGVLSATILYEILLGKATLYAVLVSALVLMAMVKRKDSRG
T2A peptide	SGSGEGRGSLLTCGDVEENPGP
TCRα	MACPGFLWALVISTCLEFSMAQTVTQSQPEMSVQEAETVTLSCTYDTSESDY YLFWYKQPPSRQMILVIRQEAYKQQNATENRFSVNFQKAAKSFSLKISDSQL GDAAMYFCAYWSNNNARLMFGDGTQLVVKPNIQNPDPAVYQLRDSKSSDK SVCLFTDFDSQTNVSQSKDSDVYITDKTVLDMRSMDFKSNSAVAWSNKSDF ACANAFNNSIIPEDTFFPSPESSCDVKLVEKSFETDTNLNFQNLSVIGFRILLLK VAGFNLLMTLRLWSS

Table S5. cDNA sequences of TCRα constructs

Name	Length	DNA sequence
α1	423	AATATCCAGAACCCTGACCCTGCCGTGTACCAGCTGAGAGACTCTA<br>AATCCAGTGACAAGTCTGTCTGCCTATTCACCGATTTTGATTCTCAA<br>ACAAATGTGTCACAAAGTAAGGATTCTGATGTGTATATCACAGACAA<br>AACTGTGCTAGACATGAGGTCTATGGACTTCAAGAGCAACAGTGCT<br>GTGGCCTGGAGCAACAAATCTGACTTTGCATGTGCAAACGCCTTCA<br>ACAACAGCATTATTCCAGAAGACACCTTCTTCCCCAGCCCAGAAAG<br>TTCCTGTGATGTCAAGCTGGTCGAGAAAAGCTTTGAAACAGATACG<br>AACCTAAACTTTCAAAACCTGTCAGTGATTGGGTTCCGAATCCTCCT<br>CCTGAAAGTGGCCGGGTTTAATCTGCTCATGACGCTGCGGCTGTG<br>GTCCAGC
α2	396	TACCAGCTGAGAGACTCTAAATCCAGTGACAAGTCTGTCTGCCTATT<br>CACCGATTTTGATTCTCAAACAAATGTGTCACAAAGTAAGGATTCTG<br>ATGTGTATATCACAGACAAAACTGTGCTAGACATGAGGTCTATGGA<br>CTTCAAGAGCAACAGTGCTGTGGCCTGGAGCAACAAATCTGACTTT<br>GCATGTGCAAACGCCTTCAACAACAGCATTATTCCAGAAGACACCT<br>TCTTCCCCAGCCCAGAAAGTTCCTGTGATGTCAAGCTGGTCGAGAA<br>AAGCTTTGAAACAGATACGAACCTAAACTTTCAAAACCTGTCAGTGA<br>TTGGGTTCCGAATCCTCCTCCTGAAAGTGGCCGGGTTTAATCTGCT<br>CATGACGCTGCGGCTGTGGTCCAGC
α3	363	TCTGTCTGCCTATTCACCGATTTTGATTCTCAAACAAATGTGTCACA<br>AAGTAAGGATTCTGATGTGTATATCACAGACAAAACTGTGCTAGACA<br>TGAGGTCTATGGACTTCAAGAGCAACAGTGCTGTGGCCTGGAGCAA<br>CAAATCTGACTTTGCATGTGCAAACGCCTTCAACAACAGCATTATTC<br>CAGAAGACACCTTCTTCCCCAGCCCAGAAAGTTCCTGTGATGTCAA<br>GCTGGTCGAGAAAAGCTTTGAAACAGATACGAACCTAAACTTTCAAA<br>ACCTGTCAGTGATTGGGTTCCGAATCCTCCTCCTGAAAGTGGCCGG<br>GTTTAATCTGCTCATGACGCTGCGGCTGTGGTCCAGC
α4	327	AATGTGTCACAAAGTAAGGATTCTGATGTGTATATCACAGACAAAAC<br>TGTGCTAGACATGAGGTCTATGGACTTCAAGAGCAACAGTGCTGTG<br>GCCTGGAGCAACAAATCTGACTTTGCATGTGCAAACGCCTTCAACA<br>ACAGCATTATTCCAGAAGACACCTTCTTCCCCAGCCCAGAAAGTTC<br>CTGTGATGTCAAGCTGGTCGAGAAAAGCTTTGAAACAGATACGAAC<br>CTAAACTTTCAAAACCTGTCAGTGATTGGGTTCCGAATCCTCCTCCT<br>GAAAGTGGCCGGGTTTAATCTGCTCATGACGCTGCGGCTGTGGTC

		CAGC
α5	303	GATGTGTATATCACAGACAAAACTGTGCTAGACATGAGGTCTATGG ACTTCAAGAGCAACAGTGCTGTGGCCTGGAGCAACAAATCTGACTT TGCATGTGCAAACGCCTTCAACAACAGCATTATTCCAGAAGACACC TTCTTCCCCAGCCCAGAAAGTTCCTGTGATGTCAAGCTGGTCGAGA AAAGCTTTGAAACAGATACGAACCTAAACTTTCAAAACCTGTCAGTG ATTGGGTTCCGAATCCTCCTCCTGAAAGTGGCCGGGTTTAATCTGC TCATGACGCTGCGGCTGTGGTCCAGC
α6	276	CTAGACATGAGGTCTATGGACTTCAAGAGCAACAGTGCTGTGGCCT GGAGCAACAAATCTGACTTTGCATGTGCAAACGCCTTCAACAACAG CATTATTCCAGAAGACACCTTCTTCCCCAGCCCAGAAAGTTCCTGT GATGTCAAGCTGGTCGAGAAAAGCTTTGAAACAGATACGAACCTAA ACTTTCAAAACCTGTCAGTGATTGGGTTCCGAATCCTCCTCCTGAAA GTGGCCGGGTTTAATCTGCTCATGACGCTGCGGCTGTGGTCCAGC
α7	252	AAGAGCAACAGTGCTGTGGCCTGGAGCAACAAATCTGACTTTGCAT GTGCAAACGCCTTCAACAACAGCATTATTCCAGAAGACACCTTCTTC CCCAGCCCAGAAAGTTCCTGTGATGTCAAGCTGGTCGAGAAAAGCT TTGAAACAGATACGAACCTAAACTTTCAAAACCTGTCAGTGATTGGG TTCCGAATCCTCCTCCTGAAAGTGGCCGGGTTTAATCTGCTCATGA CGCTGCGGCTGTGGTCCAGC
α8	228	AGCAACAAATCTGACTTTGCATGTGCAAACGCCTTCAACAACAGCAT TATTCCAGAAGACACCTTCTTCCCCAGCCCAGAAAGTTCCTGTGAT GTCAAGCTGGTCGAGAAAAGCTTTGAAACAGATACGAACCTAAACT TTCAAAACCTGTCAGTGATTGGGTTCCGAATCCTCCTCCTGAAAGT GGCCGGGTTTAATCTGCTCATGACGCTGCGGCTGTGGTCCAGC
α9	207	TGTGCAAACGCCTTCAACAACAGCATTATTCCAGAAGACACCTTCTT CCCCAGCCCAGAAAGTTCCTGTGATGTCAAGCTGGTCGAGAAAAGC TTTGAAACAGATACGAACCTAAACTTTCAAAACCTGTCAGTGATTGG GTTCCGAATCCTCCTCCTGAAAGTGGCCGGGTTTAATCTGCTCATG ACGCTGCGGCTGTGGTCCAGC
α10	195	TTCAACAACAGCATTATTCCAGAAGACACCTTCTTCCCCAGCCCAGA AAGTTCCTGTGATGTCAAGCTGGTCGAGAAAAGCTTTGAAACAGAT ACGAACCTAAACTTTCAAAACCTGTCAGTGATTGGGTTCCGAATCCT CCTCCTGAAAGTGGCCGGGTTTAATCTGCTCATGACGCTGCGGCTG TGGTCCAGC

α11	180	ATTCCAGAAGACACCTTCTTCCCCAGCCCAGAAAGTTCCTGTGATG<br>TCAAGCTGGTCGAGAAAAGCTTTGAAACAGATACGAACCTAAACTTT<br>CAAAACCTGTCAGTGATTGGGTTCCGAATCCTCCTCCTGAAAGTGG<br>CCGGGTTTAATCTGCTCATGACGCTGCGGCTGTGGTCCAGC

Table S6. cDNA sequences of TCRβ constructs

Name	Length	DNA sequence
β1	537	GAGGACCTGAAAAACGTGTTCCCACCCGAGGTCGCTGTGTTTGAG<br>CCATCAGAAGCAGAGATCTCCCACACCCAAAAGGCCACACTGGTGT<br>GCCTGGCCACAGGCTTCTACCCCGACCACGTGGAGCTGAGCTGGT<br>GGGTGAATGGGAAGGAGGTGCACAGTGGGGTCAGCACAGACCCG<br>CAGCCCCTCAAGGAGCAGCCCGCCCTCAATGACTCCAGATACTGC<br>CTGAGCAGCCGCCTGAGGGTCTCGGCCACCTTCTGGCAGAACCCC<br>CGCAACCACTTCCGCTGTCAAGTCCAGTTCTACGGGCTCTCGGAGA<br>ATGACGAGTGGACCCAGGATAGGGCCAAACCTGTCACCCAGATCG<br>TCAGCGCCGAGGCCTGGGGTAGAGCAGACTGTGGCTTCACCTCCG<br>AGTCTTACCAGCAAGGGGTCCTGTCTGCCACCATCCTCTATGAGAT<br>CTTGCTAGGGAAGGCCACCTTGTATGCCGTGCTGGTCAGTGCCCT<br>CGTGCTGATGGCCATGGTCAAGAGAAAGGATTCCAGAGGC
β2	489	TCAGAAGCAGAGATCTCCCACACCCAAAAGGCCACACTGGTGTGC<br>CTGGCCACAGGCTTCTACCCCGACCACGTGGAGCTGAGCTGGTGG<br>GTGAATGGGAAGGAGGTGCACAGTGGGGTCAGCACAGACCCGCA<br>GCCCCTCAAGGAGCAGCCCGCCCTCAATGACTCCAGATACTGCCT<br>GAGCAGCCGCCTGAGGGTCTCGGCCACCTTCTGGCAGAACCCCCG<br>CAACCACTTCCGCTGTCAAGTCCAGTTCTACGGGCTCTCGGAGAAT<br>GACGAGTGGACCCAGGATAGGGCCAAACCTGTCACCCAGATCGTC<br>AGCGCCGAGGCCTGGGGTAGAGCAGACTGTGGCTTCACCTCCGAG<br>TCTTACCAGCAAGGGGTCCTGTCTGCCACCATCCTCTATGAGATCT<br>TGCTAGGGAAGGCCACCTTGTATGCCGTGCTGGTCAGTGCCCTCG<br>TGCTGATGGCCATGGTCAAGAGAAAGGATTCCAGAGGC
β3	459	GCCACACTGGTGTGCCTGGCCACAGGCTTCTACCCCGACCACGTG<br>GAGCTGAGCTGGTGGGTGAATGGGAAGGAGGTGCACAGTGGGGT<br>CAGCACAGACCCGCAGCCCCTCAAGGAGCAGCCCGCCCTCAATGA<br>CTCCAGATACTGCCTGAGCAGCCGCCTGAGGGTCTCGGCCACCTT<br>CTGGCAGAACCCCCGCAACCACTTCCGCTGTCAAGTCCAGTTCTAC<br>GGGCTCTCGGAGAATGACGAGTGGACCCAGGATAGGGCCAAACCT<br>GTCACCCAGATCGTCAGCGCCGAGGCCTGGGGTAGAGCAGACTGT<br>GGCTTCACCTCCGAGTCTTACCAGCAAGGGGTCCTGTCTGCCACCA<br>TCCTCTATGAGATCTTGCTAGGGAAGGCCACCTTGTATGCCGTGCT<br>GGTCAGTGCCCTCGTGCTGATGGCCATGGTCAAGAGAAAGGATTC<br>CAGAGGC

β4	417	GTGGAGCTGAGCTGGTGGGTGAATGGGAAGGAGGTGCACAGTGG GGTCAGCACAGACCCGCAGCCCCTCAAGGAGCAGCCCGCCCTCAA TGACTCCAGATACTGCCTGAGCAGCCGCCTGAGGGTCTCGGCCAC CTTCTGGCAGAACCCCCGCAACCACTTCCGCTGTCAAGTCCAGTTC TACGGGCTCTCGGAGAATGACGAGTGGACCCAGGATAGGGCCAAA CCTGTCACCCAGATCGTCAGCGCCGAGGCCTGGGGTAGAGCAGAC TGTGGCTTCACCTCCGAGTCTTACCAGCAAGGGGTCCTGTCTGCCA CCATCCTCTATGAGATCTTGCTAGGGAAGGCCACCTTGTATGCCGT GCTGGTCAGTGCCCTCGTGCTGATGGCCATGGTCAAGAGAAAGGA TTCCAGAGGC
β5	399	GTGAATGGGAAGGAGGTGCACAGTGGGGTCAGCACAGACCCGCA GCCCCTCAAGGAGCAGCCCGCCCTCAATGACTCCAGATACTGCCT GAGCAGCCGCCTGAGGGTCTCGGCCACCTTCTGGCAGAACCCCCG CAACCACTTCCGCTGTCAAGTCCAGTTCTACGGGCTCTCGGAGAAT GACGAGTGGACCCAGGATAGGGCCAAACCTGTCACCCAGATCGTC AGCGCCGAGGCCTGGGGTAGAGCAGACTGTGGCTTCACCTCCGAG TCTTACCAGCAAGGGGTCCTGTCTGCCACCATCCTCTATGAGATCT TGCTAGGGAAGGCCACCTTGTATGCCGTGCTGGTCAGTGCCCTCG TGCTGATGGCCATGGTCAAGAGAAAGGATTCCAGAGGC
β6	372	GTCAGCACAGACCCGCAGCCCCTCAAGGAGCAGCCCGCCCTCAAT GACTCCAGATACTGCCTGAGCAGCCGCCTGAGGGTCTCGGCCACC TTCTGGCAGAACCCCCGCAACCACTTCCGCTGTCAAGTCCAGTTCT ACGGGCTCTCGGAGAATGACGAGTGGACCCAGGATAGGGCCAAAC CTGTCACCCAGATCGTCAGCGCCGAGGCCTGGGGTAGAGCAGACT GTGGCTTCACCTCCGAGTCTTACCAGCAAGGGGTCCTGTCTGCCAC CATCCTCTATGAGATCTTGCTAGGGAAGGCCACCTTGTATGCCGTG CTGGTCAGTGCCCTCGTGCTGATGGCCATGGTCAAGAGAAAGGATT CCAGAGGC
β7	336	GCCCTCAATGACTCCAGATACTGCCTGAGCAGCCGCCTGAGGGTC TCGGCCACCTTCTGGCAGAACCCCCGCAACCACTTCCGCTGTCAA GTCCAGTTCTACGGGCTCTCGGAGAATGACGAGTGGACCCAGGAT AGGGCCAAACCTGTCACCCAGATCGTCAGCGCCGAGGCCTGGGGT AGAGCAGACTGTGGCTTCACCTCCGAGTCTTACCAGCAAGGGGTC CTGTCTGCCACCATCCTCTATGAGATCTTGCTAGGGAAGGCCACCT TGTATGCCGTGCTGGTCAGTGCCCTCGTGCTGATGGCCATGGTCAA GAGAAAGGATTCCAGAGGC

β8	303	CGCCTGAGGGTCTCGGCCACCTTCTGGCAGAACCCCCGCAACCAC TTCCGCTGTCAAGTCCAGTTCTACGGGCTCTCGGAGAATGACGAGT GGACCCAGGATAGGGCCAAACCTGTCACCCAGATCGTCAGCGCCG AGGCCTGGGGTAGAGCAGACTGTGGCTTCACCTCCGAGTCTTACC AGCAAGGGGTCCTGTCTGCCACCATCCTCTATGAGATCTTGCTAGG GAAGGCCACCTTGTATGCCGTGCTGGTCAGTGCCCTCGTGCTGAT GGCCATGGTCAAGAGAAAGGATTCCAGAGGC
β9	276	CAGAACCCCCGCAACCACTTCCGCTGTCAAGTCCAGTTCTACGGGC TCTCGGAGAATGACGAGTGGACCCAGGATAGGGCCAAACCTGTCA CCCAGATCGTCAGCGCCGAGGCCTGGGGTAGAGCAGACTGTGGCT TCACCTCCGAGTCTTACCAGCAAGGGGTCCTGTCTGCCACCATCCT CTATGAGATCTTGCTAGGGAAGGCCACCTTGTATGCCGTGCTGGTC AGTGCCCTCGTGCTGATGGCCATGGTCAAGAGAAAGGATTCCAGA GGC
β10	258	TTCCGCTGTCAAGTCCAGTTCTACGGGCTCTCGGAGAATGACGAGT GGACCCAGGATAGGGCCAAACCTGTCACCCAGATCGTCAGCGCCG AGGCCTGGGGTAGAGCAGACTGTGGCTTCACCTCCGAGTCTTACC AGCAAGGGGTCCTGTCTGCCACCATCCTCTATGAGATCTTGCTAGG GAAGGCCACCTTGTATGCCGTGCTGGTCAGTGCCCTCGTGCTGAT GGCCATGGTCAAGAGAAAGGATTCCAGAGGC
β11	237	TACGGGCTCTCGGAGAATGACGAGTGGACCCAGGATAGGGCCAAA CCTGTCACCCAGATCGTCAGCGCCGAGGCCTGGGGTAGAGCAGAC TGTGGCTTCACCTCCGAGTCTTACCAGCAAGGGGTCCTGTCTGCCA CCATCCTCTATGAGATCTTGCTAGGGAAGGCCACCTTGTATGCCGT GCTGGTCAGTGCCCTCGTGCTGATGGCCATGGTCAAGAGAAAGGA TTCCAGAGGC
β12	216	GAGTGGACCCAGGATAGGGCCAAACCTGTCACCCAGATCGTCAGC GCCGAGGCCTGGGGTAGAGCAGACTGTGGCTTCACCTCCGAGTCT TACCAGCAAGGGGTCCTGTCTGCCACCATCCTCTATGAGATCTTGC TAGGGAAGGCCACCTTGTATGCCGTGCTGGTCAGTGCCCTCGTGC TGATGGCCATGGTCAAGAGAAAGGATTCCAGAGGC
β13	198	GCCAAACCTGTCACCCAGATCGTCAGCGCCGAGGCCTGGGGTAGA GCAGACTGTGGCTTCACCTCCGAGTCTTACCAGCAAGGGGTCCTGT CTGCCACCATCCTCTATGAGATCTTGCTAGGGAAGGCCACCTTGTA TGCCGTGCTGGTCAGTGCCCTCGTGCTGATGGCCATGGTCAAGAG AAAGGATTCCAGAGGC

Table S7. Amino acid sequences of TCRα constructs

Name	Length	Amino acid sequence
α1	141	NIQNPDPAVYQLRDSKSSDKSVCLFTDFDSQTNVSQSKDSDVYITDKTVLDMRSMDFKSNSAVAWSNKSDFACANAFNNSIIPEDTFFPSPESSCDVKLVEKSFETDTNLNFQNLSVIGFRILLLKVAGFNLLMTLRLWSS
α2	132	YQLRDSKSSDKSVCLFTDFDSQTNVSQSKDSDVYITDKTVLDMRSMDFKSNSAVAWSNKSDFACANAFNNSIIPEDTFFPSPESSCDVKLVEKSFETDTNLNFQNLSVIGFRILLLKVAGFNLLMTLRLWSS
α3	121	SVCLFTDFDSQTNVSQSKDSDVYITDKTVLDMRSMDFKSNSAVAWSNKSDFACANAFNNSIIPEDTFFPSPESSCDVKLVEKSFETDTNLNFQNLSVIGFRILLLKVAGFNLLMTLRLWSS
α4	109	NVSQSKDSDVYITDKTVLDMRSMDFKSNSAVAWSNKSDFACANAFNNSIIPEDTFFPSPESSCDVKLVEKSFETDTNLNFQNLSVIGFRILLLKVAGFNLLMTLRLWSS
α5	101	DVYITDKTVLDMRSMDFKSNSAVAWSNKSDFACANAFNNSIIPEDTFFPSPESSCDVKLVEKSFETDTNLNFQNLSVIGFRILLLKVAGFNLLMTLRLWSS
α6	92	LDMRSMDFKSNSAVAWSNKSDFACANAFNNSIIPEDTFFPSPESSCDVKLVEKSFETDTNLNFQNLSVIGFRILLLKVAGFNLLMTLRLWSS
α7	84	KSNSAVAWSNKSDFACANAFNNSIIPEDTFFPSPESSCDVKLVEKSFETDTNLNFQNLSVIGFRILLLKVAGFNLLMTLRLWSS
α8	76	SNKSDFACANAFNNSIIPEDTFFPSPESSCDVKLVEKSFETDTNLNFQNLSVIGFRILLLKVAGFNLLMTLRLWSS
α9	69	CANAFNNSIIPEDTFFPSPESSCDVKLVEKSFETDTNLNFQNLSVIGFRILLLKVAGFNLLMTLRLWSS
α10	65	FNNSIIPEDTFFPSPESSCDVKLVEKSFETDTNLNFQNLSVIGFRILLLKVAGFNLLMTLRLWSS
α11	60	IPEDTFFPSPESSCDVKLVEKSFETDTNLNFQNLSVIGFRILLLKVAGFNLLMTLRLWSS

Table S8. Amino acid sequences of TCRβ constructs

Name	Length	Amino acid sequence
β1	179	EDLKNVFPPEVAVFEPSEAEISHTQKATLVCLATGFYPDHVELSWWVNGKEVHSGVSTDPQPLKEQPALNDSRYCLSSRLRVSATFWQNPRNHFRCQVQFYGLSENDEWTQDRAKPVTQIVSAEAWGRADCGFTSESYQQGVLSATILYEILLGKATLYAVLVSALVLMAMVKRKDSRG
β2	163	SEAEISHTQKATLVCLATGFYPDHVELSWWVNGKEVHSGVSTDPQPLKEQPALNDSRYCLSSRLRVSATFWQNPRNHFRCQVQFYGLSENDEWTQDRAKPVTQIVSAEAWGRADCGFTSESYQQGVLSATILYEILLGKATLYAVLVSALVLMAMVKRKDSRG
β3	153	ATLVCLATGFYPDHVELSWWVNGKEVHSGVSTDPQPLKEQPALNDSRYCLSSRLRVSATFWQNPRNHFRCQVQFYGLSENDEWTQDRAKPVTQIVSAEAWGRADCGFTSESYQQGVLSATILYEILLGKATLYAVLVSALVLMAMVKRKDSRG
β4	139	VELSWWVNGKEVHSGVSTDPQPLKEQPALNDSRYCLSSRLRVSATFWQNPRNHFRCQVQFYGLSENDEWTQDRAKPVTQIVSAEAWGRADCGFTSESYQQGVLSATILYEILLGKATLYAVLVSALVLMAMVKRKDSRG
β5	133	VNGKEVHSGVSTDPQPLKEQPALNDSRYCLSSRLRVSATFWQNPRNHFRCQVQFYGLSENDEWTQDRAKPVTQIVSAEAWGRADCGFTSESYQQGVLSATILYEILLGKATLYAVLVSALVLMAMVKRKDSRG
β6	124	VSTDPQPLKEQPALNDSRYCLSSRLRVSATFWQNPRNHFRCQVQFYGLSENDEWTQDRAKPVTQIVSAEAWGRADCGFTSESYQQGVLSATILYEILLGKATLYAVLVSALVLMAMVKRKDSRG
β7	112	ALNDSRYCLSSRLRVSATFWQNPRNHFRCQVQFYGLSENDEWTQDRAKPVTQIVSAEAWGRADCGFTSESYQQGVLSATILYEILLGKATLYAVLVSALVLMAMVKRKDSRG
β8	101	RLRVSATFWQNPRNHFRCQVQFYGLSENDEWTQDRAKPVTQIVSAEAWGRADCGFTSESYQQGVLSATILYEILLGKATLYAVLVSALVLMAMVKRKDSRG
β9	92	QNPRNHFRCQVQFYGLSENDEWTQDRAKPVTQIVSAEAWGRADCGFTSESYQQGVLSATILYEILLGKATLYAVLVSALVLMAMVKRKDSRG
β10	86	FRCQVQFYGLSENDEWTQDRAKPVTQIVSAEAWGRADCGFTSESYQQGVLSATILYEILLGKATLYAVLVSALVLMAMVKRKDSRG
β11	79	YGLSENDEWTQDRAKPVTQIVSAEAWGRADCGFTSESYQQGVLSATILYEILLGKATLYAVLVSALVLMAMVKRKDSRG

β12	72	EWTQDRAKPVTQIVSAEAWGRADCGFTSESYQQGVLSATILYEILLGKATLYAVLVSALVLMAMVKRKDSRG
β13	66	AKPVTQIVSAEAWGRADCGFTSESYQQGVLSATILYEILLGKATLYAVLVSALVLMAMVKRKDSRG

Table S9. Primer sequences for inverse PCR

Construct	Forward Primer	Reverse Primer
α2	TACCAGCTGAGAGACTCTAAATCC	AGCCATGCTAAATTCAAGACAGGTG
α3	TCTGTCTGCCTATTCACCGATTTTG	
α4	AATGTGTCACAAAGTAAGGATTCTGATGTG	
α5	GATGTGTATATCACAGACAAAACTGTGCTAG	
α6	CTAGACATGAGGTCTATGGACTTCAAG	
α7	AAGAGCAACAGTGCTGTGGCC	
α8	AGCAACAAATCTGACTTTGCATGTG	
α9	TGTGCAAACGCCTTCAACAACAG	
α10	TTCAACAACAGCATTATTCCAGAAGAC	
α11	ATTCCAGAAGACACCTTCTTCCCC	
β2	TCAGAAGCAGAGATCTCCCACACCC	ACTGAACACAGAGCCTAGTCCCAG
β3	GCCACACTGGTGTGCCTGGCC	
β4	GTGGAGCTGAGCTGGTGGGTG	
β5	GTGAATGGGAAGGAGGTGCACAGTG	
β6	GTCAGCACAGACCCGCAGCCCC	
β7	GCCCTCAATGACTCCAGATACTGCC	
β8	CGCCTGAGGGTCTCGGCCAC	
β9	CAGAACCCCCGCAACCACTTCC	
β10	TTCCGCTGTCAAGTCCAGTTCTACG	
β11	TACGGGCTCTCGGAGAATGACG	
β12	GAGTGGACCCAGGATAGGGCCAAACC	
β13	GCCAAACCTGTCACCCAGATCGTC	
