# Supplementary material for: Mini-TCRs: Truncated T cell receptors to generate T cells from induced pluripotent stem cells
Source: Mol Ther Methods Clin Dev. 2023 Sep 16;31:101109. doi: 10.1016/j.omtm.2023.101109 (PMC10562677; doi:10.1016/j.omtm.2023.101109)
Supplement: Document S2. Article plus supplemental information [file mmc2.pdf]

Molecular Therapy
Methods & Clinical Development
Original Article

Mini-TCRs: Truncated T cell receptors to generate T cells from induced pluripotent stem cells

Shin-ichiro Takayanagi,[1,2,5] Bo Wang,[2,4,5] Saki Hasegawa,[1,2,5] Satoshi Nishikawa,[3,5] Ken Fukumoto,[1,2,5] Kohei Nakano,[4] Sayaka Chuganji,[1,2] Yuya Kato,[1] Sanae Kamibayashi,[2] Atsutaka Minagawa,[2] Atsushi Kunisato,[1] Hajime Nozawa,[1] and Shin Kaneko[2,4]

[1]Kirin Central Research Institute, Kirin Holdings Company, Ltd., 26-1, Muraoka-Higashi 2, Fujisawa-shi, Kanagawa 251-8555, Japan; [2]Shin Kaneko Laboratory, Department of Cell Growth and Differentiation, Center for iPS Cell Research and Application (CiRA), Kyoto University, 53 Kawahara-cho, Shogoin, Sakyo-ku, Kyoto 606-8507, Japan; [3]R&D Division, Kyowa Kirin Co. Ltd, 3-6-6 Asahi-machi, Machida-shi, Tokyo 194-8533, Japan; [4]Shinobi Therapeutics, Inc., 46-29 Yoshida-Shimo-Adachi-cho, Sakyo-ku, Kyoto 606-8501, Japan

Allogeneic T cell platforms utilizing induced pluripotent stem cell (iPSC) technology exhibit significant promise for the facilitation of adoptive immunotherapies. While mature T cell receptor (TCR) signaling plays a crucial role in generating T cells from iPSCs, the introduction of exogenous mature TCR genes carries a potential risk of causing graft-versus-host disease (GvHD). In this study, we present the development of truncated TCRα and TCRβ chains, termed mini-TCRs, which lack variable domains responsible for recognizing human leukocyte antigen (HLA)-peptide complexes. We successfully induced cytotoxic T lymphocytes (CTLs) from iPSCs by employing mini-TCRs. Combinations of TCRα and TCRβ fragments were screened from mini-TCR libraries based on the surface localization of CD3 proteins and their ability to transduce T cell signaling. Consequently, mini-TCR-expressing iPSCs underwent physiological T cell development, progressing from the CD4 and CD8 double-positive stage to the CD8 single-positive stage. The resulting iPSC-derived CTLs exhibited comparable cytokine production and cytotoxicity in comparison to that of full-length TCR-expressing T lymphocytes when chimeric antigen receptors (CARs) were expressed. These findings demonstrate the potential of mini-TCR-carrying iPSCs as a versatile platform for CAR T cell therapy, offering a promising avenue for advancing adoptive immunotherapies.

INTRODUCTION

Recent advances in cancer immunotherapies have extended existing therapeutic options such as surgery, chemotherapy, and radiotherapy for patients.[1,2] In particular, CAR T cell therapies have shown clinical outcomes in patients with B cell malignancies.[3,4] However, current autologous CAR T cell therapies have issues, such as a time lag until treatment and variability in population, that can occasionally lead to treatment failures.[5–8] A promising solution to these problems is to use allogeneic T cells from a healthy donor source and provide them in an off-the-shelf style.[9,10]

In addition to using donor-derived T cells, technologies based on feeder-free differentiation of induced pluripotent stem cells (iPSCs) have found clinical application and could become a mainstream adoptive immunotherapy in the future.[9,11–13] Both iPSC-derived and peripheral-blood-derived CAR T cells demonstrate comparable antitumor efficacy in mouse models.[14] Undifferentiated iPSCs are expandable, and CAR-expressing iPSCs can proliferate and be established as master cells.[15] Furthermore, gene editing against human leukocyte antigen (HLA) genes can universalize master cells to all patients.[16] Therefore, we focused on iPSC-derived T cells as host cells for immunotherapies.

Physiologically, T cell receptors (TCRs) play a crucial role in the immune system by interacting with antigen-presenting cells (APCs) and are involved in T cell development.[17] Several groups have created T cell-derived iPSCs (T-iPSCs) that express mature TCR genes possessed by T cells and that have the potential to differentiate into T cells.[18,19] Additionally, overexpression of full-length TCR genes into non-T cell-derived iPSCs is sufficient to generate T cells.[20] However, cross-reactivity of endogenous or overexpressed TCRs on these allogeneic T cells could be a risk factor for graft-versus-host disease (GvHD),[10] and the cross-reactivity of a TCR specific for HLA-A*24:02/Wilms tumor 1(235–243) with HLA-B*57:01 has been reported.[21]

Received 8 July 2022; accepted 13 September 2023;
https://doi.org/10.1016/j.omtm.2023.101109.

[5]These authors contributed equally

Correspondence: Shin-ichiro Takayanagi, Kirin Central Research Institute, Kirin Holdings Company, Ltd., 26-1, Muraoka-Higashi 2, Fujisawa-shi, Kanagawa 251-8555, Japan.
E-mail: shinichiro.takayanagi.ky@kyowakirin.com
Correspondence: Shin Kaneko, MD, PhD, Shin Kaneko Laboratory, Department of Cell Growth and Differentiation, Center for iPS Cell Research and Application (CiRA), Kyoto University, Japan.
E-mail: kaneko.shin@cira.kyoto-u.ac.jp

This is an open access article under the CC BY license (http://creativecommons.org/licenses/by/4.0/).

In this study, we designed "mini-TCRs" comprising truncated TCRα and TCRβ that lacked variable regions but maintained TCR functions. These mini-TCRs recruited CD3 proteins to the cell surface and transduced T cell activation signals. Mini-TCR expression induced differentiation to CD8+ T cells from undifferentiated iPSCs and iPSC-derived hematopoietic stem/progenitor cells (HSPCs). The generated CD8+ T cells also demonstrated specific cytotoxicity of target cells when used for engineering CAR T cells. We propose this technology as a promising tool for developing allogeneic, off-the-shelf CAR T cell therapies.

RESULTS

Screening identified a mini-TCR with high CD3 recruitment activity

In the immunological synapse (the interface between T cells and APCs), TCRs form complexes with CD3 proteins and recognize HLA-antigen peptide complexes on APCs.[17,22] TCR proteins contain variable domains that determine the specificity of the TCR and constant domains to interact with CD3 proteins.[23] We designed several TCRs lacking the variable region that did not recognize antigens but that did transduce TCR signaling, and we designated them mini-TCRs (Figure 1A).

To identify the mini-TCRs that efficiently form complexes with CD3 proteins, we created mini-TCR libraries comprising various lengths of the constant regions of the TCRα and TCRβ chains and designed the cell-based screening system to measure the cell surface localization of the CD3 protein (Figure 1B). The N termini were selected mainly at the boundaries of motifs and domains based on previous studies[24–27] and protein crystal structures (Protein DataBank: 3QJF) (Figure 1C).[28] A signal peptide was added to the 5′ terminus of the designed TCR sequences that were tagged with Myc (for TCRα, TCRA-Myc) or FLAG (for TCRβ, TCRB-FLAG) epitopes at the 3′ terminus (Figure S1A). Intracellular staining with anti-Myc or anti-FLAG antibodies confirmed the expression of TCRA-Myc or TCRB-FLAG proteins from all vectors (Figures S1B and S1C, respectively). We then co-transfected all combinations of generated TCRA-Myc and TCRB-FLAG vectors into 293T cells expressing all CD3 genes (CD3-OE-293T cells; Figure S1D) to test whether they could translocate CD3 proteins to the cell surface (hereafter referred to as CD3 recruitment activity) (Figure 1B). CD3 recruitment activity was found to differ depending on the combination of TCRα and TCRβ (Figures 1D and 1E). Particularly high CD3 recruitment activities were observed when the full-length constant region of TCRβ was used irrespective of the length of a coupled TCRα, as well as in the case where both TCRα and TCRβ were short, truncated forms. The highest CD3 recruitment activity was achieved by combining α7 and β1 (92.4%). These results showed that even TCRs lacking the variable region can recruit CD3 proteins to the cell surface.

Based on CD3 recruitment capacities, we selected 17 representative pairs of truncated TCRα and TCRβ, including the pairs with the lowest (β6 and α1) and highest (β1 and α7) recruitment activities (Figures 1E and 2A). cDNAs of the selected TCRβ and TCRα were fused with the T2A peptide as previously reported and were subcloned into a single vector, as well as full-length TCRα and full-length TCRβ (Figures 2A and 2B).[29] The CD3 recruitment activities of the generated constructs in CD3-OE-293T showed a similar trend to that of the mini-TCRs that were transfected with separate TCRα and TCRβ expression vectors (Figure 2C).

To confirm the loss of antigen-recognition abilities due to deletion of the variable regions, we transfected the 17 pairs of mini-TCR vectors and the full-length TCR that recognized mutated Wilms-Tumor1 (WT1) peptide-HLA-A*24:02 complex as a positive control into CD3-OE-293T cells. In contrast to the binding of WT1-TCR-expressing cells to the WT1-HLA-A*24:02 tetramer, none of the mini-TCR-expressing cells exhibited binding to the tetramer, confirming that the mini-TCR did not recognize the antigen peptide-HLA complexes (Figures 2D and 2E). Based on these initial assessments, we used these 17 pairs of mini-TCR for further characterization.

Mini-TCR activated Jurkat T cells upon CD3 stimulation

TCR/CD3 complexes transduce TCR signaling and activate T cells.[30] Because 293T cells are not derived from T cells, TCR signaling-related molecules, including the CD3 genes, are considered to be negative. Therefore, we used human T cell-derived Jurkat cells to monitor activation upon CD3 stimulation as the second screening step.

To specifically detect transfected TCR proteins, we attempted to disrupt endogenous TCRA and TCRB genes using gene-editing technology. Both TCRα constant (TRAC) locus and TCRβ constant 1 (TRBC1) loci were targeted with previously reported guide RNAs (gRNAs) (Figure S2A). To confirm the disruption of the TCR genes, full-length TCRα and/or full-length TCRβ expression vectors were transfected into Jurkat cells, which were subsequently stained with an anti-CD3E antibody and an anti-TCRαβ antibody that recognized a part of the constant region of the TCRαβ complex. Although the CD3E and TCRαβ protein was detected after co-transfection of both full-length TCRα and full-length TCRβ, they were not detected by either single transfection of full-length TCRα or full-length TCRβ, suggesting that endogenous TCR genes were successfully disrupted (Figure S2B).

Subsequently, to compare CD3 recruitment activity, we transfected all 17 mini-TCR vectors, the full-length TCR control, and the empty vector as a negative control. Neither CD3D nor CD3E proteins were detected in the empty-vector control samples, confirming that CD3D and CD3E proteins did not localize on the cell surface unless the TCRA and TCRB chains were expressed in the Jurkat knockout (KO) cells (Figures 3A–3D). Cell surface localization of CD3D and CD3E proteins was detected in TCR-KO Jurkat cells with full-length TCR controls and several mini-TCRs, including b1a1 and b1a7, suggesting that these mini-TCRs formed a complex with CD3 proteins (Figures 3A–3D).

To assess the function of the mini-TCR/CD3 complex, the transfected TCR-KO Jurkat cells were stimulated with an OKT3 anti-CD3E

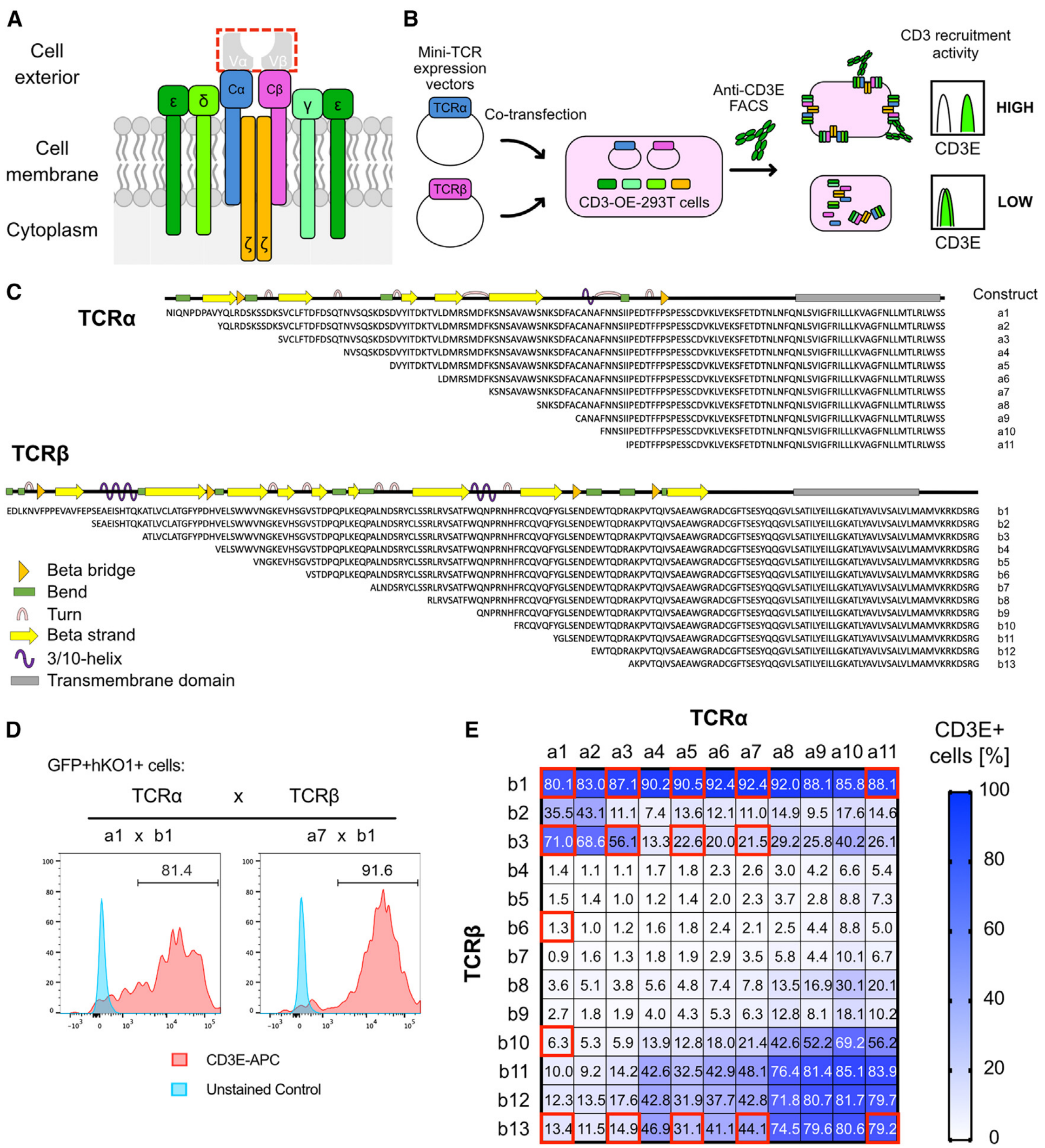

Figure 1. Design of mini-TCR constructs and their CD3 protein recruitment activities

(A) Schematic representation of the TCR/CD3 complex. The deletion of variable regions from TCRα and TCRβ chains depicts the concept of mini-TCR. Cα, constant region of TCRα; Cβ, constant region of TCRβ; Vα, variable region of TCRα; Vβ, variable region of TCRβ; ε, CD3E; γ, CD3G; δ, CD3D; and ζ, CD3Z. (B) The experimental scheme to screen the pairs of TCRα and TCRβ chains based on the CD3 recruitment activity. (C) Amino acid sequences and protein motifs of constant regions of TCRα (upper) and TCRβ (lower) chains. The constant regions were tagged with Myc (TCRα, TCRA-Myc) or FLAG (TCRβ, TCRB-FLAG) epitopes at the C terminus. (D) Representative plots of CD3-OE-293T cells transfected with TCRA-Myc and TCRB-FLAG. hKO1+GFP+ cells are shown. (E) The screening result of TCR constructs based on their CD3 protein recruitment activities. Values in the heatmap indicate the percentage of CD3E+ cells. Data are shown as mean of three independent experiments.

antibody. Subsequently, we observed the highest upregulation of CD69, a T cell activation marker, in the full-length TCR control, and the mini-TCR b1a7 showed comparable upregulation, followed by other mini-TCRs that showed correlation with mean fluorescence intensities (MFIs) of CD3D and CD3E (Figures 3E, 3F, and S2C).

As the binding affinity of the anti-CD3E antibody OKT3 to TCR/CD3 complex measured by bio-layer interferometry was approximately 10-fold higher than that of the extracellular domains of CD3γε,[27] suggesting that the presence of the TCR proteins affected the activity of the OKT3 antibody, we hypothesized that CD69 expression levels

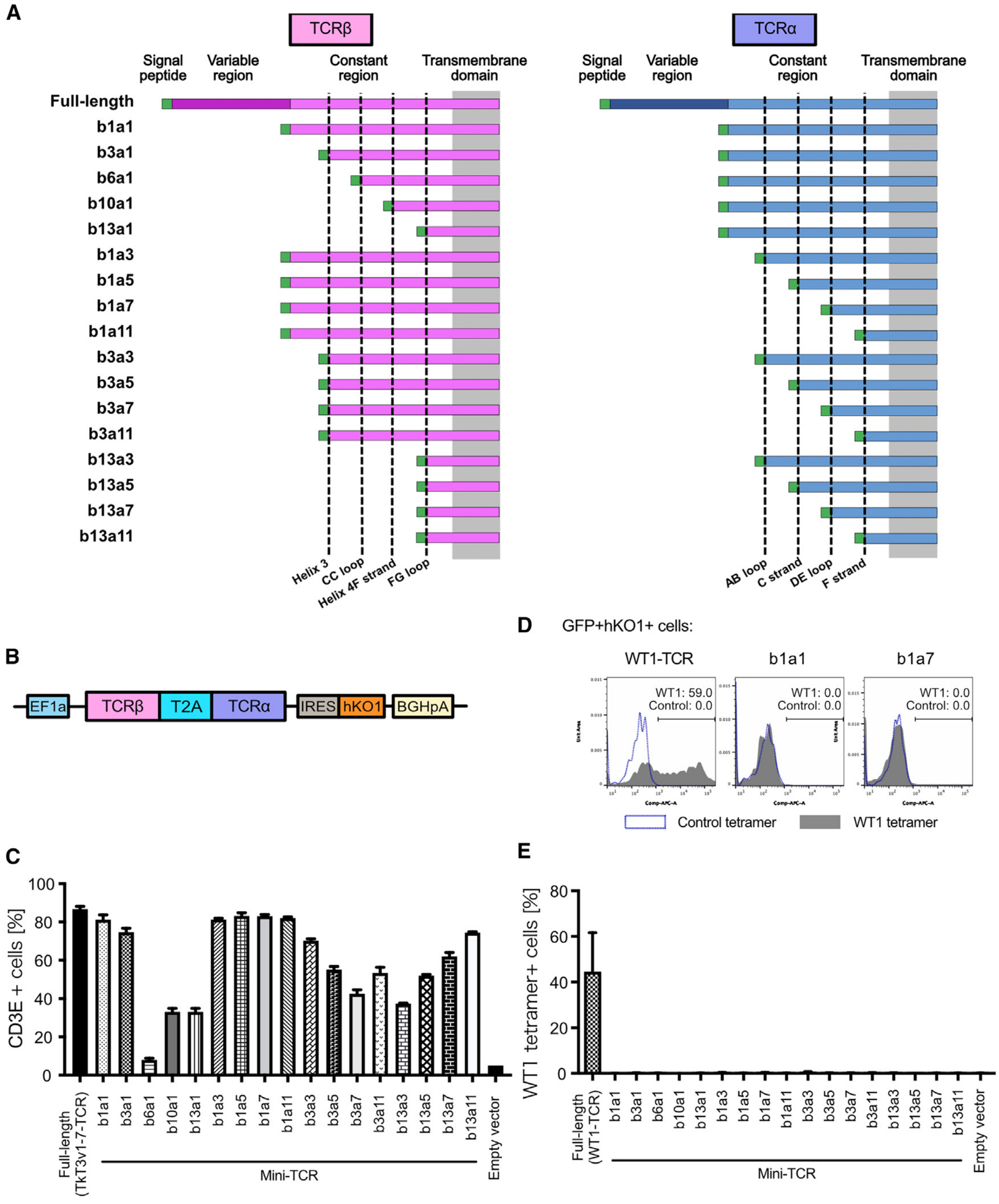

Figure 2. CD3 recruitment activities of mini-TCRs using a polycistronic expression vector

(A) Structures of the TCRα and TCRβ proteins in the generated vector. (B) The vector structure that combines TCRα and TCRβ into a single vector. The humanized Kusabiraorange1 (hKO1) fluorescent protein was used as a transfection marker. T2A, Thosea asigna virus 2A-like peptide. (C) CD3 protein recruitment activities of each created vector expressed in CD3-OE-293T cells. Data are shown as mean ± SD of three independent experiments. (D) Representative plots of TCR-transfected CD3-OE-293T cells stained with the Wilms Tumor1 (WT1)-TCR tetramer. (E) Mean fluorescence intensity (MFI) of the WT1-TCR tetramer. Data are shown as mean ± SD of three independent experiments.

were affected by the different binding capacities of OKT3 to the full-length TCR-CD3 and mini-TCR-CD3 complexes on the cell surface. To confirm this, mini-TCR (b1a7) (which showed the highest CD3 recruitment activity and CD69 expression among the mini-TCRs), mini-TCR (b1a1) (composed of the full-length TCRα and TCRβ constant regions with lower activities), and full-length TCR were each

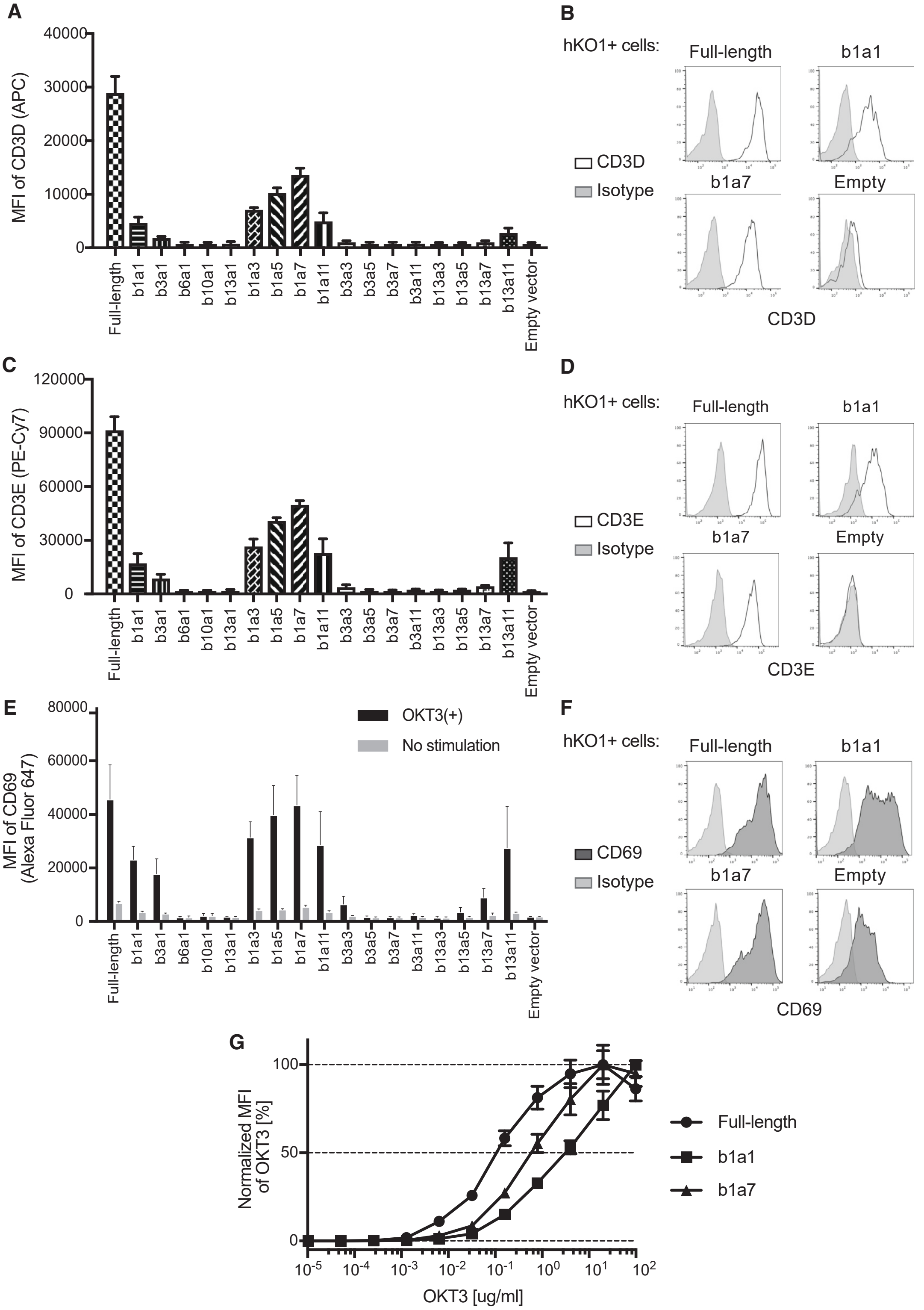

Figure 3. Activation of mini-TCR-expressing TCR-KO Jurkat cells via CD3 stimulation

MFI (A) and representative plots (B) of CD3D in TCR-KO Jurkat cells transiently transfected with TCR expression vectors. MFI (C) and representative plots (D) of CD3E in TCR-KO Jurkat cells transiently transfected with TCR expression vectors. MFI (E) and representative plots (F) of the CD69 T cell activation marker. TCR-KO Jurkat cells transiently

(legend continued on next page)

transfected into the TCR-KO Jurkat cells. The full-length TCR-expressing cells exhibited the highest MFI overall, followed by b1a7- and b1a1-expressing cells (Figure S2D), which correlated with the MFI of CD3E detected by anti-CD3E antibody UCHT1 (Figure 3C). Based on the normalized MFI of OKT3, the highest reactivity was observed against full-length TCR, followed by that against b1a7 and b1a1, suggesting the relatively low affinity of OKT3 against mini-TCRs (Figure 3G).

Collectively, these data demonstrated that the introduction of mini-TCRs enables endogenous CD3 proteins to localize on the cell surface and mediate TCR signaling via stimulation with the CD3 antibody. Subsequently, we evaluated the functions of mini-TCRs b1a1 and b1a7 in the differentiation of iPSCs to T cells.

Mini-TCRs enabled T cell differentiation of iPSC-derived HSPCs

The TCR/CD3 complex is also critical for T cell development. The T cell lineage commitment of common lymphoid progenitors derived from multipotent HSPCs is triggered by Notch signaling.[31] CD4 and CD8 double-negative (DN) T cells mature into double-positive (DP) cells, followed by further maturation into single-positive (SP) CD4 or SP CD8 cells in the thymus.[32] During maturation, T cells are selected based on their binding affinities between the self-peptide-HLA complex on thymic epithelial cells and the TCR/CD3 complex.[33]

We utilized cord-blood-derived iPSCs as a model of the hematopoietic system to test the function of mini-TCRs in T cell development, as these iPSCs were derived from myeloid cells and their TCR genes were not rearranged (Figure 4A). First, the iPSCs were differentiated into HSPCs via an embryoid body formation method, and retroviruses encoding full-length TCR, mini-TCRs b1a1 or b1a7, or empty-vector control were transduced into the harvested HSPCs (day −1; Figure S3A). The transduced cells were then cultured with a recombinant Delta-like ligand 4 (DLL4) protein, a ligand of Notch receptors, to trigger lineage commitment into T cells (days 0–21). There are no statistically significant differences in the MFI of GFP, which indicate transduced vector copy numbers, across the four retroviruses (Figure S3B).[34] Flow cytometry analyses showed that cell surface expression of CD3 was observed only in full-length TCR- and mini-TCR (b1a1 and b1a7)-transduced cells, whereas leukocyte common antigen CD45 was expressed in all samples, including empty-vector control cells (day 21; Figure 4B). Full-length TCR-transduced cells showed the highest MFI of CD3 (MFI = 29,377), followed by b1a7 (MFI = 14,483), b1a1 (MFI = 4,423), and the empty-vector control (MFI = 898; Figure 4C). Both full-length TCR and mini-TCR-expressing CD3+ cells included comparable percentages of CD4+CD8α+ DP cells, while the DP cells were not enriched (Figure 4D). These data indicated that introduction of mini-TCR genes enables efficient differentiation of iPSC-derived HSPCs into T cell lineage cells.

T cell activation signaling via the TCR/CD3 complex is essential for the maturation of DP cells to SP cells in the thymus.[33] To test the function of CD3E proteins on the mini-TCR-expressing DP cells during the maturation process, entire T cells, including DP cells, were further stimulated with an OKT3 anti-CD3 antibody in the presence of dexamethasone, a steroid that mimics endogenous glucocorticoids in the thymus (Figure 4A).[35,36] Selective activation of CD3+ cells led to nearly 100% enrichment of GFP+ cells (Figure S3C). The order of the MFI of CD3E on full-length- and mini-TCR-transduced cells after OKT3 stimulation was similar to that of DP T cells (Figures 4E and 4F). Notably, viable cell populations were not observed in empty-vector control samples after maturation (data not shown; no cell populations were observed in the forward scatter-side scatterplot of the flow cytometry analysis). Neither mini-TCR- nor full-length TCR-transduced cells expressed CD4 (Figure 4G); however, both expressed CD8α and CD8β, phenotypic markers of authentic cytotoxic T lymphocytes (CTLs; Figure 4H). These CTLs were expandable when peripheral blood mononuclear cells (PBMCs) were co-cultured as feeder cells, and entire cell yields per HSPC reached at least 10^5-fold (Figure 4I).

Avoiding GvHD risk is one of the primary purposes of the mini-TCRs. While the loss of antigen-recognition capacities by mini-TCR was experimentally demonstrated in Figure 2E, endogenous expression of TCRA and TCRB genes in the differentiated CTLs remains the risk associated with GvHD. We performed next-generation sequencing (NGS) of TCRA and TCRB transcripts, of which cDNA samples were amplified with the adapter primer at the 5′ terminus, and primers correspond to constant regions (Figure S4A). Then, sequenced reads were queried to the CDR3 sequence database to extract in-framed reads that can be translated to CDR3 proteins. It is worth noting that mini-TCR lacks variable domains, including CDR3, and the detected TCRA and TCRB transcripts could indicate endogenous TCR gene expression in mini-TCR-transduced CTLs. Among the nearly 3×10^5 total reads, 33.9% of in-frame TCRA reads and 4.95% of in-frame TCRB were detected in full-length TCR CTLs (mean values of two iPSCs) (Figure 4J). Mini-TCR CTLs exhibited very limited frequencies of in-frame TCRA reads (0.01%–1.29%) and in-frame TCRB reads (0.01%–0.03%) in both b1a1 and b1a7 mini-TCR CTLs (Figure 4J).

To determine the endogenous TCR gene expression, we referred to our previous work that demonstrated the disruption of RAG2-disabled rearrangement of TCR genes during T cell induction from two T cell-derived iPSCs (GPC3 T-iPSC and TkT3V1-7) using similar NGS-based TCR repertoire analysis with the similar depth of the sequencing (3.3–4.9 $\times 10^5$ total reads) (Table S1).[36] Among the 1.8 or 2.5 $\times 10^5$ total in-frame reads, 86.31% and 91.85% of the reads represented the original TCRA sequences in the RAG2-KO iPSC-derived T cells (GPC3 T-iPSC and TkT3V1-7, respectively) (Table S1),

transfected with TCR expression vectors were stimulated with anti-CD3E antibody OKT3 overnight and stained with the anti-CD69 antibody. (G) Normalized MFI of OKT3 in TCR-KO Jurkat cells transiently transfected with TCR expression vectors to compare the reactivity of OKT3. All data are shown as mean ± SD of three independent experiments.

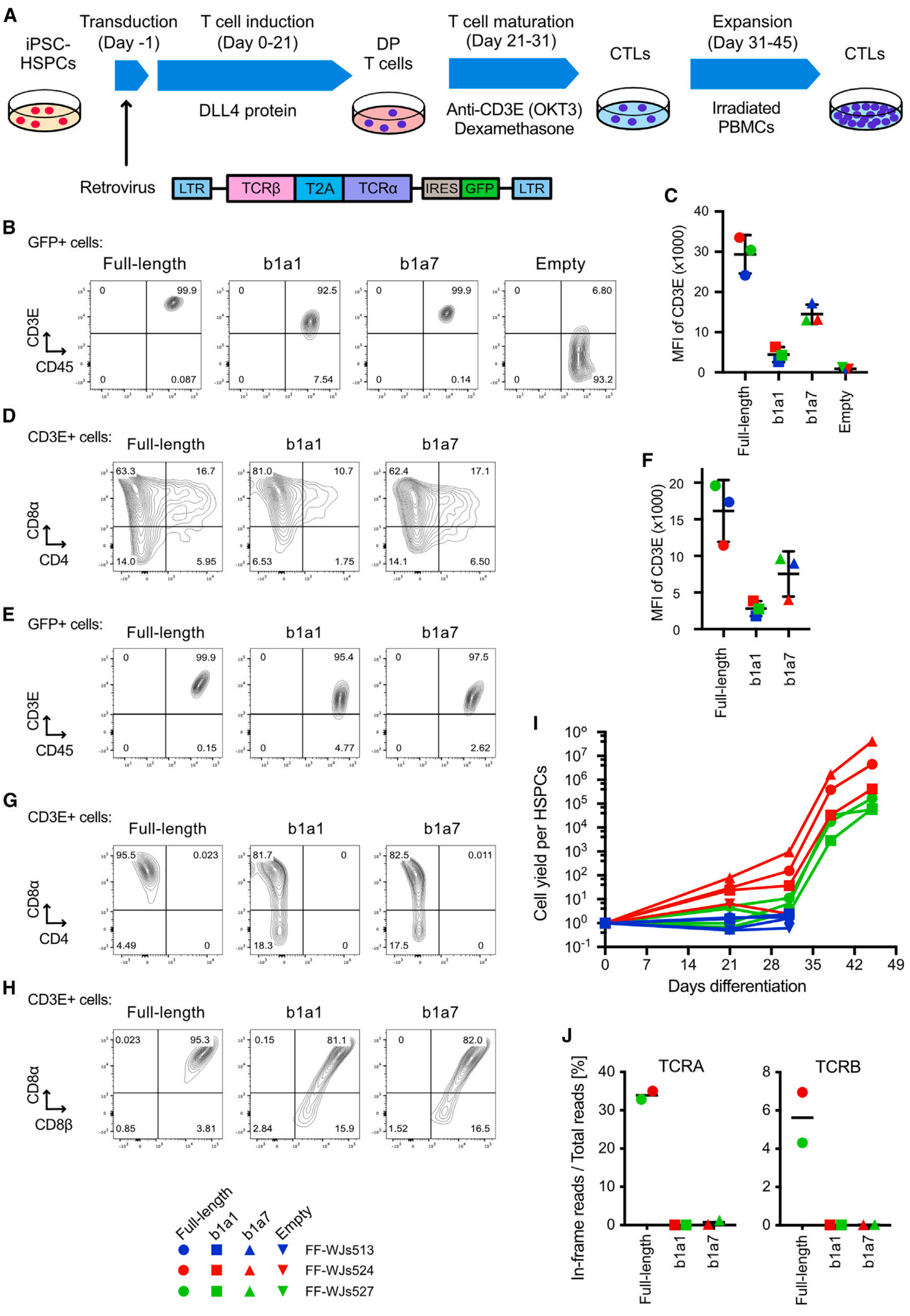

(legend on next page)

whereas only 1,000 to hundreds of reads encoded CDR3 amino acid sequences other than the original TCRA (Table S1, rank 2–5). Considering that RAG genes are vital for TCR rearrangement, hundreds of in-frame reads other than the original TCRs were regarded as sequencing noise levels. In this study, 96.47% and 93.22% of in-frame reads from full-length TCR CTLs were confirmed to be those of transduced full-length TCRA and TCRB, respectively (Tables S1, S2, and S3). Regarding the mini-TCR-transduced CTLs, the numbers of in-frame reads were also less than hundreds, except for b1a7-transduced FF-WJs527 cells, suggesting that mini-TCR suppressed the expression of endogenous TCR to the near noise level. Because CTLs induced with b1a7 exhibited a tendency toward a biased repertoire (Figure S4B; Tables S1 and S2), disrupting the RAG2 gene or TRAC locus would be worth considering to avoid the TCR rearrangement and endogenous TCR expression altogether. In conclusion, mini-TCRs demonstrated comparable performance to full-length TCR as inducers of phenotypic CTLs in iPSC-derived HSPCs.

Mini-TCR-expressing iPSCs differentiated into CTLs

We examined whether mini-TCRs could be used in undifferentiated iPSCs as a cell source for T cell generation (Figure 5A). To address this question, a clinical-grade iPSC clone[37] was transfected with mini-TCRs b1a1 and b1a7 using the piggyBac system by MaxCyte, and transgene-positive cells were isolated via fluorescence-activated cell sorting (FACS) (Figure S5). Concurrently, iPSCs transfected with a full-length Glypican-3 (GPC3)-specific TCR were generated as a positive control. The differentiation of both mini-TCR-iPSCs and full-length TCR-iPSCs into HSPCs was comparable to that of the parental iPSC line and the full-length TCR iPSC (Figure 5B). Furthermore, HSPCs derived from mini-TCR-iPSCs were able to differentiate into CD3+CD4+CD8α+ T cells, similar to full-length TCR-iPSCs, in a clinical-grade T cell induction condition (Figures 5C and 5D).[14] Furthermore, only progenitor cells derived from mini-TCR-iPSCs and full-length TCR-iPSCs responded to OKT3 stimulation and matured into CD8αβ SP CTLs (Figures 5E–5G).

Cell yields during the differentiation and expansion processes were estimated to reach the magnitude of 1×10^{10} cells from an initial 1×10^{6} iPSCs (full length, 6.84×10^{10}; b1a1, 8.58×10^{10}; b1a7, 4.35×10^{10}) (Figure 5H). Similar to full-length TCR-iPSC-derived T cells and consistent with our previously reported phenotype of iPSC-derived T cells, both b1a1 and b1a7 iPSC-derived T cells exhibited elevated expression levels of CD45RA, CD62L, and CD45RO, and partial expression levels of CD27 and CCR7 (Figure 5I).[14,38] It is worth noting that these CTL-expressing memory T cell markers have been shown to correlate significantly with antitumor efficacy in induced pluripotent stem (iPS)-T cells[14,38] as well as in peripheral blood-derived T cells.[39] Consequently, iPSCs expressing mini-TCR were considered to be a robust cell source for generating CTLs.

Mini-TCR-iPSC-derived T cells responded to the CD20 antigen when engineered with anti-CD20 CAR

To investigate whether such expanded CTLs could potentially be used for CAR T cell therapy, full-length or mini-TCR-iPSC-T cells were modified by transducing a retroviral vector containing a second-generation anti-CD20 CAR (CD20CAR) (Figures S6A and S6B). The effector function of these CAR-expressing cells was evaluated by co-culturing them with B lymphoblastoid cell line (B-LCL) cells expressing CD20 (Figures 6A and S6C). CD20CAR-expressing mini-TCR-iPSC-T cells exhibited significantly elevated expression of the degranulation marker CD107a compared to CD20CAR-negative populations (Figures 6B and 6C). Intracellular staining revealed that CD107a+ cells produced significantly elevated levels of interferon-gamma (IFNγ), implying that mini-TCR-iPSC-T cells executed their CTLs function in a CAR-dependent manner (Figure 6D). Moreover, after co-culturing with B-LCL cells for 3 h, the CD20CAR-transduced mini-TCR-iPSC-T cells (b1a7) efficiently killed the target cells, with comparable efficacy in comparison to the control CD20CAR-transduced full-length TCR-iPSC-T cells (Figure 6E). These results suggest that mini-TCR-iPSC-T cells expressing CAR exhibit robust effector function, thereby holding promise for potential utilization in CAR T cell therapy.

iPSC-CAR T cells generated using mini-TCRs had *in vivo* antitumor effects

Finally, we evaluated the antitumor effects of CTLs generated using the mini-TCR b1a7, which showed the highest CD3 recruitment activity and higher cytokine production in response to the CAR antigen (Figure 7A). Another second-generation CAR targeting CD19 (CD19CAR) was transduced into iPSC-derived T cells generated using mini-TCR b1a7 or full-length TCR (hereafter designated as b1a7 CD19CAR T cells or full-length TCR CD19CAR T cells, respectively) (Figure S7A). Surface expression of the CD19CAR protein was confirmed with protein-L staining (Figure S7B). Moreover, luciferase-expressing NALM6 (NALM6-Luc-KO) B-ALL cells were used as the target cells. The *in vitro* cytotoxicity against NALM6-Luc-KO

Figure 4. Induction of CTLs from iPSC-derived HSPCs by mini-TCRs

(A) Experimental scheme of T cell differentiation using TCR genes. For the stepwise differentiation processes from iPSC-derived HSPCs to CTLs, retroviruses encoding full-length TCR, mini-TCR, or empty control vectors were transduced into HSPCs. (B) Representative FACS plots of the CD45 and CD3E expression after T cell induction on DLL4 protein. (C) MFI of CD3E in GFP+ transduced cells after T cell induction on DLL4 protein. Data are shown as mean ± SD of three iPS clones. (D) Representative FACS plots of CD4 and CD8α in CD3E + cells after T cell induction. (E) Representative FACS plots of the CD45 and CD3E expression after T cell maturation using the anti-CD3E antibody and dexamethasone. (F) MFI of CD3E in GFP+ cells after T cell maturation. Data are shown as mean ± SD of three iPS clones. (G) Representative FACS plots of CD4 and CD8α expression after T cell maturation. (H) Representative FACS plots of CD8α and CD8β expression after T cell maturation. (I) Estimated cell yields per HSPC. Cell numbers were calculated based on the initially seeded HSPC count and subsequent proliferation rates. (J) TCR gene expression analysis by NGS. Percentages of in-frame reads coding CDR3 amino acid sequence derived from endogenous TCRA or TCRB genes are shown.

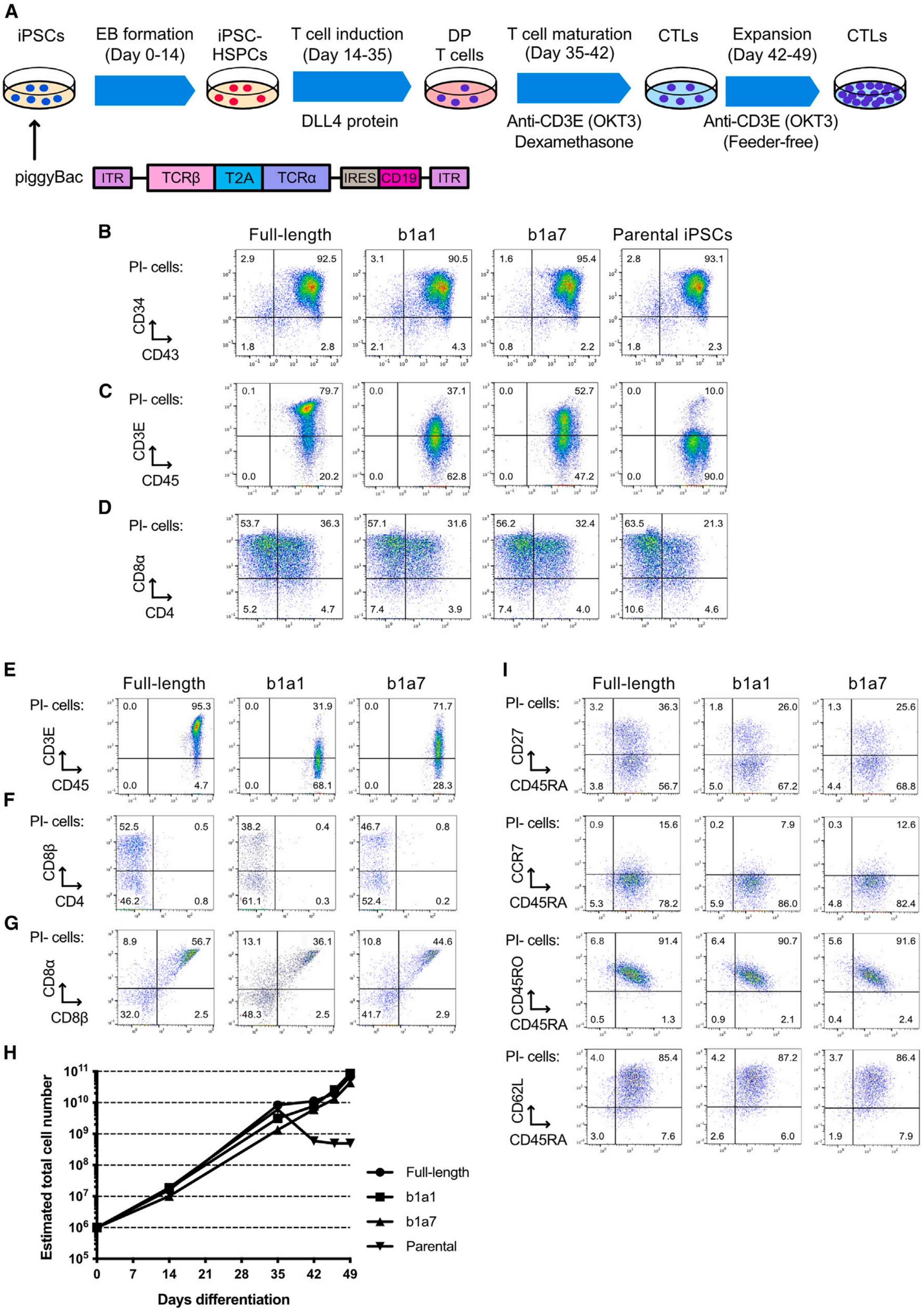

(legend on next page)

cells of b1a7 CD19CAR T cells was comparable to that of full-length TCR CD19CAR T cells (Figure 7B).

To evaluate the *in vivo* antitumor efficacy, NALM6-Luc-KO cells were injected to NOD.CG-Prkdc scid Il2rg tm1Wjl/SzJ (NSG) mice (day −3) (Figure 7A). The mice were grouped based on the amount of the engrafted tumor (day −1; Figures S7C and S7D). Subsequently, b1a7 CD19CAR or full-length TCR CD19CAR T cells were injected into the mice 3 days after injection of NALM6-Luc-KO cells (day 0). *In vivo* imaging at 3 weeks demonstrated that both b1a7 CD19CAR and full-length TCR CD19CAR T cells significantly suppressed the tumor growth of NALM6-Luc-KO cells compared to untreated control mice (total luminescence at 3 weeks: b1a7 CD19CAR T cells, 135.4×10^5 photons/s; full-length TCR CD19CAR T cells, 130.5×10^5 photons/s; untreated control cells, 336×10^5 photons/s; Figures 7C and 7D). A similar trend was observed at 4 weeks. These data indicated that iPSC-derived T cells generated using mini-TCRs can serve as an allogeneic cell source for CAR T cell therapy.

DISCUSSION

In this study, we developed mini-TCRs, gene constructs of truncated TCRs, proficient in inducing T cell differentiation from iPSCs. Furthermore, we substantiated an innovative approach for engineering allogeneic CAR T cell therapies. Mini-TCRs successfully triggered T cell differentiation in all four iPSC lines tested. These compelling findings strongly suggest that mini-TCRs have the potential to generate CD8+ T cells from iPSCs as a promising allogeneic cell source platform to enhance CAR T cell therapies. In this section, we elaborately compare the mini-TCRs with full-length TCR and other iPS-T cell-related technologies. Additionally, we explore the future perspectives of iPS-T cells in the context of our findings.

In the screening of mini-TCRs, constant regions of TCRα and TCRβ were cleaved in a stepwise manner, and the combination of these TCRs resulted in a bimodal distribution of CD3 recruitment to the cell surface (Figure 1C). One reason for this could be a change in the interaction between TCRα and TCRβ.[40] TCRα and TCRβ are held by hydrogen and disulfide bonds in a complex formation that is responsible for recruiting CD3 molecules and transmitting downstream signals.[30,41] The deletion of variable regions and extracellular parts of the constant regions can cause structural changes in the binding interfaces that interact with CD3 molecules.[42,43] These changes directly affect the stability of the TCR/CD3 complex and the number of CD3E proteins recruited to the cell surface.[44] The CD3 recruitment activities were markedly decreased when 40–87 amino acids (β4–β9) were removed from the constant region of TCRβ, suggesting the presence of a domain in the constant region of TCRβ that inhibits the binding of CD3 molecules and the TCRα in the absence of N-terminal domains (1–39 amino acids) of the TCRβ constant region. Interestingly, combinations of shorter TCRα and TCRβ constant regions exerted high CD3 recruitment activities, which raises the possibility of interaction modes of these TCR/CD3 complexes that differ from those involving native TCRs.

To date, most studies reporting the induction of T cells from iPSCs have been performed using full-length TCR-overexpressing iPSCs or T cell-derived iPSCs.[14,18–20,36,45–48] Our mini-TCRs achieved an efficient lineage commitment to the T cell lineage in the presence of a DLL4 protein. In T cell development *in vivo*, Notch signaling and pre-TCR signaling coordinate the proliferation of DN cells and their differentiation into DP cells.[41,49] Thus, in the differentiation culture condition, mini-TCRs, as well as full-length TCRs, might partially mimic the function of the pre-TCR, which consists of a pre-TCRα and a properly rearranged TCRβ, through the engagement of CD3 molecules on the cell surface.

On the other hand, we observed different expression patterns of CD4, CD8a, and CD8b between full-length TCRs and mini-TCRs when T cells were differentiated from both retroviral-transduced HSPCs and iPSCs transfected with transposon. A DN cell population was formed after the maturation process in mini-TCR-introduced iPSC-T cells, indicating that the developmental cues received from mini-TCRs were weaker than those from full-length TCRs (Figure 4). Moreover, the expression levels of CD3 and CD69 in mini-TCR-expressing Jurkat cells and iPSC-T cells were lower than full-length TCRs, while a variety of transduction efficiency was not observed. Although these phenomena are partially explained by structural differences, as discussed above, as well as the differing reactivity of OKT3 to TCR/CD3 complex, we aim to analyze cellular signaling and/or copy number in greater detail and to optimize the parameters of the cell culture conditions as part of a future study.

Recent reports to improve the efficiency of T cell induction and antitumor efficacy are worth considering for combination with the mini-TCR. Jing et al. reported efficient induction of iPSC-derived $CD8ab^+$ T cells by knocking down EZH1 and demonstrated superior *in vivo* antitumor efficacy due to the existence of memory-like T cells that gave rise to effector T cells.[50] EZH1 is a component of Polycomb Repressive Complex 2 as well as EZH2 and some available EZH1/2 inhibitors, such as lirametostat and valemetostat tosylate, can be

Figure 5. Induction of CTLs from mini-TCR-iPSCs

(A) Experimental scheme of T cell differentiation from mini-TCR-iPSCs. TCR genes were expressed with the piggyBac transposon method in iPSCs and the iPSCs were differentiated into CTLs. (B) Representative FACS plots of the CD34 and CD43 expression in iPSC-derived HSPCs. (C) Representative FACS plots of the CD45 and CD3E expression after T cell induction on DLL4 protein. (D) Representative FACS plots of CD4 and CD8α in CD3E + cells after T cell induction.

(E–H) Phenotypic analyses of CTLs after T cell maturation using the anti-CD3E antibody and dexamethasone. Representative FACS plots of CD45 and CD3E (E) and CD4 and CD8α (F), and CD8α and CD8β (G) in PI- cells are shown. (H) Estimated cell yields per HSPC. Cell numbers were calculated based on the cell numbers of seeded HSPCs and following proliferation rates (mean of technical duplicate). Representative of three independent experiments is shown. (I) Phenotypic analyses of memory T cell markers in CTLs after T cell maturation. The markers are indicated in the figure. Representative FACS plots of three independent experiments are shown.

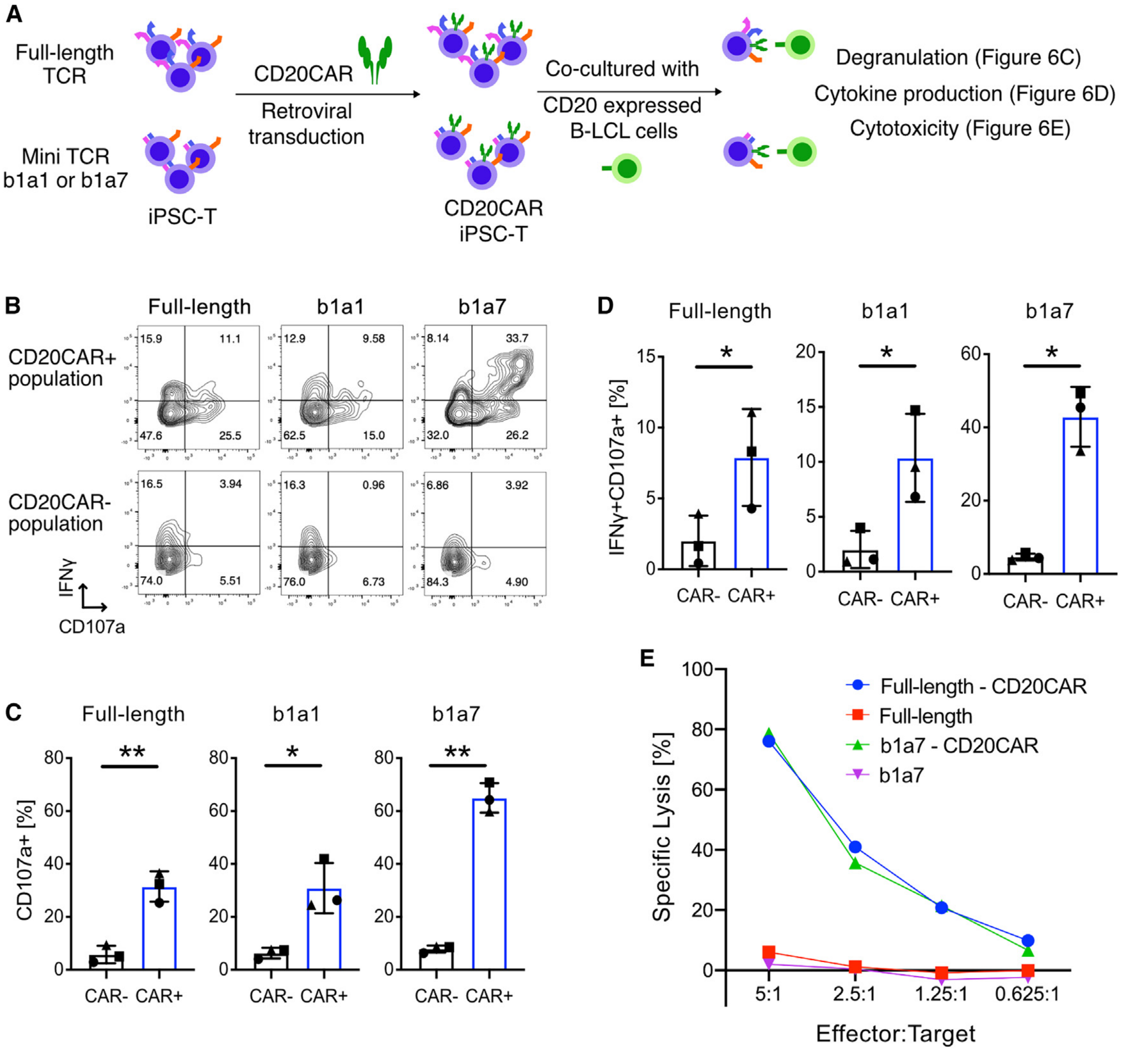

Figure 6. Effector functions of mini-TCR-iPS-CAR T cells

(A) Experimental scheme to evaluate effector functions of mini-TCR-iPSC-derived CTLs. The CTLs were transduced with CD20CAR retrovirus, and CD20+ B-LCL cells were employed as the target cells. (B) Representative FACS plots of CD107a and IFNγ in CTLs after co-culture with the target cells. (C) Percentages of CD107a+ cells in CD20CAR− and CD20CAR+ populations. Data are shown as mean ± SD of three independent experiments. (D) Percentages of CD107a+IFNγ+ cells in CD20CAR− and CD20CAR+ populations. Data are shown as mean ± SD of three independent experiments. Paired t tests, $^*p < 0.05$, $^{**}p < 0.01$, and $^{***}p < 0.001$. (E) *In vitro* cytotoxicity comparison between mini-TCR (b1a7)-iPS-CAR T cells and full-length TCR-iPS-CAR T cells. The specific lysis after a 3-h incubation with B-LCL cells is shown (mean of technical duplicate). The data represent two independent experiments.

added to the medium for feeder-free differentiation.[51] Stegen et al. reported a T cell induction method from iPSCs involving insertion of the CAR construct to the TRAC locus and achieved high antitumor efficacy.[52] This approach presents an alternative method for inducing functional CTLs from iPSCs. However, it is essential to note that the associated costs may limit its practicality. Establishing a master cell bank for each CAR construct contributes to the overall expenses involved in this approach. Mini-TCR iPS-T cells offer many specific advantages, such as accepting newly developed CAR constructs including co-expression of memory T cell-related cytokines such as interleukin (IL)-15[14,53] and dual-targeting CAR approaches.[54,55]

Among hundreds of CAR T-related clinical trials, only a few allogeneic peripheral-blood-derived CAR T cell products are under development, such as Allo-501A and Allo-715 by Allogene Therapeutics and CTX110 by CRISPR Therapeutics.[56,57] These technologies require complete deletion of the endogenous TCR activities; otherwise, the remaining TCRs might recognize the patients' cells and induce GvHD due to the presence of allogeneic T cells. In FT819, the first iPSC-derived CAR T cells in clinical development, the CAR gene targeting CD19 is inserted into the *TRAC* locus of T cell-derived iPSCs to prevent GvHD.[58,59] Our concept offers another safe strategy when starting from allogeneic non-T cell-derived iPSCs

because our mini-TCRs, which lack variable regions, would not recognize host cells. Although we have successfully demonstrated the loss of antigen-recognition capacity of the mini-TCR protein, one possible concern at the cellular level is a spontaneous rearrangement of endogenous TCR genes of host iPSCs during differentiation to express alloreactive TCRs. Several studies have reported that expression of endogenous TCR genes was not detectable in differentiated T cells when exogenous TCR genes were overexpressed in iPSCs.[18,36,48] However, it is noteworthy that endogenous TCR genes might still be expressed with a biased repertoire, and the TCR locus rearrangements might be higher in the mini-TCR compared to the full-length TCR, albeit at nearly negligible levels in NGS-based TCR gene expression analysis in this study. In the context of safety assessment for mini-TCR-iPSC-derived CTLs, it is essential to conduct future studies involving mixed leukocyte reactions and incorporate comprehensive manufacturing development processes. When aiming for complete avoidance of the risk of GvHD, disruption of the rearrangement-activating genes *RAG1* or *RAG2* would be worth considering.[36]

Finally, we present a comprehensive summary of the current state of iPS-T cell technology based on published research. Although culture conditions and iPSC lines exhibit variations, iPS-T cells commonly face challenges in maintaining memory T cell phenotypes, such as CCR7 and CD62L, and gene expression profiles during culture. Moreover, they demonstrate weaker cytokine and TCR signaling than clinically established primary CAR T cells. However, recent efforts to enhance iPS-T cell function have shown promising results. For instance, introducing optimized CAR constructs and membrane-bound IL-15 into iPS-T cells has led to comparable antitumor efficacy to primary CAR T cells in mouse tumor models.[14,15] Despite the extended duration required for T cell induction from iPSCs, leading to substantial costs, an off-the-shelf approach can mitigate expenses through large-scale manufacturing. It is worth noting that, while numerous engineered receptors, including CARs, have been reported to activate T cells, they typically contain variable domains derived from immunoglobulin or TCR.[45,46] Notably, mini-TCRs stand out as the first artificial receptors that lack variable regions while retaining the ability to activate T cells. This unique feature makes iPSCs bearing mini-TCRs an ideal platform for developing allogeneic immunotherapies.

In conclusion, the development of mini-TCRs and the potential use of iPSCs as a platform for allogeneic immunotherapies represent significant advancements in cellular therapies. This approach holds promise for future clinical applications and contributes to the continued progress in cutting-edge cellular therapies.

MATERIALS AND METHODS

Sequencing of full-length TCRA and TCRB

Full-length TCRα and TCRβ cDNA were amplified using the SMARTer RACE 5′/3′ Kit (Takara Bio) with T cell-derived iPSCs (TkT3v1-7,[18] University of Tokyo). Gene-specific primers for the constant regions for the RACE method were as follows: TCRA-5RACE, 5′-CAGCACTGTTGCTCTTGAAGTCCATAGACC-3′; TCRA-3RACE, 5′-ACCGATTTTGATTCTCAAACAAATGTGTCACAAAGTAAGG-3′; TCRB-5RACE, 5′-GGGTTCTGCCAGAAGGTGGCCGAGAC-3′; and TCRB-3RACE, 5′-TGGGAAGGAGGTGCACAGTGGGGTC-3′. Amplicons were sequenced using the standard Sanger sequencing method. The sequences of full-length TCRα and TCRβ cDNA are shown in Tables S3 and S4. The TCRβ was derived from the TRBC2 locus.

Vector construction

Vectors expressing TCRα or TCRβ

The constant regions were amplified by PCR from the full-length-TCRα or TCRβ cDNA samples and a Myc Tag or FLAG Tag was fused to the 3′ end of the constant regions via a GS (glycine serine) linker (sequence: GSGSGS) (designated as TCRA-Myc and TCRB-FLAG, respectively; Figure S1A). The PCR fragment was cloned into the pcDNA3.1(+) mammalian expression vector (Thermo Fisher Scientific) using the In-Fusion HD Cloning Kit (Clontech). The vector was then used as a template for inverse PCR to produce the TCR libraries with the constant region removed in a stepwise process. The sequences of TCRα and TCRβ of the generated TCR library are shown in Tables S5–S8. The sequences of the primers used for inverse PCR are shown in Table S9.

CD3 expression vectors

CD3E (NM_000733.3), CD3G (NM_000073.2), CD3D (NM_000732.4), CD3z (NM_198053.2), and EGFP were fused with T2A peptides (CD3E-T2A-CD3G-T2A-CD3D-T2A-CD3Z-T2A-EGFP) and subcloned into the pBApo-EF1α Pur mammalian expression vector (Takara Bio; pEF1α-CD3E-T2A-CD3G-T2A-CD3D-T2A-CD3Z-T2A-EGFP; Figure S1D).

TCRβ-T2A-TCRα mammalian expression vectors

To express equal number of TCRα and TCRβ proteins in each cell, the selected pairs of TCRα and TCRβ genes with signal peptides were fused with the GS linker (sequence: SGSG) and the T2A peptide (Figure 2B) and subcloned into the mammalian expression vector comprising EF1 promotor from pBApo-EF1α Pur vector (Takara Bio; Figure S2B), IRES-Kusabiraorange (hKO1, codon optimized for human use, derived from phKO1-S1 purchased from MBL), and BGH polyadenylation signal (designated as pEF1α-IRES-hKO1; Figure S1A).

Cell culture

293T cells (DSMZ), GP2-293 cells (Takara Bio), and 293FT cells (Invitrogen) were maintained in Dulbecco's modified Eagle's medium (DMEM, Nacalai Tesque) supplemented with 10% fetal bovine serum (FBS) and 1% L-glutamine-penicillin-streptomycin solution (PSG; Sigma) at 37°C in a 5% CO_2 atmosphere. Jurkat cells (DSMZ), B-LCL cells (RIKEN Cell Bank), and NALM6 cells (ATCC) were cultured in RPMI-1640 (Sigma) supplemented with 10% FBS and 1% PSG at 37°C in a 5% CO_2 atmosphere.

Flow cytometry

Cells were washed with phosphate-buffered saline (PBS) supplemented with 2% FBS and transferred into 96-well plates (2×10^5

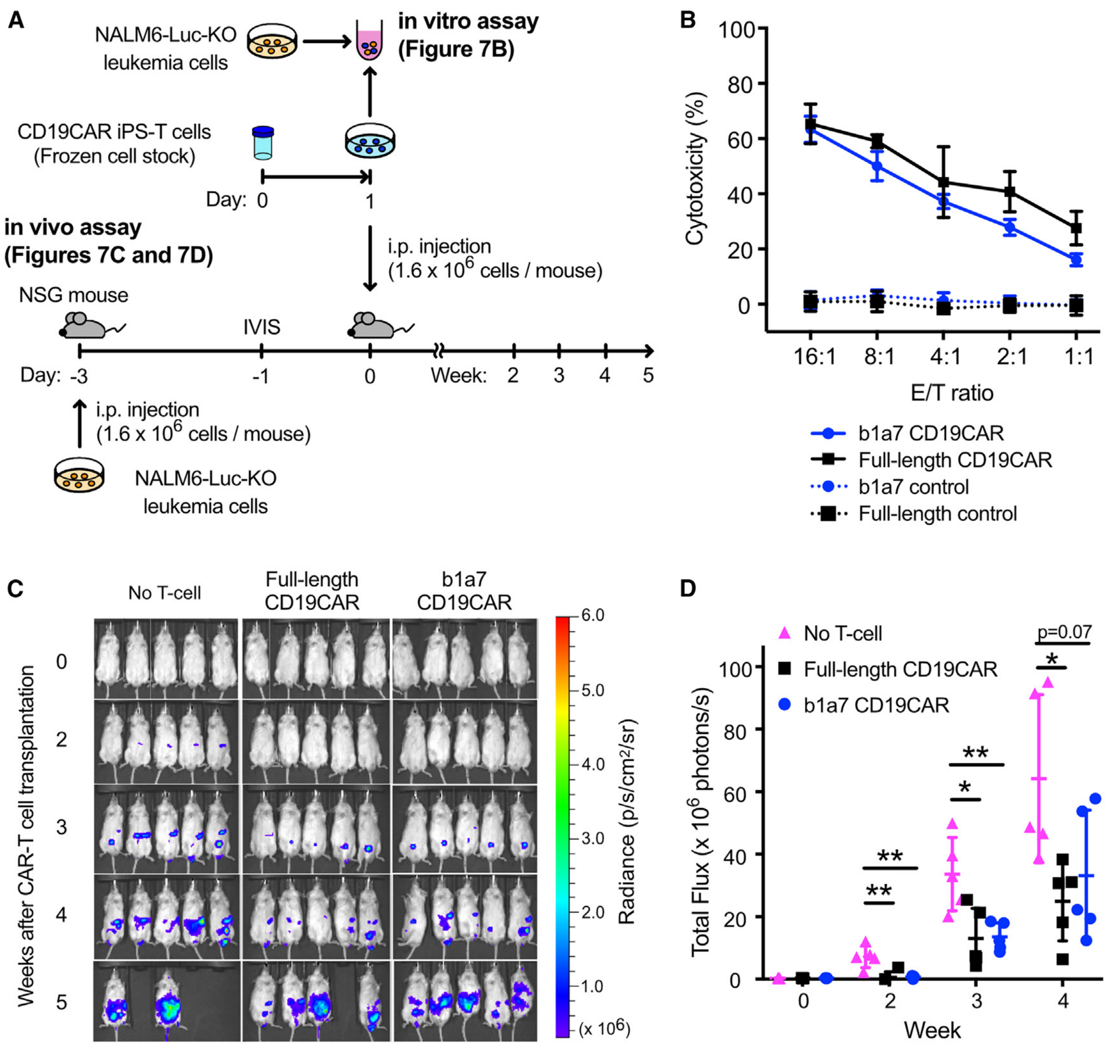

Figure 7. b1a7 CD19CAR T cells exhibit cytotoxic abilities both *in vivo* and *in vitro*

(A) Experimental scheme of the *in vitro* and *in vivo* cytotoxicity assays. (B) *In vitro* cytotoxicity of b1a7 CD19CAR and full-length TCR CD19CAR T cells against CD19+ NALM6-Luc-KO leukemia cells. Data are shown as mean ± SD of technical triplicates. (C) IVIS images at the indicated time points. (D) Amounts of NALM6-Luc-KO cells at the indicated time points. The scale bar represents the bioluminescence signal in radiance. Unpaired multiple t tests, *p < 0.05, **p < 0.01, and ***p < 0.001.

cells/well, 50 μL/well) or 15-mL tubes (1 × 10^6 cells/tube, 100 μL/tube). Fluorescent-labeled antibodies (2 μL each for 1 × 10^6 cells; detailed information is described in each section) were diluted with the buffer (total volume was 50 μL/sample) and transferred to the samples. After incubation at 4°C for 30 min, cells were washed with the buffer and resuspend in PBS supplemented with 2% FBS and DAPI (for SONY SH800 cell sorter, FUJIFILM Wako) or propidium iodide (for other machines, Thermo Fisher Scientific) at the volumes of 160 μL/well in 96-well plates or 500 μL/tube for 15-mL tubes. FCS files were analyzed on FlowJo v10 software.

Establishment of CD3-expressing 293T cells

293T cells (1.1 × 10^5 cells/well) were seeded into 12-well plates. Next day, the cells were washed with PBS to remove the antibiotics and 1 mL of DMEM supplemented with 10% FBS was added. The CD3 expression vector pEF1α-CD3E-T2A-CD3G-T2A-CD3D-T2A-CD3Z-T2A-EGFP (1 μg) and Lipofectamine 2000 (Thermo Fisher Scientific) were diluted in 50 μL of Opti-MEM (Thermo Fisher Scientific), respectively. After 5 min, the two diluted solutions were combined, incubated at room temperature for 5 min to form DNA-liposome complexes, and transferred into the 293T cells. EGFP+ cells were purified using FACS SH800 (SONY) and maintained to establish a stable transfectant (designated as CD3-OE-293T cells).

Establishment of TCR-KO Jurkat cells

Genome editing targeting TRAC and TRBC loci was performed to establish a double-KO mutant of Jurkat cells (designated as TCR-KO Jurkat cells) using the Alt-R CRISPR-Cas9 System (Integrated DNA

Technologies). TCRαTCRβ double negative cells were purified by FACS on SONY SH800. Disruption of TCRA and TCRB genes was confirmed by single transfection with the full-length TCRα or full-length TCRβ expression vector and subsequent FACS analysis using anti-TCRαβ and anti-CD3E (approximately 99% of the cells were negative for TCRαβ and CD3E). The target genomic sequences were as follows: *TRAC*,[60] 5′-GAGAATCAAAATCGGTGAAT-3′; *TRBC1/TRBC2*,[61] 5′-CAAACACAGCGACCTCGGGT-3′. Anti-TCRαβ-APC (clone IP26, BioLegend) and anti-CD3E-PE-Cy7 (clone UCHT1, BioLegend) were used for FACS analysis on a SONY SH800.

Intracellular staining of TCR proteins

To validate the protein expression from TCRA-Myc or TCRB-FLAG vectors, CD3-OE-293T cells were seeded in 12-well plates at 1.1 × 10^5 cells/well. The next day, TCRA-Myc or TCRB-FLAG (0.8 μg) and pEF1α-IRES-hKO1 vectors (0.2 μg, as a transfection marker) (total 1.0 μg) were mixed in 50 μL of Opti-MEM and cotransfected into CD3-OE-293T cells as described in the "establishment of CD3-expressing 293T cells" section. Two days after transfection, cells were harvested with trypsin (0.05%)-EDTA phenol red (Gibco) and washed with FACS buffer (PBS [Nacalai Tesque] + 2% FBS [Access Cell Culture] + 1 mM EDTA [Invitrogen]). Then, the cells were fixed with Fixation Buffer (BioLegend) and permeabilized with Perm Wash Buffer (BioLegend). The cells were stained with anti-Myc Tag-Alexa Fluor 647 (clone 9B11, Cell Signaling) or anti-DYKDDDDK (FLAG) Tag-Alexa Fluor 647 (clone L5, BioLegend). The stained cells were suspended in Cell Staining Buffer (BioLegend) and analyzed using BD LSRII (BD Bioscience).

Detection of CD3E protein on CD3-OE-293T cells

To detect cell surface localization of CD3E protein on CD3-OE-293T cells, the following two combinations of vectors were transfected as described in the "intracellular staining of TCR proteins" section.

(1) TCRA-Myc (0.4 μg), TCRB-FLAG (0.4 μg), and pEF1α-IRES-hKO1 vectors (0.2 μg, as a transfection marker) (total 1.0 μg) (Figures 1 and S1)
(2) TCRβ-T2A-TCRα-pEF1α-IRES-hKO1 vectors (1.0 μg) (Figure 2)

Two to 3 days later, cells were harvested by trypsinization, stained with anti-CD3E-APC (clone UCHT1, BioLegend), and analyzed using a BD LSRII (BD Bioscience).

Tetramer binding assay

TCRβ-T2A-TCRα-pEF1α-IRES-hKO1 vectors (1.0 μg) including full-length TCRs[35] were transfected into CD3-OE-293T cells as described in the "intracellular staining of TCR proteins" section. Two to 3 days later, cells were harvested by trypsinization, stained with T-Select HLA-A*24:02 modified WT1 tetramer-CYTWNQMNL-APC (MBL) and HLA-A*24:02 control tetramer-AYAAAAAAL-APC (MBL) as described in the "flow cytometry" section and analyzed using a BD LSRII (BD Bioscience).

Detection of CD3 proteins on TCR-KO Jurkat cells

To detect cell surface expression of CD3E and CD3D proteins on TCR-KO Jurkat cells, TCRβ-T2A-TCRα-pEF1α-IRES-hKO1 vectors (2 μg) were transfected into TCR-KO Jurkat cells (1 × 10^6 cells/sample, 100 μL/well) by using the 4D-Nucleofector electroporator (Lonza, program: CL-120) and SE Cell Line 4D-Nucleofector X Kit L (Lonza) according to the manufacturer's instruction.

Two days later, transfected cells were harvested and stained with anti-CD3E-PE-Cy7 (clone UCHT1, BioLegend) and anti-CD3D-APC (clone 7D6, Invitrogen). Biotinylated anti-CD3E antibody OKT3 (BioLegend) was diluted to 200 μg/mL and serial dilution was performed (1:5). Diluted antibody solution (100 μL/well) was transferred to a 96-well plate and mixed with equal amount of TCR-transfected TCR-KO Jurkat cells (1 × 10^5 cells/sample, 100 μL/well). After incubation (4°C, 30 min), cells were washed twice and stained with Alexa 647-labeled streptavidin (BioLegend, diluted in 1:400, 50 μL/sample). Samples were prepared as described in the "flow cytometry" section and analyzed using a BD LSRII (BD Bioscience).

CD3 stimulation of TCR-KO Jurkat cells

Transfection into TCR-KO Jurkat cells was performed as described in the "detection of CD3 proteins on TCR-KO Jurkat cells" section and incubated overnight (day 0). Ultra-LEAF purified anti-human CD3 (clone OKT3, BioLegend) was plated into 96-well plates (10 μg/mL in PBS, 100 μL/well) and incubated overnight (day 0). After washing the CD3-coated plate with PBS, transfected TCR-KO Jurkat cells or parental Jurkat cells were seeded at the cell density of 1–2 × 10^5 cells/well and incubated overnight (day 1). Jurkat cells were stained with anti-CD69-Alexa Fluor 647 (clone FN50, BioLegend) and analyzed using a BD LSRII (BD Bioscience).

Retroviral production

Full-length and selected pairs of TCRβ and TCRα (b1a1 and b1a7) described in the section "TCRβ-T2A-TCRα mammalian expression vectors" were transferred into pMYs-IRES-GFP retroviral vector (pMYs-IG, Cell Biolabs). The CD19CAR sequence was obtained from an application for a US patent (US2014-0271635). The synthesized CD19CAR cDNA was subcloned into pMYs-IG. The CD20CAR cDNA[16,62] was subcloned into the pGCDNsam vector.[63]

For viral production, GP2-293 cells (Takara Bio) were seeded onto poly-L-lysine 5× (Sigma-Aldrich)-coated 10-cm dishes (5 × 10^6 cells/dish) and incubated overnight. pMYs-IG vectors (2 μg) and VSV-G vector (Takara Bio, 2 μg) were diluted in Opti-MEM (200 μL) and combined with Lipofectamine 2000 (Thermo Fisher Scientific; 12 μL were diluted in Opti-MEM 200 μL). After 5 min of incubation at room temperature, they were transfected into the GP2-293 cells and incubated for 2 days. The harvested supernatant (10 mL/dish) was filtered and mixed with a Retro-X Concentrator (Takara Bio, 3.3 mL) and incubated overnight at 4°C. After centrifugation (at 1,500 × *g*, 45 min, 4°C), the pellet was resuspended in alpha-MEM (300 μL). The titers were determined by infection with

the serial diluted viruses to Jurkat cells and FACS analyses. Aliquots were stored in a −80°C deep freezer until further use.

Lentiviral production and establishment of NALM6-Luc-KO cells

The GFP gene in CMV-Luciferase-EF1a-copGFP-T2A-Puro Lentivector (System Biosciences) was replaced with hKO1 amplified from the phKO1-S1 vector (MBL). Lentivirus was produced as previously described using 293FT cells.[35] The concentrated lentivirus was titrated with Jurkat cells and infected to NALM6 cells (MOI = 6) by the spin-infection method in 24-well plates (32°C, 2000 × *g*, 2 h). hKO1+ NALM6 cells were sorted on BD FACSAria II and expanded.

T cell induction from iPSCs

Maintenance of feeder-free iPSCs

Myeloid-derived iPSCs (Ff-WJs513, Ff-WJs524, Ff-WJs527, and FfI01s04; all clones were established from cord-blood cells in CiRA, Kyoto University[64,65]) and T cell-derived iPSCs (TkT3V1-7, provided by the University of Tokyo) were maintained in StemFit AK03N (Ajinomoto) on iMatrix-511 (Matrixome) as previously reported.[37]

Establishment of TCR-expressing iPSCs

Full-length GPC3-specific TCRs (provided by Shinobi Therapeutics) and selected pairs of TCRβ and TCRα (b1a1 and b1a7) described in section "TCRβ-T2A-TCRα mammalian expression vectors" were transferred into piggyBac transfer vector (kindly provided by Dr. Yozo Nakazawa, Shinshu University). iPSCs (FfI01s04, 4 × 10^6 cells/sample) were washed with MaxCyte buffer (HyClone, EPB1) three times and resuspended in MaxCyte buffer (4 × 10^6 cells per 100 μL) in an OC-100 cuvette. Then, the transfer and transposase vectors (16 μg each) were electroporated into the cells using MaxCyte STX (MaxCyte) in the Optimization 8 condition. Following the electroporation, the cells were immediately placed in culture medium and incubated at 37°C and 5% CO_2 for 30 min. Then, the cells were transferred to iMatrix-511 coated six-well plates with StemFit AK03. After 5–7 days, CD19-positive cells were sorted by FACS (Figure S5).

Generation of HSPCs from iPSCs

HSPCs were induced by using the embryoid body (EB) method as previously reported.[14] Briefly, iPSCs dissociated into single cells using 0.5× TrypLE Select (Thermo Fisher Scientific) and 0.75 mM EDTA were resuspended in StemFit AK02N supplemented with 10 μM Y-27632 (Nacalai Tesque) and 10 μM CHIR99021 (Tocris Bioscience) and cultured in six-well ultra-low attachment plates (Corning) for 24 h at a concentration of 0.2–0.3 × 10^6 cells/well in a 5% O_2 incubator (day 0). The next day, the cells were harvested and resuspended in 2 mL of StemPro-34 (Thermo Fisher Scientific) supplemented with 0.2× PSG, 1× GlutaMAX (Thermo Fisher Scientific), 50 μg/mL ascorbic acid-2-phosphate (PAA, FUJIFILM Wako), 45 mM monothioglycerol (FUJIFILM Wako), and 1× insulin-transferrin-selenium solution (Thermo Fisher Scientific) (hereafter referred to as EB basal medium), 50 ng/mL recombinant human (rh) BMP-4 (Miltenyi Biotec), 50 ng/mL rhVEGF (FUJIFILM Wako), and 50 ng/mL bFGF (FUJIFILM Wako) per well (day 1, 5% O_2, 5% CO_2). After 24 h, 6 μM SB431542 (FUJIFILM Wako) was added (day 2, 5% O_2, 5% CO_2). After 2 days, the differentiating EBs were collected, washed, and resuspended in 2 mL of EB basal medium supplemented with 50 ng/mL rhVEGF, 50 ng/mL rhbFGF, and 50 ng/mL rhSCF (R&D Systems) per well and cultured for 2 days (day 4, 5% O_2, 5% CO_2). After 2 days, the EBs were cultured in 2 mL of EB basal medium supplemented with 50 ng/mL rhVEGF, 50 ng/mL rhbFGF, 50 ng/mL rhSCF (R&D Systems), 30 ng/mL rhTPO (FUJIFILM Wako), and 10 ng/mL rhFLT3L (FUJIFILM Wako) (day 6, 5% CO_2). The medium was changed every 2–3 days until day 13. Harvested cells were resuspended in TC-Protector cell freezing medium (Iwai) and stored in a −150°C deep freezer.

Transduction of TCR genes and T cell induction

HSPCs were differentiated into T cells as previously described.[14] Briefly, thawed EB-derived HSPCs (from Ff-WJs513, Ff-WJs524, and Ff-WJs527 cells) were incubated in EB medium supplemented with 50 ng/mL VEGF-165A, 50 ng/mL rhbFGF, 50 ng/mL rhSCF, 50 ng/mL rhTPO, and 10 ng/mL rhFLT3L (day −2). The next day, cells were seeded into 48-well plates coated with RetroNectin (Takara Bio, 100 μg/mL) at a cell density of 2–4 × 10^4 cells/well. Titrated retrovirus was added to the cell suspension (day −1, MOI = 20). The next day, cells were seeded into plates coated with RetroNectin (5 μg/mL) and DLL4 protein (5 μg/mL) and incubated in alpha-MEM supplemented with 50 ng/mL rhSCF, 50 ng/mL rhIL-7 (FUJIFILM Wako), 50 ng/mL rhFLT3L, 100 ng/mL rhTPO, 30 nM rhSDF-1α (FUJIFILM Wako), 15 μM SB203580 (FUJIFILM Wako), 50 μg/mL PAA, 15% FBS, and 1% PSG (day 0). The medium was changed every 2–3 days, and cells were reseeded into fresh RetroNectin- and DLL4-coated 48-well plates every 7 days until day 21. Anti-CD3-BV510 (BioLegend) and anti-CD45-APC-Cy7 (BioLegend) were used for FACS analysis.

Maturation of T cell progenitors to CD8α+CD8β+ CTLs

T cells induced on DLL4 proteins were seeded into 48-well plates at a density of 1–10 × 10^4 cells/well and stimulated with OKT3 anti-CD3 antibody (eBioscience, 500 ng/mL) in alpha-MEM supplemented with 15% FBS, 50 μg/mL PAA, 1% PSG, 10 ng/mL rhIL-7, and 10 nM dexamethasone (DEXART, Fuji Pharma) for 3 days (days 21–24).[14,36] OKT3 and dexamethasone were removed by changing the medium (alpha-MEM supplemented with 15% FBS, 50 μg/mL PAA, 1% PSG, and 10 ng/mL rhIL-7), and cells were incubated for another 7 days (days 24–31). Anti-CD8β-PE-Cy7, anti-CD8α-APC, anti-CD45-APC-Cy7, anti-CD4-BV421, and anti-CD3-BV510 antibodies were used for FACS analysis.

T cell expansion

CTLs were co-cultured with irradiated PBMCs (40 Gy) as feeder cells in alpha-MEM supplemented with 10% FBS, 1% PSG, IL-7 (5 ng/mL), IL-15 (5 ng/mL), pan-caspase inhibitor Z-VAD-FMK (10 μM, R&D Systems), PAA (50 μg/mL), ITS-G, and phytohemagglutinin (PHA; 2 μg/mL, FUJIFILM Wako) for 14 days. Feeder-free expansion was performed based on the previous report.[14] The medium was replenished every 2–3 days, and cell passaging was performed every 2–6 days.

TCR gene expression analysis

The NGS to evaluate expression of endogenous and transduced TCR genes was performed as previously described.[36] Total RNA was isolated from CTLs after expansion (see the section "T cell expansion") using RNeasy micro kit (QIAGEN) following the manufacturer's instructions. After reverse transcription, the cDNA was amplified by using adopter primer at the 5′ and the reverse primers to constant domains of TCRA and TCRB. After the sequencing of the amplified CDR3 regions from both TCRA and TCRB genes on a next-generation sequencer, entire sequenced reads (number of reads are depicted as "Total reads" in Tables S1 and S2) were analyzed using the repertoire analysis software Repertoire Genesis to assign TRV and TRJ alleles to queries and then generate CDR3 sequences and aggregate their combination patterns ("Total assigned reads" in Tables S1 and S2). In-frame sequences for which the CDR3 region can be translated to amino acid sequences were analyzed (Figures 4J and S4B; "Total in-frame reads" in Tables S1 and S2).

Retroviral transduction into iPSC-derived T cells

Anti-CD19 CAR retrovirus was transduced into iPS-T cells. A 96-well plate was coated with RetroNectin (Takara Bio) for 2 h at room temperature or overnight at 4°C. Two days after CD3/CD28 stimulation, iPS-T cells were transduced with concentrated retroviral supernatants by centrifugation on a RetroNectin-coated plate (MOI = 20). Cells were cultured for 11 days from transduction (total 13 days). Transduction was confirmed with flow cytometry with protein-L (GenScript) and streptavadin-APC (BioLegend). The cryopreserved cell stocks were prepared as outlined in "generation of HSPCs from iPSCs" section (Figure S7A).

CD107a and cytokine detection

For detection of CD107a and IFNγ expression of iPSC-T cells, T cells were co-cultured with B-LCLs cells at a 1:1 ratio in a 96-well plate for 5 h in 100 μL of RPMI-1640 supplemented with glutamine, 1× Monensin (BioLegend), 10% FBS, and anti-CD107a-APC antibody (BioLegend, Clone H4A3, 1 μL). Then the cell surface was stained with anti-CD20-PE-Cy7 (BD Pharmingen) and anti-EGFR-PE (BioLegend). Samples were washed and fixed with Fixation Buffer (BioLegend) for 20 min. Samples were washed twice with Permeabilization Wash Buffer (BioLegend) and were stained with IFNγ-APC-Cy7 (BioLegend) for 20 min. The washed and resuspended cells were analyzed using BD FACSAria II.

In vitro cytotoxicity assay

A nonradioactive cellular cytotoxicity assay kit (Techno-Suzuta, Nagasaki, Japan) was used for CD20CAR T cells. Briefly, target B-LCLs were pulsed with BM-HT reagent at 37°C for 15 min, washed three times, and seeded into 96-well plates at a density of 5,000 cells/well. Effector cells were added to the wells at effector-to-target (E:T) ratios ranging from 5:1 to 0.625:1 and were co-cultured for 3 h with α-MEM supplemented with 15% FBS and 1% PSG at 37°C with 5% CO_2. Then, 40 μL of the co-culture supernatant was collected and mixed with 160 μL of Eu solution, and time-resolved fluorescence was measured using a NIVO plate reader (PerkinElmer, Waltham, MA). *In vitro* cytotoxicity of the CD19CAR T cells were examined using the standard 51Cr release method. Briefly, target NALM6-Luc-KO cells were loaded with 1.85 MBq 51Cr for 1 h in α-MEM supplemented with 15% FBS and 1% PSG. Target cells (5,000 cells/well) were co-cultured with effector iPSC-derived CAR T cells for 5 h in the medium with various effector-to-target (E:T) ratios ranging from 16:1 to 1:1. Supernatants were harvested and the released 51Cr was measured using a beta counter (PerkinElmer).

Mouse leukemia model

Male NSG mice (11 weeks old; Charles River Laboratories Japan) were housed under controlled humidity and light/dark cycle in a specific pathogen-free facility. All animal experiments were performed in accordance with the Animal Review Board of Kyoto University. A total of 1×10^6 NALM6-Luc-KO cells were intraperitoneally (i.p.) injected to establish a mouse leukemia model (day −3). From day −1, the tumor burden was monitored weekly by *in vivo* bioluminescence imaging using IVIS SPECTRUM and Living Image 4.7.3 software (PerkinElmer). CAR T cells (1.6×10^6 cells/mouse) were i.p. injected into mice grouped according to the luminescence on day 0. From day −3, body weight was monitored daily, and 20% weight loss compared to that on day −3 was used as the humane endpoint for sacrifice.

DATA AND CODE AVAILABILITY

The sequences identified and designed during this study are available from supplementary tables or from the corresponding author upon reasonable request.

SUPPLEMENTAL INFORMATION

Supplemental information can be found online at https://doi.org/10.1016/j.omtm.2023.101109.

ACKNOWLEDGMENTS

We thank T. Ueda, S. Iriguchi, Y. Kawai, Y. Konishi, K. Kondo, K. Nakazono, K. Sasaki, and K. Tomizuka for critical discussions; K. Takaki and A. Fukui for technical assistance; M. Osawa for designing the piggyBac vector; and the CiRA FACS core lab and animal facility for supporting the experiments. We thank Dr. Yozo Nakazawa for providing the piggyBac transposon vector and for his advice. We also thank Dr. P. Karagiannis for editing the manuscript. The study was conducted in accordance with the Declaration of Helsinki and was approved by the Institutional Ethics Board of Kyoto University.

AUTHOR CONTRIBUTIONS

S.-i.T. conceived the study, designed the experiments, performed the research, analyzed the data, and wrote the manuscript. B.W., S.H., S.N., and K.F. designed the experiments, performed the research, analyzed the data, and wrote the manuscript. K.N., S.C., and Y.K. designed the experiments and performed the research. A.M. provided critical methods. S. Kamibayashi performed the experiments. A.K. and H.N. interpreted the data. S. Kaneko edited the manuscript and supervised the study.

DECLARATION OF INTERESTS

This work was supported by a collaborative research grant from Kirin Holdings. S.-i.T., S.H., K.F., A.K., S.N., and S. Kaneko declare a patent titled "WO2020138371 MODIFIED TCR AND PRODUCTION METHOD THEREFOR" related to this work. S.-i.T., S.H., K.F., S.C., Y.K., A.K., and H.N. are employed by Kirin Holdings Co., Ltd. K.N. and B.W. are an employee and a part-time employee of Shinobi Therapeutics, Inc., respectively. S. Kaneko is a founder, shareholder, and chief scientific officer at Shinobi Therapeutics, Inc., and received research funding from Kirin Holdings Co., Ltd.; Takeda Pharmaceutical Co., Ltd.; Shinobi Therapeutics, Inc.; Astellas Co., Ltd.; Terumo Co., Ltd.; and Tosoh Co., Ltd.

REFERENCES

1. Baskar, R., Lee, K.A., Yeo, R., Yeoh, K.-W., Baskar, R., and Phil, M. (2012). Cancer and Radiation Therapy: Current Advances and Future Directions. Int. J. Med. Sci. *9*, 193–199.
2. Rosenberg, S.A., and Restifo, N.P. (2015). Adoptive cell transfer as personalized immunotherapy for human cancer. Science *348*, 62–68.
3. Park, J.H., Geyer, M.B., and Brentjens, R.J. (2016). CD19-targeted CAR T-cell therapeutics for hematologic malignancies: Interpreting clinical outcomes to date. Blood *127*, 3312–3320.
4. Martino, M., Alati, C., Canale, F.A., Musuraca, G., Martinelli, G., and Cerchione, C. (2021). A Review of Clinical Outcomes of CAR T-Cell Therapies for B-Acute Lymphoblastic Leukemia. Int. J. Mol. Sci. *22*, 2150.
5. Tully, S., Feng, Z., Grindrod, K., McFarlane, T., Chan, K.K.W., and Wong, W.W.L. (2019). Impact of Increasing Wait Times on Overall Mortality of Chimeric Antigen Receptor T-Cell Therapy in Large B-Cell Lymphoma: A Discrete Event Simulation Model. JCO Clin. Cancer Inform. *3*, 1–9.
6. Caldwell, K.J., Gottschalk, S., and Talleur, A.C. (2020). Allogeneic CAR Cell Therapy—More Than a Pipe Dream. Front. Immunol. *11*, 618427.
7. Sadelain, M., Rivière, I., and Riddell, S. (2017). Therapeutic T cell engineering. Nature *545*, 423–431.
8. Vormittag, P., Gunn, R., Ghorashian, S., and Veraitch, F.S. (2018). A guide to manufacturing CAR T cell therapies. Curr. Opin. Biotechnol. *53*, 164–181.
9. Iriguchi, S., and Kaneko, S. (2019). Toward the development of true "off-the-shelf" synthetic T-cell immunotherapy. Cancer Sci. *110*, 16–22.
10. Cutmore, L.C., and Marshall, J.F. (2021). Current Perspectives on the Use of off the Shelf CAR-T/NK Cells for the Treatment of Cancer. Cancers *13*, 1926.
11. Li, Y., Hermanson, D.L., Moriarity, B.S., and Kaufman, D.S. (2018). Human iPSC-derived Natural Killer Cells Engineered with Chimeric Antigen Receptors Enhance Anti-Tumor Activity. Cell Stem Cell *23*, 181–192.e5.
12. Arias, J., Yu, J., Varshney, M., Inzunza, J., and Nalvarte, I. (2021). Hematopoietic stem cell- and induced pluripotent stem cell-derived CAR-NK cells as reliable cell-based therapy solutions. Stem Cells Transl. Med. *10*, 987–995.
13. Sachamitr, P., Hackett, S., and Fairchild, P.J. (2014). Induced pluripotent stem cells: challenges and opportunities for cancer immunotherapy. Front. Immunol. *5*, 176–179.
14. Iriguchi, S., Yasui, Y., Kawai, Y., Arima, S., Kunitomo, M., Sato, T., Ueda, T., Minagawa, A., Mishima, Y., Yanagawa, N., et al. (2021). A clinically applicable and scalable method to regenerate T-cells from iPSCs for off-the-shelf T-cell immunotherapy. Nat. Commun. *12*, 430–515.
15. Ueda, T., Shiina, S., Iriguchi, S., Terakura, S., Kawai, Y., Kabai, R., Sakamoto, S., Watanabe, A., Ohara, K., Wang, B., et al. (2023). Optimization of the proliferation and persistency of CAR T cells derived from human induced pluripotent stem cells. Nat. Biomed. Eng. *7*, 24–37.
16. Wang, B., Iriguchi, S., Waseda, M., Ueda, N., Ueda, T., Xu, H., Minagawa, A., Ishikawa, A., Yano, H., Ishi, T., et al. (2021). Generation of hypoimmunogenic T cells from genetically engineered allogeneic human induced pluripotent stem cells. Nat. Biomed. Eng. *5*, 429–440.
17. Dustin, M.L. (2014). The immunological synapse. Cancer Immunol. Res. *2*, 1023–1033.
18. Nishimura, T., Kaneko, S., Kawana-Tachikawa, A., Tajima, Y., Goto, H., Zhu, D., Nakayama-Hosoya, K., Iriguchi, S., Uemura, Y., Shimizu, T., et al. (2013). Generation of rejuvenated antigen-specific T cells by reprogramming to pluripotency and redifferentiation. Cell Stem Cell *12*, 114–126.
19. Maeda, T., Nagano, S., Ichise, H., Kataoka, K., Yamada, D., Ogawa, S., Koseki, H., Kitawaki, T., Kadowaki, N., Takaori-Kondo, A., et al. (2016). Regeneration of CD8αβ T cells from T-cell-derived iPSC imparts potent tumor antigen-specific cytotoxicity. Cancer Res. *76*, 6839–6850.
20. Maeda, T., Nagano, S., Kashima, S., Terada, K., Agata, Y., Ichise, H., Ohtaka, M., Nakanishi, M., Fujiki, F., Sugiyama, H., et al. (2020). Regeneration of Tumor-Antigen-Specific Cytotoxic T Lymphocytes from iPSCs Transduced with Exogenous TCR Genes. Mol. Ther. Methods Clin. Dev. *19*, 250–260.
21. Ochi, T., Nakatsugawa, M., Chamoto, K., Tanaka, S., Yamashita, Y., Guo, T., Fujiwara, H., Yasukawa, M., Butler, M.O., and Hirano, N. (2015). Optimization of T-cell reactivity by exploiting TCR chain centricity for the purpose of safe and effective antitumor TCR gene therapy. Cancer Immunol. Res. *3*, 1070–1081.
22. Rosati, E., Dowds, C.M., Liaskou, E., Henriksen, E.K.K., Karlsen, T.H., and Franke, A. (2017). Overview of methodologies for T-cell receptor repertoire analysis. BMC Biotechnol. *171 17*, 61.
23. Smith-Garvin, J.E., Koretzky, G.A., and Jordan, M.S. (2009). T Cell Activation. Annu. Rev. Immunol. *27*, 591–619.
24. Kuhns, M.S., and Davis, M.M. (2007). Disruption of Extracellular Interactions Impairs T Cell Receptor-CD3 Complex Stability and Signaling. Immunity *26*, 357–369.
25. Natarajan, A., Nadarajah, V., Felsovalyi, K., Wang, W., Jeyachandran, V.R., Wasson, R.A., Cardozo, T., Bracken, C., and Krogsgaard, M. (2016). Structural Model of the Extracellular Assembly of the TCR-CD3 Complex. Cell Rep. *14*, 2833–2845.
26. Krshnan, L., Park, S., Im, W., Call, M.J., and Call, M.E. (2016). A conserved αβ transmembrane interface forms the core of a compact T-cell receptor-CD3 structure within the membrane. Proc. Natl. Acad. Sci. USA *113*, E6649–E6658.
27. Dong, D., Zheng, L., Lin, J., Zhang, B., Zhu, Y., Li, N., Xie, S., Wang, Y., Gao, N., and Huang, Z. (2019). Structural basis of assembly of the human T cell receptor–CD3 complex. Nature *573*, 546–552.
28. Newell, E.W., Ely, L.K., Kruse, A.C., Reay, P.A., Rodriguez, S.N., Lin, A.E., Kuhns, M.S., Garcia, K.C., and Davis, M.M. (2011). Structural Basis of Specificity and Cross-Reactivity in T Cell Receptors Specific for Cytochrome c–I-Ek. J. Immunol. *186*, 5823–5832.
29. Okamoto, S., Amaishi, Y., Goto, Y., Ikeda, H., Fujiwara, H., Kuzushima, K., Yasukawa, M., Shiku, H., and Mineno, J. (2012). A promising vector for TCR gene therapy: Differential effect of siRNA, 2A peptide, and disulfide bond on the introduced TCR expression. Mol. Ther. Nucleic Acids *1*, e63.
30. Pageon, S.V., Tabarin, T., Yamamoto, Y., Ma, Y., Nicovich, P.R., Bridgeman, J.S., Cohnen, A., Benzing, C., Gao, Y., Crowther, M.D., et al. (2016). Functional role of T-cell receptor nanoclusters in signal initiation and antigen discrimination. Proc. Natl. Acad. Sci. USA *113*, E5454–E5463.
31. Radtke, F., MacDonald, H.R., and Tacchini-Cottier, F. (2013). Regulation of innate and adaptive immunity by Notch. Nat. Rev. Immunol. *13*, 427–437.
32. Anderson, G., and Takahama, Y. (2012). Thymic epithelial cells: Working class heroes for T cell development and repertoire selection. Trends Immunol. *33*, 256–263.
33. Kondo, K., Ohigashi, I., and Takahama, Y. (2019). Thymus machinery for T-cell selection. Int. Immunol. *31*, 119–125.
34. Kustikova, O.S., Wahlers, A., Kühlcke, K., Stähle, B., Zander, A.R., Baum, C., and Fehse, B. (2003). Dose finding with retroviral vectors: correlation of retroviral vector copy numbers in single cells with gene transfer efficiency in a cell population. Blood *102*, 3934–3937.

35. Mittelstadt, P.R., Monteiro, J.P., and Ashwell, J.D. (2012). Thymocyte responsiveness to endogenous glucocorticoids is required for immunological fitness. J. Clin. Invest. *122*, 2384–2394.

36. Minagawa, A., Yoshikawa, T., Yasukawa, M., Hotta, A., Kunitomo, M., Iriguchi, S., Takiguchi, M., Kassai, Y., Imai, E., Yasui, Y., et al. (2018). Enhancing T Cell Receptor Stability in Rejuvenated iPSC-Derived T Cells Improves Their Use in Cancer Immunotherapy. Cell Stem Cell *23*, 850–858.e4.

37. Okita, K., Matsumura, Y., Sato, Y., Okada, A., Morizane, A., Okamoto, S., Hong, H., Nakagawa, M., Tanabe, K., Tezuka, K.I., et al. (2011). A more efficient method to generate integration-free human iPS cells. Nat. Methods *8*, 409–412.

38. Kawai, Y., Kawana-Tachikawa, A., Kitayama, S., Ueda, T., Miki, S., Watanabe, A., and Kaneko, S. (2021). Generation of highly proliferative rejuvenated cytotoxic T cell clones through pluripotency reprogramming for adoptive immunotherapy. Mol. Ther. *29*, 3027–3041.

39. Gattinoni, L., Lugli, E., Ji, Y., Pos, Z., Paulos, C.M., Quigley, M.F., Almeida, J.R., Gostick, E., Yu, Z., Carpenito, C., et al. (2011). A human memory T cell subset with stem cell-like properties. Nat. Med. *17*, 1290–1297.

40. Mariuzza, R.A., Agnihotri, P., and Orban, J. (2020). The structural basis of T-cell receptor (TCR) activation: An enduring enigma. J. Biol. Chem. *295*, 914–925.

41. Courtney, A.H., Lo, W.L., and Weiss, A. (2018). TCR Signaling: Mechanisms of Initiation and Propagation. Trends Biochem. Sci. *43*, 108–123.

42. Kuhns, M.S., Davis, M.M., and Garcia, K.C. (2006). Deconstructing the form and function of the TCR/CD3 complex. Immunity *24*, 133–139.

43. Birnbaum, M.E., Berry, R., Hsiao, Y.S., Chen, Z., Shingu-Vazquez, M.A., Yu, X., Waghray, D., Fischer, S., McCluskey, J., Rossjohn, J., et al. (2014). Molecular architecture of the αβ T cell receptor-CD3 complex. Proc. Natl. Acad. Sci. USA *111*, 17576–17581.

44. Schamel, W.W., Alarcon, B., and Minguet, S. (2019). The TCR is an allosterically regulated macromolecular machinery changing its conformation while working. Immunol. Rev. *291*, 8–25.

45. Rafiq, S., Hackett, C.S., and Brentjens, R.J. (2020). Engineering strategies to overcome the current roadblocks in CAR T cell therapy. Nat. Rev. Clin. Oncol. *17*, 147–167.

46. Oh, J., Warshaviak, D.T., Mkrtichyan, M., Munguia, M.L., Lin, A., Chai, F., Pigott, C., Kang, J., Gallo, M., and Kamb, A. (2019). Single variable domains from the T cell receptor β chain function as mono- and bifunctional CARs and TCRs. Sci. Rep. *9*, 17291.

47. Themeli, M., Kloss, C.C., Ciriello, G., Fedorov, V.D., Perna, F., Gonen, M., and Sadelain, M. (2013). Generation of tumor-targeted human T lymphocytes from induced pluripotent stem cells for cancer therapy. Nat. Biotechnol. *31*, 928–933.

48. Montel-Hagen, A., Seet, C.S., Li, S., Chick, B., Zhu, Y., Chang, P., Tsai, S., Sun, V., Lopez, S., Chen, H.C., et al. (2019). Organoid-Induced Differentiation of Conventional T Cells from Human Pluripotent Stem Cells. Cell Stem Cell *24*, 376–389.e8.

49. Zhao, J., Song, Y., and Liu, D. (2019). Clinical trials of dual-target CAR T cells, donor-derived CAR T cells, and universal CAR T cells for acute lymphoid leukemia. J. Hematol. Oncol. *12*, 17.

50. Jing, R., Scarfo, I., Najia, M.A., Lummertz da Rocha, E., Han, A., Sanborn, M., Bingham, T., Kubaczka, C., Jha, D.K., Falchetti, M., et al. (2022). EZH1 repression generates mature iPSC-derived CAR T cells with enhanced antitumor activity. Cell Stem Cell *29*, 1181–1196.e6.

51. Keam, S.J. (2022). Valemetostat Tosilate: First Approval. Drugs *82*, 1621–1627.

52. van der Stegen, S.J.C., Lindenbergh, P.L., Petrovic, R.M., Xie, H., Diop, M.P., Alexeeva, V., Shi, Y., Mansilla-Soto, J., Hamieh, M., Eyquem, J., et al. (2022). Generation of T-cell-receptor-negative CD8αβ-positive CAR T cells from T-cell-derived induced pluripotent stem cells. Nat. Biomed. Eng. *6*, 1284–1297.

53. Lanitis, E., Rota, G., Kosti, P., Ronet, C., Spill, A., Seijo, B., Romero, P., Dangaj, D., Coukos, G., and Irving, M. (2021). Optimized gene engineering of murine CAR-T cells reveals the beneficial effects of IL-15 coexpression. J. Exp. Med. *218*, e20192203.

54. Kokalaki, E., Ma, B., Ferrari, M., Grothier, T., Hazelton, W., Manzoor, S., Costu, E., Taylor, J., Bulek, A., Srivastava, S., et al. (2023). Dual targeting of CD19 and CD22 against B-ALL using a novel high-sensitivity aCD22 CAR. Mol. Ther. *31*, I2089–I2104.

55. Hua, J., Qian, W., Wu, X., Zhou, L., Yu, L., Chen, S., Zhang, J., and Qiu, H. (2020). Sequential infusion of anti-CD22 and anti-CD19 chimeric antigen receptor T cells for a pediatric Ph-like B-ALL patient that relapsed after CART-cell and Haplo-HSCT therapy: A case report and review of literature. OncoTargets Ther. *13*, 2311–2317.

56. Albinger, N., Hartmann, J., and Ullrich, E. (2021). Current status and perspective of CAR-T and CAR-NK cell therapy trials in Germany. Gene Ther. *28*, 513–527.

57. Mailankody, S., Matous, J.V., Chhabra, S., Liedtke, M., Sidana, S., Oluwole, O.O., Malik, S., Nath, R., Anwer, F., Cruz, J.C., et al. (2023). Allogeneic BCMA-targeting CAR T cells in relapsed/refractory multiple myeloma: phase 1 UNIVERSAL trial interim results. Nat. Med. *2023 292 29*, 422–429.

58. Chang, C., Van Der Stegen, S., Mili, M., Clarke, R., Lai, Y.-S., Witty, A., Lindenbergh, P., Yang, B.-H., Husain, M., Shaked, H., et al. (2019). FT819: Translation of Off-the-Shelf TCR-Less Trac-1XX CAR-T Cells in Support of First-of-Kind Phase I Clinical Trial. Blood *134*, 4434.

59. Park, J.H., Jain, N., Chen, A., McGuirk, J.P., Diaz, M., Valamehr, B., Chu, Y.-W., and Castro, J.E. (2020). A Phase I Study of FT819, a First-of-Kind, Off-the-Shelf, iPSC-Derived TCR-Less CD19 CAR T Cell Therapy for the Treatment of Relapsed/Refractory B-Cell Malignancies. Blood *136*, 15–16.

60. Osborn, M.J., Webber, B.R., Knipping, F., Lonetree, C.L., Tennis, N., DeFeo, A.P., McElroy, A.N., Starker, C.G., Lee, C., Merkel, S., et al. (2016). Evaluation of TCR Gene Editing Achieved by TALENs, CRISPR/Cas9, and megaTAL Nucleases. Mol. Ther. *24*, 570–581.

61. Legut, M., Dolton, G., Mian, A.A., Ottmann, O.G., and Sewell, A.K. (2018). CRISPR-mediated TCR replacement generates superior anticancer transgenic T cells. Blood *131*, 311–322.

62. Watanabe, K., Terakura, S., Martens, A.C., van Meerten, T., Uchiyama, S., Imai, M., Sakemura, R., Goto, T., Hanajiri, R., Imahashi, N., et al. (2015). Target Antigen Density Governs the Efficacy of Anti–CD20-CD28-CD3 ζ Chimeric Antigen Receptor–Modified Effector CD8+ T Cells. J. Immunol. *194*, 911–920.

63. Kamiya, A., Kakinuma, S., Onodera, M., Miyajima, A., and Nakauchi, H. (2008). Prospero-related homeobox 1 and liver receptor homolog 1 coordinated regulate long-term proliferation of murine fetal hepatoblasts. Hepatology *48*, 252–264.

64. Hanatani, T., and Takasu, N. (2021). CiRA iPSC seed stocks (CiRA's iPSC Stock Project). Stem Cell Res. *50*, 102033.

65. Okita, K., Yamakawa, T., Matsumura, Y., Sato, Y., Amano, N., Watanabe, A., Goshima, N., and Yamanaka, S. (2013). An efficient nonviral method to generate integration-free human-induced pluripotent stem cells from cord blood and peripheral blood cells. Stem Cell. *31*, 458–466.

OMTM, Volume *31*

Supplemental information

Mini-TCRs: Truncated T cell receptors to generate T cells from induced pluripotent stem cells

Shin-ichiro Takayanagi, Bo Wang, Saki Hasegawa, Satoshi Nishikawa, Ken Fukumoto, Kohei Nakano, Sayaka Chuganji, Yuya Kato, Sanae Kamibayashi, Atsutaka Minagawa, Atsushi Kunisato, Hajime Nozawa, and Shin Kaneko

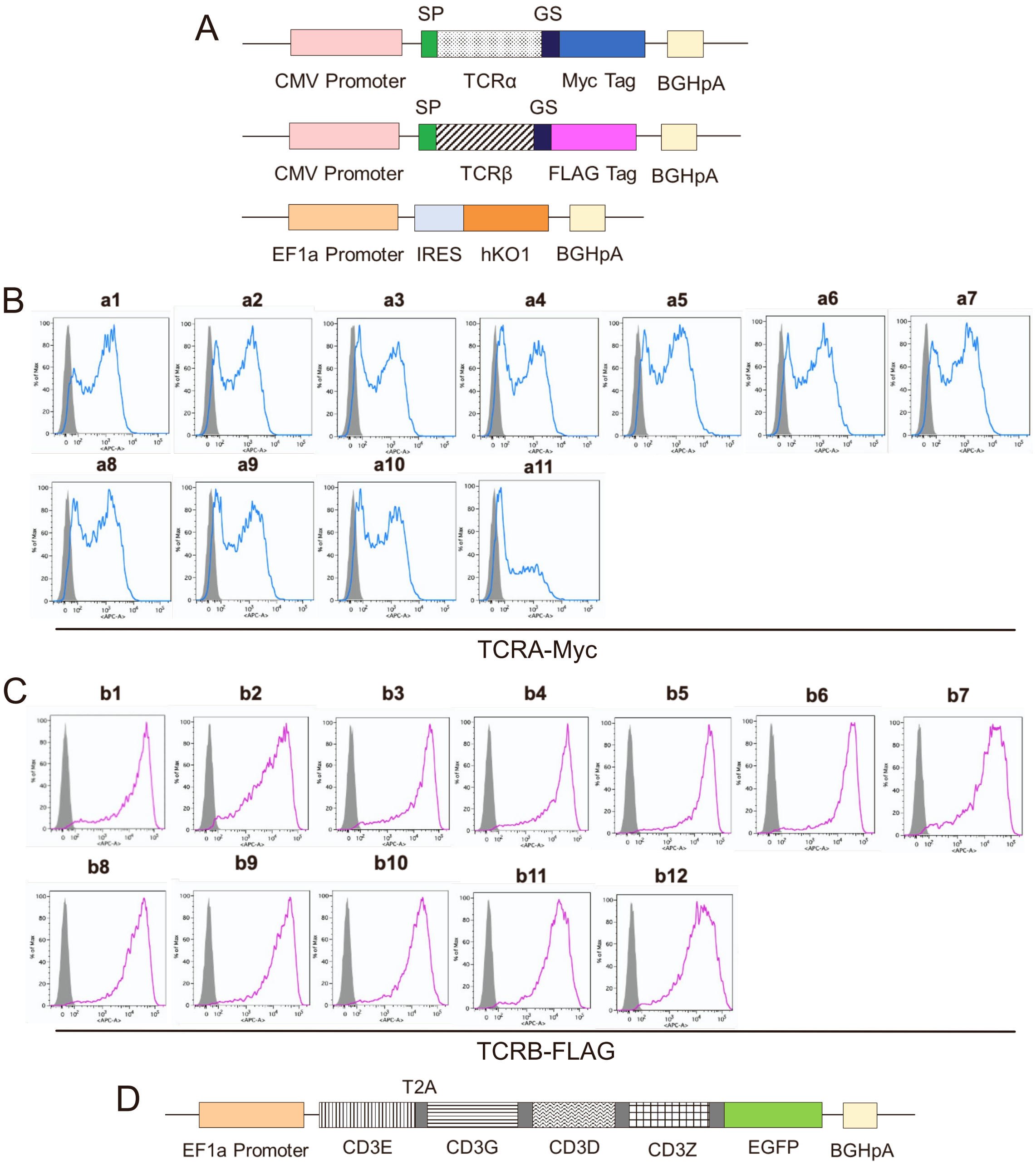

Figure S1, Related to Figure 1. Details of the experimental materials and verification of TCR protein expression.

(A) Structures of the TCRA-Myc (top), TCRB-FLAG (middle), and hKO1 (bottom) expression vectors used in the screening of TCR constructs. hKO1 fluorescent protein was used as a transfection marker.

(B) (C) Intracellular staining to evaluate expression of TCRA-Myc (B) and TCRB-FLAG proteins (C). hKO1+GFP+ cells are shown.

(D) The CD3 protein expression vector used in the CD3-OE-293T cells.

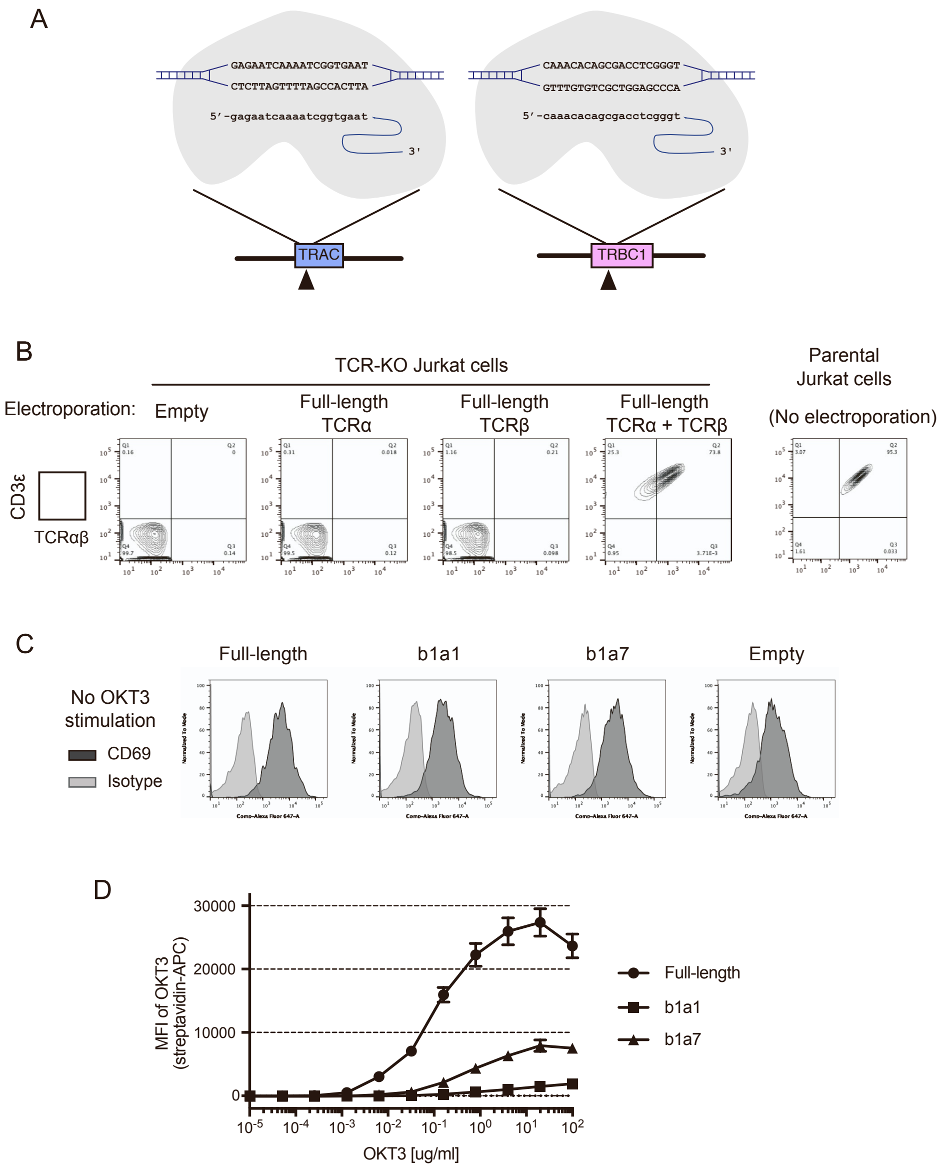

Figure S2, Related to Figure 3. Details of TCR-KO Jurkat cells

(A) gRNA sequences targeting TRAC (left) and TRBC1 (right) loci using the CRISPR/Cas9 method.

(B) FACS plots of TCR-KO and parental Jurkat cells after electroporation of full-length TCRα and/or TCRβ or empty control vector.

(C) Representative FACS plots of CD69 expression on non-stimulated TCR-KO Jurkat cells electroporated with each vector, related to Figure 3E-F.

(D) Mean fluorescence intensity (MFI) of OKT3 to TCR-KO Jurkat cells electroporated with each vector before normalization, related to Figure 3G.

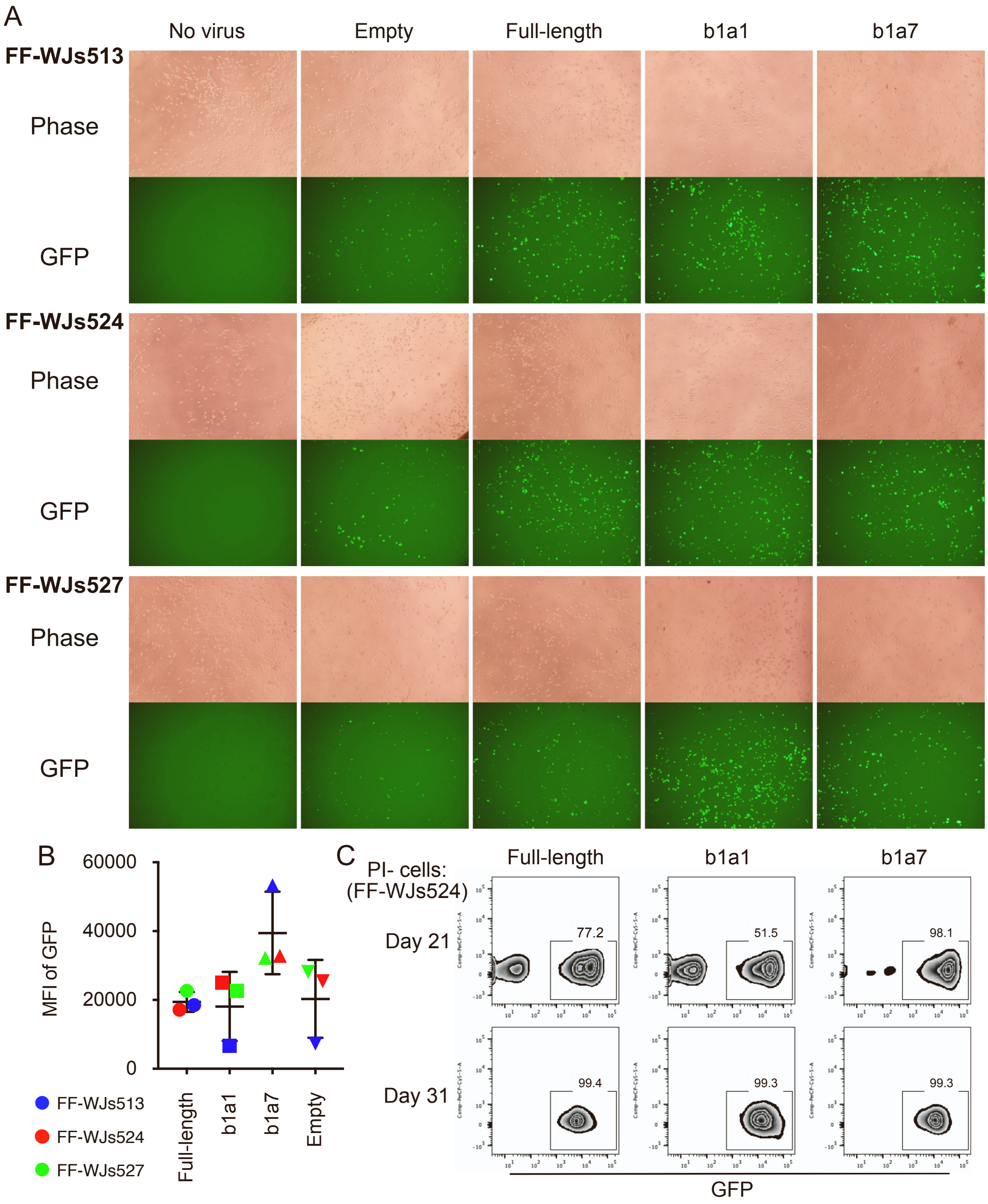

Figure S3, Related to Figure 4.

(A) Fluorescent microscopy images of iPSC-derived HSPCs six days after retroviral transduction.

(B) MFI of GFP in GFP+ cells after T cell induction on DLL4 protein (Day 21).

(C) Flow cytometry before (Day 21) and after (Day 31) T cell maturation with anti-CD3E antibody OKT3 and dexamethasone.

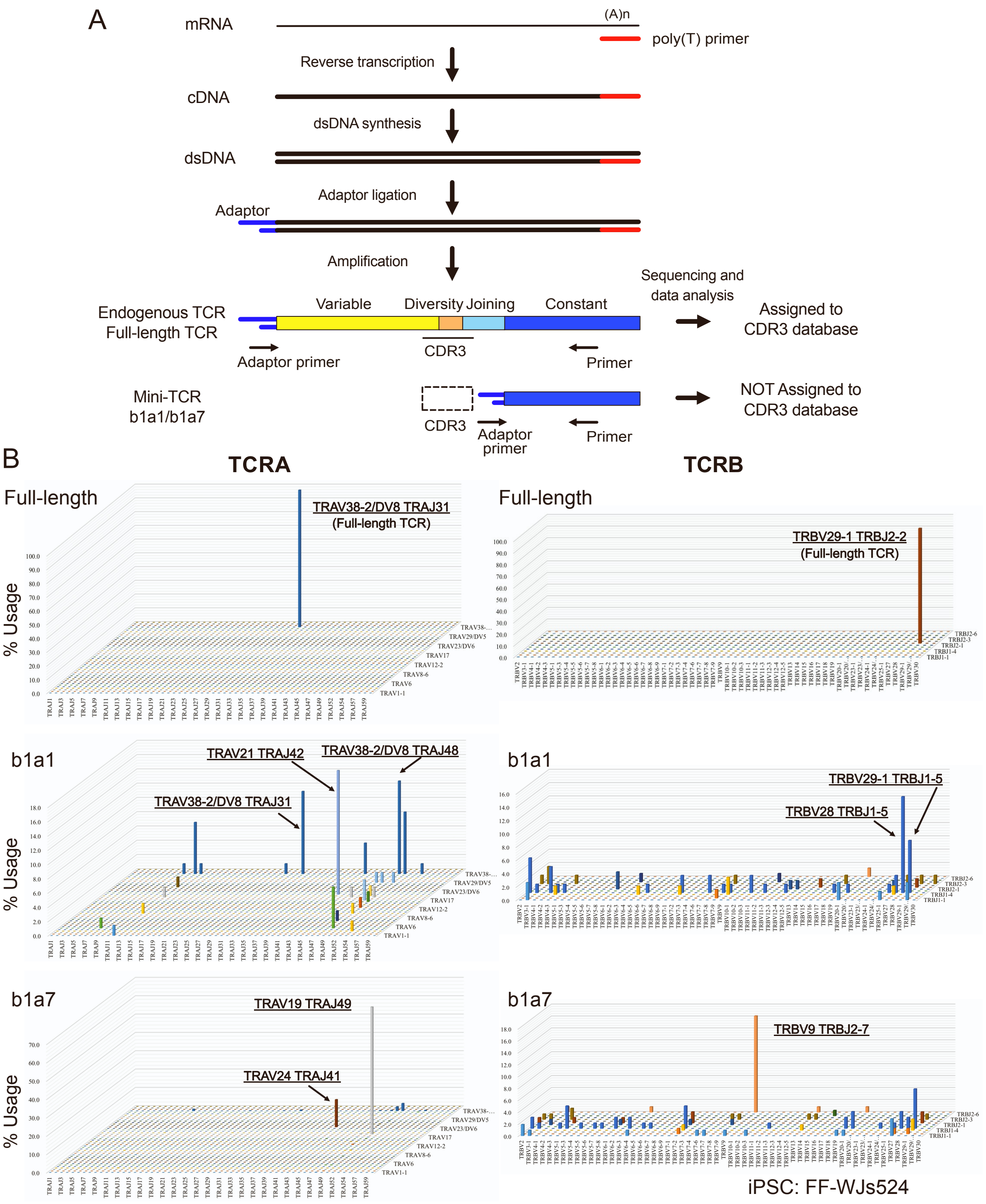

Figure S4, Related to Figure 4. TCR gene expression analysis by Next-generation sequencing

(A) Schematic illustration of NGS-based analysis of TCR gene expression.

(B) TRAVJ (left) and TRBVJ (right) repertoire 3D graph. Data from FF-WJs524 iPSCs are shown as representative of two iPSC-derived CTLs.

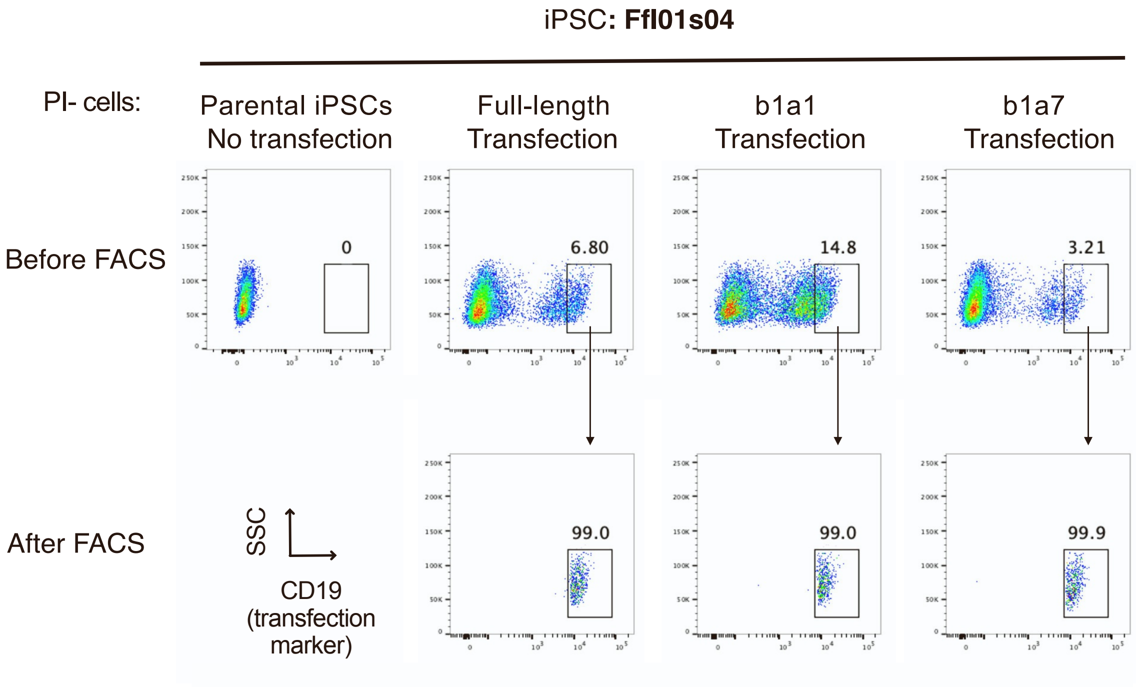

Figure S5, Related to Figure 5. Establishment of Mini-TCR-iPSCs.

Full-length or Mini-TCR b1a1 or b1a7 were inserted into the transposon vector and transfected into iPSCs (clone Ffl01s04). Flow cytometry data before FACS (upper panels) and reanalysis data after FACS to confirm the purity (lower panels). CD19 was used as the transfection marker.

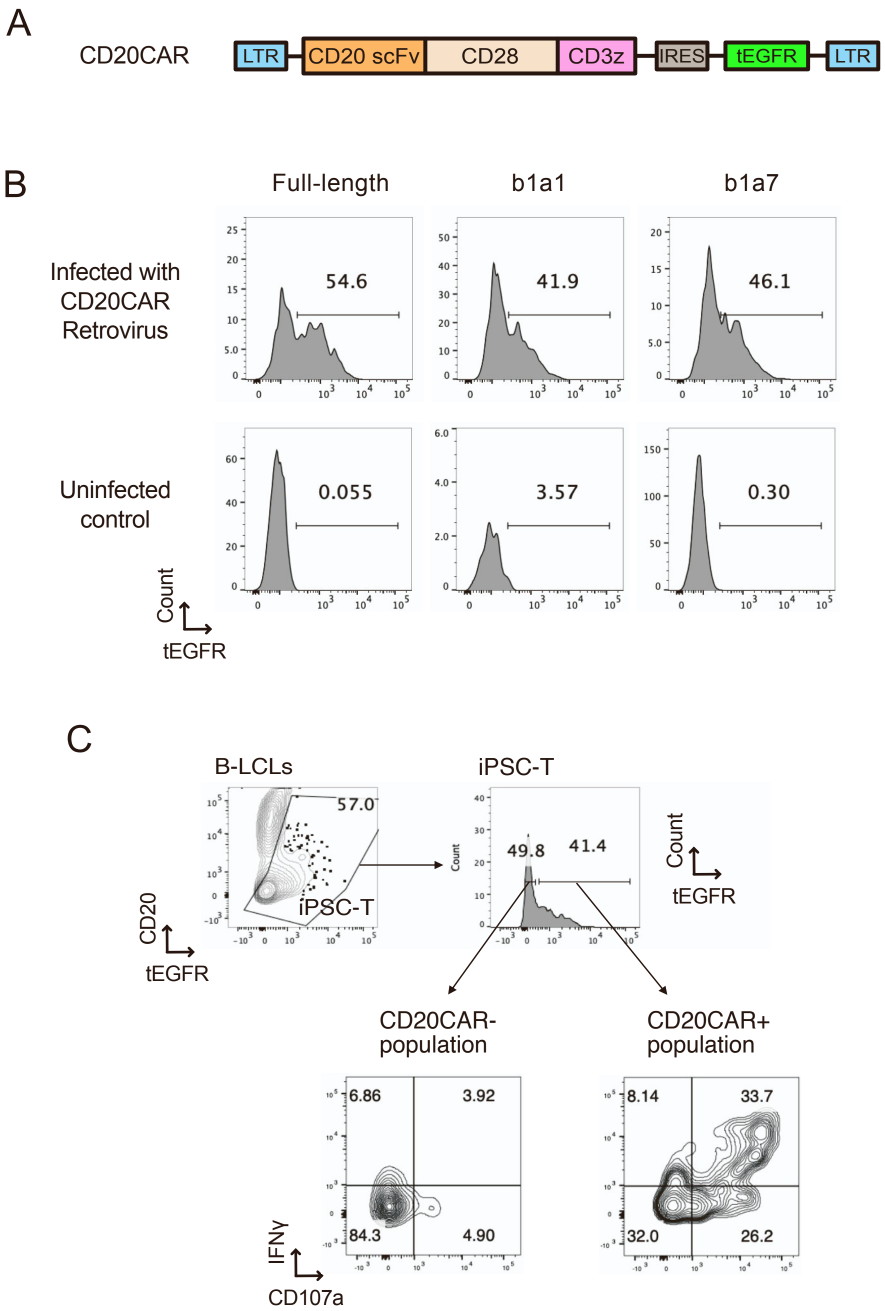

Figure S6, Related to Figure 6. Details of the cytokine production and in vitro cytotoxicity assay.

(A) The structures of the CD20CAR expression vector.

(B) Representative plots of CD20CAR expressing iPSC-derived T cells.

(C) The gating strategy for the cytokine production assay of CD20CAR expressing iPSC-derived CTLs.

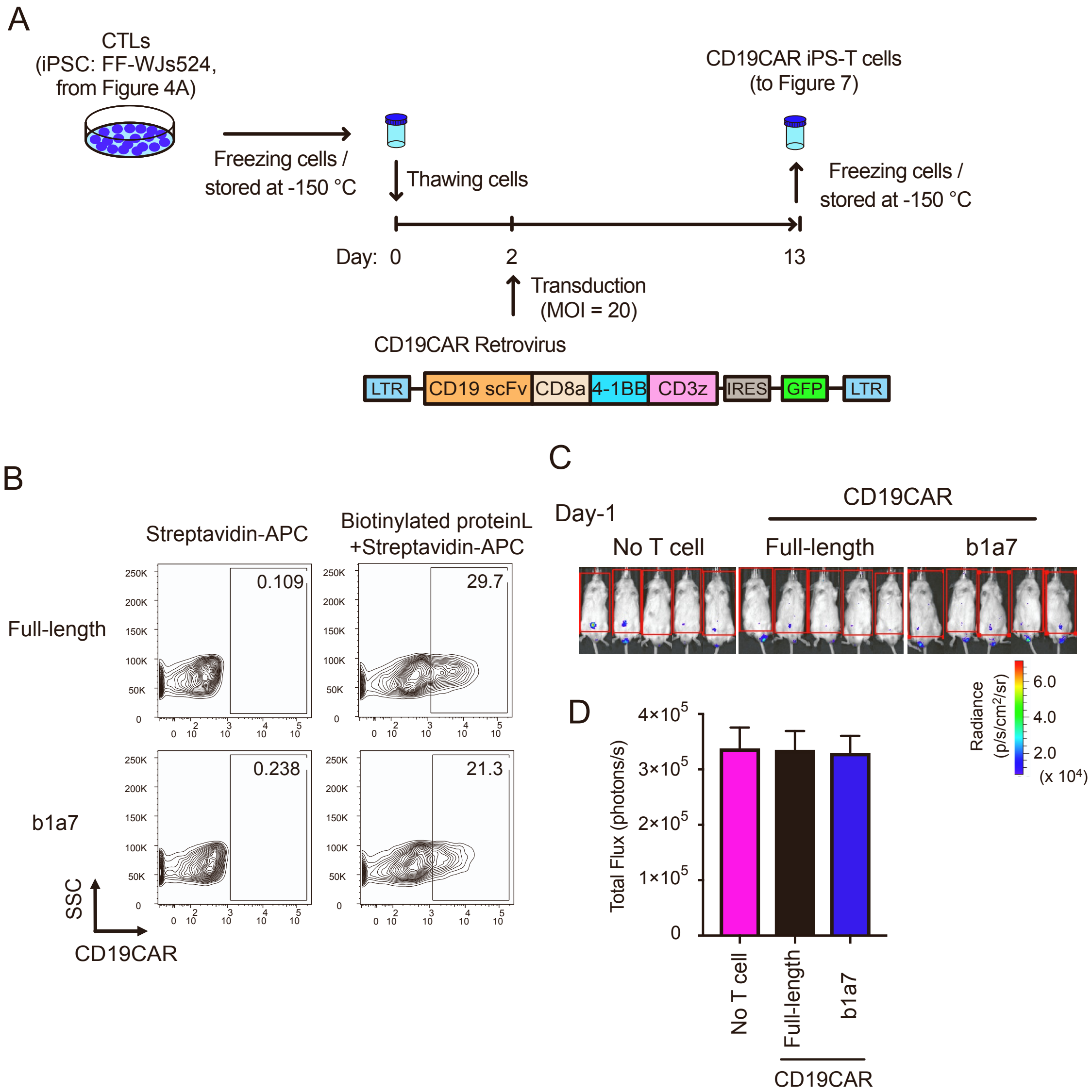

Figure S7, Related to Figure 7. Experimental materials.

(A) Preparation of CD19CAR iPS-T cells and the structure of the CD19CAR vector.

(B) Expression of the CD19CAR protein in iPSC-T cells induced with full-length-TCR or Mini-TCR b1a7. The CD19CAR protein was detected with protein-L that binds to the anti-CD19 single-chain variable fragment (scFv).

(C) *In vivo* bioluminescence images to monitor the amounts of NALM6-Luc-KO cells on Day −1.

(D) Total flux (photons/s) on Day -1. Data are shown as mean ± S.D. of 5 biological replicates.

Table S1. The details of sequencing read in the NGS-based TCRA gene expression analysis

Numbers of sequenced reads were compared with the previous report.[35]

In-frame: Number of reads for which the CDR3 region can be translated to amino acid sequences

Assigned: Number of reads judged as TCRA genes in the Repertoire Genesis software

Total: Number of total reads in NGS

%Read = (Number of reads) / (In-frame read number) x 100

[36]: Data from our earlier report (Minagawa, A. et al. (2018). Enhancing T Cell Receptor Stability in Rejuvenated iPSC-Derived T Cells Improves Their Use in Cancer Immunotherapy. Cell Stem Cell 23, 850–858.e4.)

Sample	Rank	TRAV	TRAJ	CDR3	Reads	%Read
FF-WJs524-Full-length	1	TRAV38-2/DV8	TRAJ31	CAYWSNNNARLMF	107486	96.51
	2	TRAV38-2/DV8	TRAJ31	CAYWSNNNDRLMF	144	0.13
	3	TRAV38-2/DV8	TRAJ31	CAYWSNSNARLMF	107	0.10
	4	TRAV38-2/DV8	TRAJ31	CAYWSNNNVRLMF	106	0.10
	5	TRAV38-2/DV8	TRAJ31	CAYWGNNNARLMF	99	0.09
	In-frame				111368	35.03
	Assigned				113385	35.66
	Total				317963	
FF-WJs524-b1a1	1	TRAV21	TRAJ42	CAVILWRSQGNLIF	12	17.39
	2	TRAV38-2/DV8	TRAJ48	CVSNFGNEKLTF	9	13.04
	3	TRAV38-2/DV8	TRAJ31	CAYWSNNNARLMF	4	5.80
	4	TRAV38-2/DV8	TRAJ12	CGMDSSYKLIF	4	5.80
	5	TRAV38-2/DV8	TRAJ49	CLSQWNTGNQFYF	3	4.35
	In-frame				69	0.02
	Assigned				649	0.19
	Total				338072	
FF-WJs524-b1a7	1	TRAV19	TRAJ49	CALSEASNTGNQFYF	471	67.38
	2	TRAV24	TRAJ41	CAASNSNSGYALNF	98	14.02
	3	TRAV38-2/DV8	TRAJ48	CVSNFGNEKLTF	11	1.57
	4	TRAV38-2/DV8	TRAJ12	CGMDSSYKLIF	5	0.72

	5	TRAV38-2/DV8	TRAJ49	CLSQWNTGNQFYF	4	0.57
	In-frame				699	0.23
	Assigned				1761	0.59
	Total				297521	
FF-WJs527-Full-length	1	TRAV38-2/DV8	TRAJ31	CAYWSNNNARLMF	124717	96.43
	2	TRAV3	TRAJ17	CAVRDSRAAGNKLTF	931	0.72
	3	TRAV38-2/DV8	TRAJ31	CAYWSNNNAGLMF	145	0.11
	4	TRAV38-2/DV8	TRAJ31	CAYWSNNNDRLMF	122	0.09
	5	TRAV38-2/DV8	TRAJ31	CAYWSNSNARLMF	120	0.09
	In-frame				129340	
	Assigned				131259	
	Total				393623	
FF-WJs527-b1a1	1	TRAV38-2/DV8	TRAJ48	CVSNFGNEKLTF	5	16.67
	2	TRAV38-2/DV8	TRAJ31	CAYWSNNNARLMF	3	10.00
	3	TRAV38-2/DV8	TRAJ49	CLSQWNTGNQFYF	2	6.67
	4	TRAV38-2/DV8	TRAJ47	CVEYGNKLVF	2	6.67
	5	TRAV8-4	TRAJ48	CVSNFGNEKLTF	1	3.33
	In-frame				30	
	Assigned				304	
	Total				238460	
FF-WJs527-b1a7	1	TRAV5	TRAJ8	CAEERTGFQKLVF	3815	66.95
	2	TRAV17	TRAJ40	CATAGWGTYKYIF	1349	23.67
	3	TRAV17	TRAJ40	CVTAGWGTYKYIF	117	2.05
	4	TRAV38-2/DV8	TRAJ12	CGMDSSYKLIF	24	0.42
	5	TRAV38-2/DV8	TRAJ48	CVSNFGNEKLTF	21	0.37
	In-frame				5698	
	Assigned				7253	
	Total				440363	
GPC3 T-iPSCs WT[36]	1	TRAV19	TRAJ10	CALTYILTGGGNKLTF	76811	29.29
	2	TRAV1-1	TRAJ33	CAVMDSNYQLIW	6540	2.49
	3	TRAV1-1	TRAJ31	CAVRDNNARLMF	5989	2.28
	4	TRAV9-2	TRAJ4	CALSDSGGYNKLIF	4624	1.76
	5	TRAV1-1	TRAJ34	CAVRDNTDKLIF	3937	1.50

	In-frame				262206	
	Assigned				358316	
	Total				415348	
GPC3 T-iPSCs RAG2 KO[36]	1	TRAV19	TRAJ10	CALTYILTGGGNKLTF	153535	86.31
	2	TRAV19	TRAJ10	CALTYIRTGGGNKLTF	1064	0.60
	3	TRAV19	TRAJ10	CALTDILTGGGNKLTF	764	0.43
	4	TRAV19	TRAJ10	CALTYILTGGGNKRTF	626	0.35
	5	TRAV19	TRAJ10	CALTYMLTGGGNKLTF	617	0.35
	In-frame				177884	
	Assigned				269603	
	Total				338666	
TkT3v1-7 WT[36]	1	TRAV38-2	TRAJ31	CAYWSNNNARLMF	27928	8.17
	2	TRAV39	TRAJ39	CAVVNNAGNMLTF	4270	1.25
	3	TRAV21	TRAJ11	CAVISSGYSTLTF	4122	1.21
	4	TRAV27	TRAJ22	CAGAGSARQLTF	3914	1.15
	5	TRAV30	TRAJ6	CGTSGGSYIPTF	3866	1.13
	In-frame				341648	
	Assigned				421059	
	Total				491249	
TkT3v1-7 RAG2 KO[36]	1	TRAV38-2	TRAJ31	CAYWSNNNARLMF	225909	91.85
	2	TRAV38-2	TRAJ31	CADWSNNNARLMF	747	0.30
	3	TRAV38-2	TRAJ31	CAYWSNSNARLMF	734	0.30
	4	TRAV38-2	TRAJ31	CDYWSNNNARLMF	637	0.26
	5	TRAV38-2	TRAJ31	CAYWSNNNVRLMF	545	0.22
	In-frame				245943	
	Assigned				250110	
	Total				325934	

Table S2. The details of sequencing read in the NGS-based TCRB gene expression analysis

In-frame: Number of reads for which the CDR3 region can be translated to amino acid sequences

Assigned: Number of reads judged as TCRA genes in the Repertoire Genesis software

Total: Number of total reads in NGS

%Read = (Number of reads) / (In-frame read number) x 100

Sample	Rank	TRBV	TRBJ	CDR3	Reads	%Read
FF-WJs524-Full-length	1	TRBV29-1	TRBJ2-2	CSVDGQGNTGELFF	31358	93.19
	2	TRBV29-1	TRBJ2-2	CSVDGQGNTEELFF	68	0.20
	3	TRBV29-1	TRBJ2-2	GSVDGQGNTGELFF	53	0.16
	4	TRBV29-1	TRBJ2-2	CSVDGQRNTGELFF	51	0.15
	5	TRBV29-1	TRBJ2-2	CSVEGQGNTGELFF	43	0.13
	In-frame				33651	
	Assigned				34908	
	Total				484086	
FF-WJs524-b1a1	1	TRBV28	TRBJ1-5	CASSNPGLKQPQHF	10	13.16
	2	TRBV29-1	TRBJ1-5	CSNQPQHF	5	6.58
	3	TRBV2	TRBJ1-5	CSNQPQHF	4	5.26
	4	TRBV9	TRBJ1-4	CATNEKLFF	2	2.63
	5	TRBV7-7	TRBJ1-5	CSNQPQHF	2	2.63
	In-frame				76	
	Assigned				548	
	Total				364343	
FF-WJs524-b1a7	1	TRBV9	TRBJ2-7	CASSVGERWTSGDEQYF	16	15.24
	2	TRBV29-1	TRBJ1-5	CSNQPQHF	6	5.71
	3	TRBV5-1	TRBJ1-5	CSNQPQHF	3	2.86
	4	TRBV7-2	TRBJ2-2	CANTGELFF	2	1.90
	5	TRBV7-2	TRBJ1-5	CSNQPQHF	2	1.90
	In-frame				105	
	Assigned				634	
	Total				339996	

FF-WJs527-Full-length	1	TRBV29-1	TRBJ2-2	CSVDGQGNTGELFF	13328	93.24
	2	TRBV20-1	TRBJ1-5	CSAGNPGTPAGPQHF	31	0.22
	3	TRBV29-1	TRBJ2-2	GSVDGQGNTGELFF	24	0.17
	4	TRBV29-1	TRBJ2-2	CSVDGQGNPGELFF	23	0.16
	5	TRBV29-1	TRBJ2-2	CSVEGQGNTGELFF	22	0.15
	In-frame				14294	
	Assigned				14853	
	Total				331814	
FF-WJs527-b1a1	1	TRBV7-2	TRBJ1-5	CSNQPQHF	5	8.06
	2	TRBV7-2	TRBJ2-2	CANTGELFF	3	4.84
	3	TRBV4-1	TRBJ1-5	CSNQPQHF	3	4.84
	4	TRBV5-4	TRBJ1-5	CSNQPQHF	2	3.23
	5	TRBV3-1	TRBJ1-5	CSNQPQHF	2	3.23
	In-frame				62	
	Assigned				572	
	Total				440286	
FF-WJs527-b1a7	1	TRBV28	TRBJ1-2	CASSRGMGGSGYTF	80	52.98
	2	TRBV29-1	TRBJ1-5	CSNQPQHF	6	3.97
	3	TRBV4-3	TRBJ1-5	CSNQPQHF	3	1.99
	4	TRBV6-3	TRBJ1-5	CSNQPQHF	2	1.32
	5	TRBV29/OR9-2	TRBJ1-5	CSNQPQHF	2	1.32
	In-frame				151	
	Assigned				538	
	Total				383582	

Table S3. cDNA sequences of the full-length TCR and the T2A peptide

Name	DNA sequence
TCRβ	ATGCTGAGTCTTCTGCTCCTTCTCCTGGGACTAGGCTCTGTGTTCAGTGC<br>TGTCATCTCTCAAAAGCCAAGCAGGGATATCTGTCAACGTGGAACCTCCC<br>TGACGATCCAGTGTCAAGTCGATAGCCAAGTCACCATGATGTTCTGGTAC<br>CGTCAGCAACCTGGACAGAGCCTGACACTGATCGCAACTGCAAATCAGG<br>GCTCTGAGGCCACATATGAGAGTGGATTTGTCATTGACAAGTTTCCCATC<br>AGCCGCCCAAACCTAACATTCTCAACTCTGACTGTGAGCAACATGAGCCC<br>TGAAGACAGCAGCATATATCTCTGCAGCGTTGATGGACAGGGAAACACCG<br>GGGAGCTGTTTTTTGGAGAAGGCTCTAGGCTGACCGTACTGGAGGACCT<br>GAAAAACGTGTTCCCACCCGAGGTCGCTGTGTTTGAGCCATCAGAAGCAG<br>AGATCTCCCACACCCAAAAGGCCACACTGGTGTGCCTGGCCACAGGCTT<br>CTACCCCGACCACGTGGAGCTGAGCTGGTGGGTGAATGGGAAGGAGGT<br>GCACAGTGGGGTCAGCACAGACCCGCAGCCCCTCAAGGAGCAGCCCGC<br>CCTCAATGACTCCAGATACTGCCTGAGCAGCCGCCTGAGGGTCTCGGCC<br>ACCTTCTGGCAGAACCCCCGCAACCACTTCCGCTGTCAAGTCCAGTTCTA<br>CGGGCTCTCGGAGAATGACGAGTGGACCCAGGATAGGGCCAAACCTGTC<br>ACCCAGATCGTCAGCGCCGAGGCCTGGGGTAGAGCAGACTGTGGCTTCA<br>CCTCCGAGTCTTACCAGCAAGGGGTCCTGTCTGCCACCATCCTCTATGAG<br>ATCTTGCTAGGGAAGGCCACCTTGTATGCCGTGCTGGTCAGTGCCCTCGT<br>GCTGATGGCCATGGTCAAGAGAAAGGATTCCAGAGGC
T2A peptide	TCCGGAAGCGGAGAGGGCAGAGGAAGTCTGCTAACATGCGGTGACGTCG<br>AGGAGAATCCTGGCCCC
TCRα	ATGGCATGCCCTGGCTTCCTGTGGGCACTTGTGATCTCCACCTGTCTTGA<br>ATTTAGCATGGCTCAGACAGTCACTCAGTCTCAACCAGAGATGTCTGTGC<br>AGGAGGCAGAGACCGTGACCCTGAGCTGCACATATGACACCAGTGAGAG<br>TGATTATTATTTATTCTGGTACAAGCAGCCTCCCAGCAGGCAGATGATTCT<br>CGTTATTCGCCAAGAAGCTTATAAGCAACAGAATGCAACAGAGAATCGTTT<br>CTCTGTGAACTTCCAGAAAGCAGCCAAATCCTTCAGTCTCAAGATCTCAGA<br>CTCACAGCTGGGGGATGCCGCGATGTATTTCTGTGCTTATTGGAGTAATA<br>ACAATGCCAGACTCATGTTTGGAGATGGAACTCAGCTGGTGGTGAAGCCC<br>AATATCCAGAACCCTGACCCTGCCGTGTACCAGCTGAGAGACTCTAAATC<br>CAGTGACAAGTCTGTCTGCCTATTCACCGATTTTGATTCTCAAACAAATGT<br>GTCACAAAGTAAGGATTCTGATGTGTATATCACAGACAAAACTGTGCTAGA<br>CATGAGGTCTATGGACTTCAAGAGCAACAGTGCTGTGGCCTGGAGCAACA<br>AATCTGACTTTGCATGTGCAAACGCCTTCAACAACAGCATTATTCCAGAAG

	ACACCTTCTTCCCCAGCCCAGAAAGTTCCTGTGATGTCAAGCTGGTCGAG<br>AAAAGCTTTGAAACAGATACGAACCTAAACTTTCAAAACCTGTCAGTGATT<br>GGGTTCCGAATCCTCCTCCTGAAAGTGGCCGGGTTTAATCTGCTCATGAC<br>GCTGCGGCTGTGGTCCAGCTGA

Table S4. Amino acid sequences of the full-length-TCR and the T2A peptide

Name	Amino acid sequence
TCRβ	MLSLLLLLLGLGSVFSAVISQKPSRDICQRGTSLTIQCQVDSQVTMMFWYRQ<br>QPGQSLTLIATANQGSEATYESGFVIDKFPISRPNLTFSTLTVSNMSPEDSSIY<br>LCSVDGQGNTGELFFGEGSRLTVLEDLKNVFPPEVAVFEPSEAEISHTQKATL<br>VCLATGFYPDHVELSWWVNGKEVHSGVSTDPQPLKEQPALNDSRYCLSSRL<br>RVSATFWQNPRNHFRCQVQFYGLSENDEWTQDRAKPVTQIVSAEAWGRAD<br>CGFTSESYQQGVLSATILYEILLGKATLYAVLVSALVLMAMVKRKDSRG
T2A peptide	SGSGEGRGSLLTCGDVEENPGP
TCRα	MACPGFLWALVISTCLEFSMAQTVTQSQPEMSVQEAETVTLSCTYDTSESDY<br>YLFWYKQPPSRQMILVIRQEAYKQQNATENRFSVNFQKAAKSFSLKISDSQL<br>GDAAMYFCAYWSNNNARLMFGDGTQLVVKPNIQNPDPAVYQLRDSKSSDK<br>SVCLFTDFDSQTNVSQSKDSDVYITDKTVLDMRSMDFKSNSAVAWSNKSDF<br>ACANAFNNSIIPEDTFFPSPESSCDVKLVEKSFETDTNLNFQNLSVIGFRILLLK<br>VAGFNLLMTLRLWSS

Table S5. cDNA sequences of TCRα constructs

Name	Length	DNA sequence
α1	423	AATATCCAGAACCCTGACCCTGCCGTGTACCAGCTGAGAGACTCTA<br>AATCCAGTGACAAGTCTGTCTGCCTATTCACCGATTTTGATTCTCAA<br>ACAAATGTGTCACAAAGTAAGGATTCTGATGTGTATATCACAGACAA<br>AACTGTGCTAGACATGAGGTCTATGGACTTCAAGAGCAACAGTGCT<br>GTGGCCTGGAGCAACAAATCTGACTTTGCATGTGCAAACGCCTTCA<br>ACAACAGCATTATTCCAGAAGACACCTTCTTCCCCAGCCCAGAAAG<br>TTCCTGTGATGTCAAGCTGGTCGAGAAAAGCTTTGAAACAGATACG<br>AACCTAAACTTTCAAAACCTGTCAGTGATTGGGTTCCGAATCCTCCT<br>CCTGAAAGTGGCCGGGTTTAATCTGCTCATGACGCTGCGGCTGTG<br>GTCCAGC
α2	396	TACCAGCTGAGAGACTCTAAATCCAGTGACAAGTCTGTCTGCCTATT<br>CACCGATTTTGATTCTCAAACAAATGTGTCACAAAGTAAGGATTCTG<br>ATGTGTATATCACAGACAAAACTGTGCTAGACATGAGGTCTATGGA<br>CTTCAAGAGCAACAGTGCTGTGGCCTGGAGCAACAAATCTGACTTT<br>GCATGTGCAAACGCCTTCAACAACAGCATTATTCCAGAAGACACCT<br>TCTTCCCCAGCCCAGAAAGTTCCTGTGATGTCAAGCTGGTCGAGAA<br>AAGCTTTGAAACAGATACGAACCTAAACTTTCAAAACCTGTCAGTGA<br>TTGGGTTCCGAATCCTCCTCCTGAAAGTGGCCGGGTTTAATCTGCT<br>CATGACGCTGCGGCTGTGGTCCAGC
α3	363	TCTGTCTGCCTATTCACCGATTTTGATTCTCAAACAAATGTGTCACA<br>AAGTAAGGATTCTGATGTGTATATCACAGACAAAACTGTGCTAGACA<br>TGAGGTCTATGGACTTCAAGAGCAACAGTGCTGTGGCCTGGAGCAA<br>CAAATCTGACTTTGCATGTGCAAACGCCTTCAACAACAGCATTATTC<br>CAGAAGACACCTTCTTCCCCAGCCCAGAAAGTTCCTGTGATGTCAA<br>GCTGGTCGAGAAAAGCTTTGAAACAGATACGAACCTAAACTTTCAAA<br>ACCTGTCAGTGATTGGGTTCCGAATCCTCCTCCTGAAAGTGGCCGG<br>GTTTAATCTGCTCATGACGCTGCGGCTGTGGTCCAGC
α4	327	AATGTGTCACAAAGTAAGGATTCTGATGTGTATATCACAGACAAAAC<br>TGTGCTAGACATGAGGTCTATGGACTTCAAGAGCAACAGTGCTGTG<br>GCCTGGAGCAACAAATCTGACTTTGCATGTGCAAACGCCTTCAACA<br>ACAGCATTATTCCAGAAGACACCTTCTTCCCCAGCCCAGAAAGTTC<br>CTGTGATGTCAAGCTGGTCGAGAAAAGCTTTGAAACAGATACGAAC<br>CTAAACTTTCAAAACCTGTCAGTGATTGGGTTCCGAATCCTCCTCCT<br>GAAAGTGGCCGGGTTTAATCTGCTCATGACGCTGCGGCTGTGGTC

		CAGC
α5	303	GATGTGTATATCACAGACAAAACTGTGCTAGACATGAGGTCTATGG<br>ACTTCAAGAGCAACAGTGCTGTGGCCTGGAGCAACAAATCTGACTT<br>TGCATGTGCAAACGCCTTCAACAACAGCATTATTCCAGAAGACACC<br>TTCTTCCCCAGCCCAGAAAGTTCCTGTGATGTCAAGCTGGTCGAGA<br>AAAGCTTTGAAACAGATACGAACCTAAACTTTCAAAACCTGTCAGTG<br>ATTGGGTTCCGAATCCTCCTCCTGAAAGTGGCCGGGTTTAATCTGC<br>TCATGACGCTGCGGCTGTGGTCCAGC
α6	276	CTAGACATGAGGTCTATGGACTTCAAGAGCAACAGTGCTGTGGCCT<br>GGAGCAACAAATCTGACTTTGCATGTGCAAACGCCTTCAACAACAG<br>CATTATTCCAGAAGACACCTTCTTCCCCAGCCCAGAAAGTTCCTGT<br>GATGTCAAGCTGGTCGAGAAAAGCTTTGAAACAGATACGAACCTAA<br>ACTTTCAAAACCTGTCAGTGATTGGGTTCCGAATCCTCCTCCTGAAA<br>GTGGCCGGGTTTAATCTGCTCATGACGCTGCGGCTGTGGTCCAGC
α7	252	AAGAGCAACAGTGCTGTGGCCTGGAGCAACAAATCTGACTTTGCAT<br>GTGCAAACGCCTTCAACAACAGCATTATTCCAGAAGACACCTTCTTC<br>CCCAGCCCAGAAAGTTCCTGTGATGTCAAGCTGGTCGAGAAAAGCT<br>TTGAAACAGATACGAACCTAAACTTTCAAAACCTGTCAGTGATTGGG<br>TTCCGAATCCTCCTCCTGAAAGTGGCCGGGTTTAATCTGCTCATGA<br>CGCTGCGGCTGTGGTCCAGC
α8	228	AGCAACAAATCTGACTTTGCATGTGCAAACGCCTTCAACAACAGCAT<br>TATTCCAGAAGACACCTTCTTCCCCAGCCCAGAAAGTTCCTGTGAT<br>GTCAAGCTGGTCGAGAAAAGCTTTGAAACAGATACGAACCTAAACT<br>TTCAAAACCTGTCAGTGATTGGGTTCCGAATCCTCCTCCTGAAAGT<br>GGCCGGGTTTAATCTGCTCATGACGCTGCGGCTGTGGTCCAGC
α9	207	TGTGCAAACGCCTTCAACAACAGCATTATTCCAGAAGACACCTTCTT<br>CCCCAGCCCAGAAAGTTCCTGTGATGTCAAGCTGGTCGAGAAAAGC<br>TTTGAAACAGATACGAACCTAAACTTTCAAAACCTGTCAGTGATTGG<br>GTTCCGAATCCTCCTCCTGAAAGTGGCCGGGTTTAATCTGCTCATG<br>ACGCTGCGGCTGTGGTCCAGC
α10	195	TTCAACAACAGCATTATTCCAGAAGACACCTTCTTCCCCAGCCCAGA<br>AAGTTCCTGTGATGTCAAGCTGGTCGAGAAAAGCTTTGAAACAGAT<br>ACGAACCTAAACTTTCAAAACCTGTCAGTGATTGGGTTCCGAATCCT<br>CCTCCTGAAAGTGGCCGGGTTTAATCTGCTCATGACGCTGCGGCTG<br>TGGTCCAGC

α11	180	ATTCCAGAAGACACCTTCTTCCCCAGCCCAGAAAGTTCCTGTGATG TCAAGCTGGTCGAGAAAAGCTTTGAAACAGATACGAACCTAAACTTT CAAAACCTGTCAGTGATTGGGTTCCGAATCCTCCTCCTGAAAGTGG CCGGGTTTAATCTGCTCATGACGCTGCGGCTGTGGTCCAGC

Table S6. cDNA sequences of TCRβ constructs

Name	Length	DNA sequence
β1	537	GAGGACCTGAAAAACGTGTTCCCACCCGAGGTCGCTGTGTTTGAG<br>CCATCAGAAGCAGAGATCTCCCACACCCAAAAGGCCACACTGGTGT<br>GCCTGGCCACAGGCTTCTACCCCGACCACGTGGAGCTGAGCTGGT<br>GGGTGAATGGGAAGGAGGTGCACAGTGGGGTCAGCACAGACCCG<br>CAGCCCCTCAAGGAGCAGCCCGCCCTCAATGACTCCAGATACTGC<br>CTGAGCAGCCGCCTGAGGGTCTCGGCCACCTTCTGGCAGAACCCC<br>CGCAACCACTTCCGCTGTCAAGTCCAGTTCTACGGGCTCTCGGAGA<br>ATGACGAGTGGACCCAGGATAGGGCCAAACCTGTCACCCAGATCG<br>TCAGCGCCGAGGCCTGGGGTAGAGCAGACTGTGGCTTCACCTCCG<br>AGTCTTACCAGCAAGGGGTCCTGTCTGCCACCATCCTCTATGAGAT<br>CTTGCTAGGGAAGGCCACCTTGTATGCCGTGCTGGTCAGTGCCCT<br>CGTGCTGATGGCCATGGTCAAGAGAAAGGATTCCAGAGGC
β2	489	TCAGAAGCAGAGATCTCCCACACCCAAAAGGCCACACTGGTGTGC<br>CTGGCCACAGGCTTCTACCCCGACCACGTGGAGCTGAGCTGGTGG<br>GTGAATGGGAAGGAGGTGCACAGTGGGGTCAGCACAGACCCGCA<br>GCCCCTCAAGGAGCAGCCCGCCCTCAATGACTCCAGATACTGCCT<br>GAGCAGCCGCCTGAGGGTCTCGGCCACCTTCTGGCAGAACCCCCG<br>CAACCACTTCCGCTGTCAAGTCCAGTTCTACGGGCTCTCGGAGAAT<br>GACGAGTGGACCCAGGATAGGGCCAAACCTGTCACCCAGATCGTC<br>AGCGCCGAGGCCTGGGGTAGAGCAGACTGTGGCTTCACCTCCGAG<br>TCTTACCAGCAAGGGGTCCTGTCTGCCACCATCCTCTATGAGATCT<br>TGCTAGGGAAGGCCACCTTGTATGCCGTGCTGGTCAGTGCCCTCG<br>TGCTGATGGCCATGGTCAAGAGAAAGGATTCCAGAGGC
β3	459	GCCACACTGGTGTGCCTGGCCACAGGCTTCTACCCCGACCACGTG<br>GAGCTGAGCTGGTGGGTGAATGGGAAGGAGGTGCACAGTGGGGT<br>CAGCACAGACCCGCAGCCCCTCAAGGAGCAGCCCGCCCTCAATGA<br>CTCCAGATACTGCCTGAGCAGCCGCCTGAGGGTCTCGGCCACCTT<br>CTGGCAGAACCCCCGCAACCACTTCCGCTGTCAAGTCCAGTTCTAC<br>GGGCTCTCGGAGAATGACGAGTGGACCCAGGATAGGGCCAAACCT<br>GTCACCCAGATCGTCAGCGCCGAGGCCTGGGGTAGAGCAGACTGT<br>GGCTTCACCTCCGAGTCTTACCAGCAAGGGGTCCTGTCTGCCACCA<br>TCCTCTATGAGATCTTGCTAGGGAAGGCCACCTTGTATGCCGTGCT<br>GGTCAGTGCCCTCGTGCTGATGGCCATGGTCAAGAGAAAGGATTC<br>CAGAGGC

β4	417	GTGGAGCTGAGCTGGTGGGTGAATGGGAAGGAGGTGCACAGTGG<br>GGTCAGCACAGACCCGCAGCCCCTCAAGGAGCAGCCCGCCCTCAA<br>TGACTCCAGATACTGCCTGAGCAGCCGCCTGAGGGTCTCGGCCAC<br>CTTCTGGCAGAACCCCCGCAACCACTTCCGCTGTCAAGTCCAGTTC<br>TACGGGCTCTCGGAGAATGACGAGTGGACCCAGGATAGGGCCAAA<br>CCTGTCACCCAGATCGTCAGCGCCGAGGCCTGGGGTAGAGCAGAC<br>TGTGGCTTCACCTCCGAGTCTTACCAGCAAGGGGTCCTGTCTGCCA<br>CCATCCTCTATGAGATCTTGCTAGGGAAGGCCACCTTGTATGCCGT<br>GCTGGTCAGTGCCCTCGTGCTGATGGCCATGGTCAAGAGAAAGGA<br>TTCCAGAGGC
β5	399	GTGAATGGGAAGGAGGTGCACAGTGGGGTCAGCACAGACCCGCA<br>GCCCCTCAAGGAGCAGCCCGCCCTCAATGACTCCAGATACTGCCT<br>GAGCAGCCGCCTGAGGGTCTCGGCCACCTTCTGGCAGAACCCCCG<br>CAACCACTTCCGCTGTCAAGTCCAGTTCTACGGGCTCTCGGAGAAT<br>GACGAGTGGACCCAGGATAGGGCCAAACCTGTCACCCAGATCGTC<br>AGCGCCGAGGCCTGGGGTAGAGCAGACTGTGGCTTCACCTCCGAG<br>TCTTACCAGCAAGGGGTCCTGTCTGCCACCATCCTCTATGAGATCT<br>TGCTAGGGAAGGCCACCTTGTATGCCGTGCTGGTCAGTGCCCTCG<br>TGCTGATGGCCATGGTCAAGAGAAAGGATTCCAGAGGC
β6	372	GTCAGCACAGACCCGCAGCCCCTCAAGGAGCAGCCCGCCCTCAAT<br>GACTCCAGATACTGCCTGAGCAGCCGCCTGAGGGTCTCGGCCACC<br>TTCTGGCAGAACCCCCGCAACCACTTCCGCTGTCAAGTCCAGTTCT<br>ACGGGCTCTCGGAGAATGACGAGTGGACCCAGGATAGGGCCAAAC<br>CTGTCACCCAGATCGTCAGCGCCGAGGCCTGGGGTAGAGCAGACT<br>GTGGCTTCACCTCCGAGTCTTACCAGCAAGGGGTCCTGTCTGCCAC<br>CATCCTCTATGAGATCTTGCTAGGGAAGGCCACCTTGTATGCCGTG<br>CTGGTCAGTGCCCTCGTGCTGATGGCCATGGTCAAGAGAAAGGATT<br>CCAGAGGC
β7	336	GCCCTCAATGACTCCAGATACTGCCTGAGCAGCCGCCTGAGGGTC<br>TCGGCCACCTTCTGGCAGAACCCCCGCAACCACTTCCGCTGTCAA<br>GTCCAGTTCTACGGGCTCTCGGAGAATGACGAGTGGACCCAGGAT<br>AGGGCCAAACCTGTCACCCAGATCGTCAGCGCCGAGGCCTGGGGT<br>AGAGCAGACTGTGGCTTCACCTCCGAGTCTTACCAGCAAGGGGTC<br>CTGTCTGCCACCATCCTCTATGAGATCTTGCTAGGGAAGGCCACCT<br>TGTATGCCGTGCTGGTCAGTGCCCTCGTGCTGATGGCCATGGTCAA<br>GAGAAAGGATTCCAGAGGC

β8	303	CGCCTGAGGGTCTCGGCCACCTTCTGGCAGAACCCCCGCAACCAC<br>TTCCGCTGTCAAGTCCAGTTCTACGGGCTCTCGGAGAATGACGAGT<br>GGACCCAGGATAGGGCCAAACCTGTCACCCAGATCGTCAGCGCCG<br>AGGCCTGGGGTAGAGCAGACTGTGGCTTCACCTCCGAGTCTTACC<br>AGCAAGGGGTCCTGTCTGCCACCATCCTCTATGAGATCTTGCTAGG<br>GAAGGCCACCTTGTATGCCGTGCTGGTCAGTGCCCTCGTGCTGAT<br>GGCCATGGTCAAGAGAAAGGATTCCAGAGGC
β9	276	CAGAACCCCCGCAACCACTTCCGCTGTCAAGTCCAGTTCTACGGGC<br>TCTCGGAGAATGACGAGTGGACCCAGGATAGGGCCAAACCTGTCA<br>CCCAGATCGTCAGCGCCGAGGCCTGGGGTAGAGCAGACTGTGGCT<br>TCACCTCCGAGTCTTACCAGCAAGGGGTCCTGTCTGCCACCATCCT<br>CTATGAGATCTTGCTAGGGAAGGCCACCTTGTATGCCGTGCTGGTC<br>AGTGCCCTCGTGCTGATGGCCATGGTCAAGAGAAAGGATTCCAGA<br>GGC
β10	258	TTCCGCTGTCAAGTCCAGTTCTACGGGCTCTCGGAGAATGACGAGT<br>GGACCCAGGATAGGGCCAAACCTGTCACCCAGATCGTCAGCGCCG<br>AGGCCTGGGGTAGAGCAGACTGTGGCTTCACCTCCGAGTCTTACC<br>AGCAAGGGGTCCTGTCTGCCACCATCCTCTATGAGATCTTGCTAGG<br>GAAGGCCACCTTGTATGCCGTGCTGGTCAGTGCCCTCGTGCTGAT<br>GGCCATGGTCAAGAGAAAGGATTCCAGAGGC
β11	237	TACGGGCTCTCGGAGAATGACGAGTGGACCCAGGATAGGGCCAAA<br>CCTGTCACCCAGATCGTCAGCGCCGAGGCCTGGGGTAGAGCAGAC<br>TGTGGCTTCACCTCCGAGTCTTACCAGCAAGGGGTCCTGTCTGCCA<br>CCATCCTCTATGAGATCTTGCTAGGGAAGGCCACCTTGTATGCCGT<br>GCTGGTCAGTGCCCTCGTGCTGATGGCCATGGTCAAGAGAAAGGA<br>TTCCAGAGGC
β12	216	GAGTGGACCCAGGATAGGGCCAAACCTGTCACCCAGATCGTCAGC<br>GCCGAGGCCTGGGGTAGAGCAGACTGTGGCTTCACCTCCGAGTCT<br>TACCAGCAAGGGGTCCTGTCTGCCACCATCCTCTATGAGATCTTGC<br>TAGGGAAGGCCACCTTGTATGCCGTGCTGGTCAGTGCCCTCGTGC<br>TGATGGCCATGGTCAAGAGAAAGGATTCCAGAGGC
β13	198	GCCAAACCTGTCACCCAGATCGTCAGCGCCGAGGCCTGGGGTAGA<br>GCAGACTGTGGCTTCACCTCCGAGTCTTACCAGCAAGGGGTCCTGT<br>CTGCCACCATCCTCTATGAGATCTTGCTAGGGAAGGCCACCTTGTA<br>TGCCGTGCTGGTCAGTGCCCTCGTGCTGATGGCCATGGTCAAGAG<br>AAAGGATTCCAGAGGC

Table S7. Amino acid sequences of TCRα constructs

Name	Length	Amino acid sequence
α1	141	NIQNPDPAVYQLRDSKSSDKSVCLFTDFDSQTNVSQSKDSDVYITDKTVLDMRSMDFKSNSAVAWSNKSDFACANAFNNSIIPEDTFFPSPESSCDVKLVEKSFETDTNLNFQNLSVIGFRILLLKVAGFNLLMTLRLWSS
α2	132	YQLRDSKSSDKSVCLFTDFDSQTNVSQSKDSDVYITDKTVLDMRSMDFKSNSAVAWSNKSDFACANAFNNSIIPEDTFFPSPESSCDVKLVEKSFETDTNLNFQNLSVIGFRILLLKVAGFNLLMTLRLWSS
α3	121	SVCLFTDFDSQTNVSQSKDSDVYITDKTVLDMRSMDFKSNSAVAWSNKSDFACANAFNNSIIPEDTFFPSPESSCDVKLVEKSFETDTNLNFQNLSVIGFRILLLKVAGFNLLMTLRLWSS
α4	109	NVSQSKDSDVYITDKTVLDMRSMDFKSNSAVAWSNKSDFACANAFNNSIIPEDTFFPSPESSCDVKLVEKSFETDTNLNFQNLSVIGFRILLLKVAGFNLLMTLRLWSS
α5	101	DVYITDKTVLDMRSMDFKSNSAVAWSNKSDFACANAFNNSIIPEDTFFPSPESSCDVKLVEKSFETDTNLNFQNLSVIGFRILLLKVAGFNLLMTLRLWSS
α6	92	LDMRSMDFKSNSAVAWSNKSDFACANAFNNSIIPEDTFFPSPESSCDVKLVEKSFETDTNLNFQNLSVIGFRILLLKVAGFNLLMTLRLWSS
α7	84	KSNSAVAWSNKSDFACANAFNNSIIPEDTFFPSPESSCDVKLVEKSFETDTNLNFQNLSVIGFRILLLKVAGFNLLMTLRLWSS
α8	76	SNKSDFACANAFNNSIIPEDTFFPSPESSCDVKLVEKSFETDTNLNFQNLSVIGFRILLLKVAGFNLLMTLRLWSS
α9	69	CANAFNNSIIPEDTFFPSPESSCDVKLVEKSFETDTNLNFQNLSVIGFRILLLKVAGFNLLMTLRLWSS
α10	65	FNNSIIPEDTFFPSPESSCDVKLVEKSFETDTNLNFQNLSVIGFRILLLKVAGFNLLMTLRLWSS
α11	60	IPEDTFFPSPESSCDVKLVEKSFETDTNLNFQNLSVIGFRILLLKVAGFNLLMTLRLWSS

Table S8. Amino acid sequences of TCRβ constructs

Name	Length	Amino acid sequence
β1	179	EDLKNVFPPEVAVFEPSEAEISHTQKATLVCLATGFYPDHVELSWWVNGKEVHSGVSTDPQPLKEQPALNDSRYCLSSRLRVSATFWQNPRNHFRCQVQFYGLSENDEWTQDRAKPVTQIVSAEAWGRADCGFTSESYQQGVLSATILYEILLGKATLYAVLVSALVLMAMVKRKDSRG
β2	163	SEAEISHTQKATLVCLATGFYPDHVELSWWVNGKEVHSGVSTDPQPLKEQPALNDSRYCLSSRLRVSATFWQNPRNHFRCQVQFYGLSENDEWTQDRAKPVTQIVSAEAWGRADCGFTSESYQQGVLSATILYEILLGKATLYAVLVSALVLMAMVKRKDSRG
β3	153	ATLVCLATGFYPDHVELSWWVNGKEVHSGVSTDPQPLKEQPALNDSRYCLSSRLRVSATFWQNPRNHFRCQVQFYGLSENDEWTQDRAKPVTQIVSAEAWGRADCGFTSESYQQGVLSATILYEILLGKATLYAVLVSALVLMAMVKRKDSRG
β4	139	VELSWWVNGKEVHSGVSTDPQPLKEQPALNDSRYCLSSRLRVSATFWQNPRNHFRCQVQFYGLSENDEWTQDRAKPVTQIVSAEAWGRADCGFTSESYQQGVLSATILYEILLGKATLYAVLVSALVLMAMVKRKDSRG
β5	133	VNGKEVHSGVSTDPQPLKEQPALNDSRYCLSSRLRVSATFWQNPRNHFRCQVQFYGLSENDEWTQDRAKPVTQIVSAEAWGRADCGFTSESYQQGVLSATILYEILLGKATLYAVLVSALVLMAMVKRKDSRG
β6	124	VSTDPQPLKEQPALNDSRYCLSSRLRVSATFWQNPRNHFRCQVQFYGLSENDEWTQDRAKPVTQIVSAEAWGRADCGFTSESYQQGVLSATILYEILLGKATLYAVLVSALVLMAMVKRKDSRG
β7	112	ALNDSRYCLSSRLRVSATFWQNPRNHFRCQVQFYGLSENDEWTQDRAKPVTQIVSAEAWGRADCGFTSESYQQGVLSATILYEILLGKATLYAVLVSALVLMAMVKRKDSRG
β8	101	RLRVSATFWQNPRNHFRCQVQFYGLSENDEWTQDRAKPVTQIVSAEAWGRADCGFTSESYQQGVLSATILYEILLGKATLYAVLVSALVLMAMVKRKDSRG
β9	92	QNPRNHFRCQVQFYGLSENDEWTQDRAKPVTQIVSAEAWGRADCGFTSESYQQGVLSATILYEILLGKATLYAVLVSALVLMAMVKRKDSRG
β10	86	FRCQVQFYGLSENDEWTQDRAKPVTQIVSAEAWGRADCGFTSESYQQGVLSATILYEILLGKATLYAVLVSALVLMAMVKRKDSRG
β11	79	YGLSENDEWTQDRAKPVTQIVSAEAWGRADCGFTSESYQQGVLSATILYEILLGKATLYAVLVSALVLMAMVKRKDSRG

β12	72	EWTQDRAKPVTQIVSAEAWGRADCGFTSESYQQGVLSATILYEILLGKATLYAVLVSALVLMAMVKRKDSRG
β13	66	AKPVTQIVSAEAWGRADCGFTSESYQQGVLSATILYEILLGKATLYAVLVSALVLMAMVKRKDSRG

Table S9. Primer sequences for inverse PCR

Construct	Forward Primer	Reverse Primer
α2	TACCAGCTGAGAGACTCTAAATCC	AGCCATGCTAAATTCAAGACAGGTG
α3	TCTGTCTGCCTATTCACCGATTTTG	
α4	AATGTGTCACAAAGTAAGGATTCTGATGTG	
α5	GATGTGTATATCACAGACAAAACTGTGCTAG	
α6	CTAGACATGAGGTCTATGGACTTCAAG	
α7	AAGAGCAACAGTGCTGTGGCC	
α8	AGCAACAAATCTGACTTTGCATGTG	
α9	TGTGCAAACGCCTTCAACAACAG	
α10	TTCAACAACAGCATTATTCCAGAAGAC	
α11	ATTCCAGAAGACACCTTCTTCCCC	
β2	TCAGAAGCAGAGATCTCCCACACCC	ACTGAACACAGAGCCTAGTCCCAG
β3	GCCACACTGGTGTGCCTGGCC	
β4	GTGGAGCTGAGCTGGTGGGTG	
β5	GTGAATGGGAAGGAGGTGCACAGTG	
β6	GTCAGCACAGACCCGCAGCCCC	
β7	GCCCTCAATGACTCCAGATACTGCC	
β8	CGCCTGAGGGTCTCGGCCAC	
β9	CAGAACCCCCGCAACCACTTCC	
β10	TTCCGCTGTCAAGTCCAGTTCTACG	
β11	TACGGGCTCTCGGAGAATGACG	
β12	GAGTGGACCCAGGATAGGGCCAAACC	
β13	GCCAAACCTGTCACCCAGATCGTC	
